# Fabrication of azido-PEG-NHC stabilized gold nanoparticles as a functionalizable platform†

**DOI:** 10.1039/d4sc04112g

**Published:** 2024-09-26

**Authors:** Constantin Eisen, Bernhard K. Keppler, Jia Min Chin, Xiaodi Su, Michael R. Reithofer

**Affiliations:** a Institute of Inorganic Chemistry, Faculty of Chemistry, University of Vienna Währinger Straße 42 1090 Vienna Austria michael.reithofer@univie.ac.at; b Institute of Materials Research and Engineering (IMRE), Agency for Science, Technology and Research (A*STAR) 2 Fusionopolis Way, Innovis #08-03 Singapore 138634 Singapore xd-su@imre.a-star.edu.sg; c Department of Functional Materials and Catalysis, Faculty of Chemistry, University of Vienna Währinger Straße 42 1090 Vienna Austria jiamin.chin@univie.ac.at

## Abstract

Rapid and precise detection of biochemical markers is vital for accurate medical diagnosis. Gold nanoparticles (AuNPs) have emerged as promising candidates for diagnostic sensing due to their biocompatibility and distinctive physical properties. However, AuNPs functionalized with selective targeting vectors often suffer from reduced stability in complex biological environments. To address this, (N)-heterocyclic carbene (NHC) ligands have been investigated for their robust binding affinity to AuNP surfaces, enhancing stability. This study outlines an optimized top-down synthesis route for highly stable, azide-terminal PEGylated NHC (PEG-NHC) functionalized AuNPs. This process employs well-defined oleylamine-protected AuNPs and masked PEGylated NHC precursors. The activation and attachment mechanisms of the masked NHCs were elucidated through the identification of intermediate AuNPs formed during incomplete ligand exchange. The resulting PEG-NHC@AuNPs exhibit exceptional colloidal stability across various biologically relevant media, showing no significant aggregation or ripening over extended periods. These particles demonstrate superior stability compared to those synthesized *via* a bottom-up approach. Further functionalization of azide-terminal PEG-NHC@AuNPs was achieved through copper-catalyzed click- and bioorthogonal strain-promoted azide–alkyne cycloaddition reactions. The maintained colloidal stability and successful conjugation highlight the potential of azide-functionalized PEG-NHC@AuNPs as a versatile platform for a wide range of biomedical applications.

## Abstract 2

Azid- and methoxy-functionalized PEG-NHC-CO_2_ adducts enable PEGylated NHC@AuNPs fabrication from oleylamine-protected AuNPs. N_3_-terminated NHC@AuNPs allow further derivatization *via* copper-catalyzed click or strain-promoted azide–alkyne cycloaddition.

## Graphical abstract

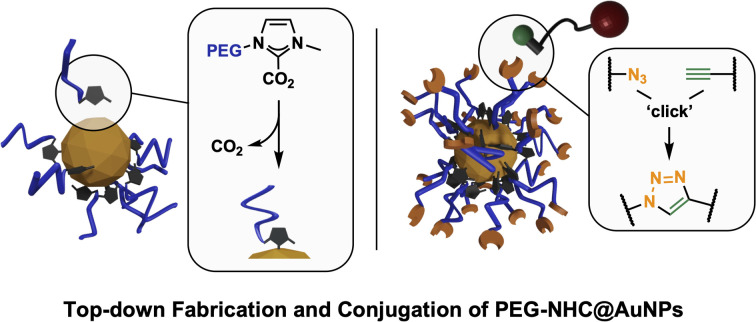

## Introduction

The rapid and selective detection of biochemical markers remains a fundamental quest in (bio-) medical diagnosis and sensing. In this context, gold nanoparticles (AuNPs), being biocompatible and featuring distinct (photo-) physical properties, are one of the most common motifs.^1^ State-of-the-art AuNP systems decorated with selective targeting vectors allow high-precision detection of biomolecules but often lack straightforward preparation and long-term stability in complex analytes.^2^ To improve stability in biological media and simplify target vector installation, robust and versatile post-synthetically modifiable AuNP platforms are desired.^3^

Nitrogen (N)-heterocyclic carbenes (NHCs) have been found to be excellent persistent surface ligands for metallic NPs, forming strong and inert bonds to the NP surfaces.^4^ Furthermore, the synthetic versatility of NHC precursors facilitates the synthesis of highly stable and multifunctional NHC@AuNPs.^5^ The synthesis of such stable NHC@AuNPs can be achieved by two major pathways: (1) bottom-up (BU) by the reduction of NHC-Au complexes or gold-containing imidazolium (IMZ) salts^6^ and (2) top-down (TD) *via* the exchange of weakly-coordinated ligands with activated NHCs or the deposition of NHC-Au complexes.^7^ The bottom-up approach benefits from a single reduction step but often lacks precise control over the final size of the resulting AuNPs. NHC@AuNPs obtained by a top-down approach benefit from tailor-made precursor AuNPs with precise size and shape, but successful ligand exchange with NHCs can be challenging as the exchange reaction can be sensitive to environmental conditions. Top-down approaches typically comprise of procedures utilizing either strong bases to generate NHCs, the deposition of NHC-Au complexes or through the use of NHC-silver/copper complexes as NHC transfer agents.^8^ Of note is the use of masked NHCs including the NHC-CO_2_ adduct (NHC-CO_2_) and IMZ hydrogen carbonates (IMZ HCO_3_), which marks a mild top-down approach to generate reactive NHC species facilitating the ligand exchange with metallic surfaces.^9^

NHC@AuNPs exhibit remarkable stability in challenging biological media bearing high concentrations of naturally occurring thiols, a broad pH range, as well as oxidative environments, making them suitable for use in various applications.^10^ They can also be functionalized with amphiphilic, biocompatible polymers to endow further stability and processability in both aqueous media and organic solvents.^10*b*,*c*,*f*,*g*,11^ Furthermore, distinct conjugation groups can be implemented in the NHC design, facilitating post-synthetic modification of NHC@AuNPs towards their final application.^12^ Given the limited reports of biomedical applications utilizing the advantage of the strong NHC–AuNP bond,^7*b*,9*b*,13^ the development of a versatile NHC@AuNP platform with a straightforward fabrication protocol for biomedical applications would be of huge value in expanding this promising area.

The incorporation of amphiphilic polymers like poly(ethylene) glycol (PEG) into the NHC design improves NHC@AuNPs stability in biomedically relevant media. Johnson,^7*b*,10*f*^ Crudden,^10*b*^ Nazemi,^10*c*,11,12*c*^ and co-workers have successfully achieved this, obtaining colloidally stable PEGylated NHC@AuNPs (PEG-NHC@AuNP) by the bottom-up reduction of PEGylated NHC-gold complexes. However, top-down methods to install PEGylated NHCs on pre-prepared Au nanomaterials would grant access to different Au nanostructures and their differing physicochemical properties for specific applications.^1*b*,7*b*,14^

**Fig. 1:**
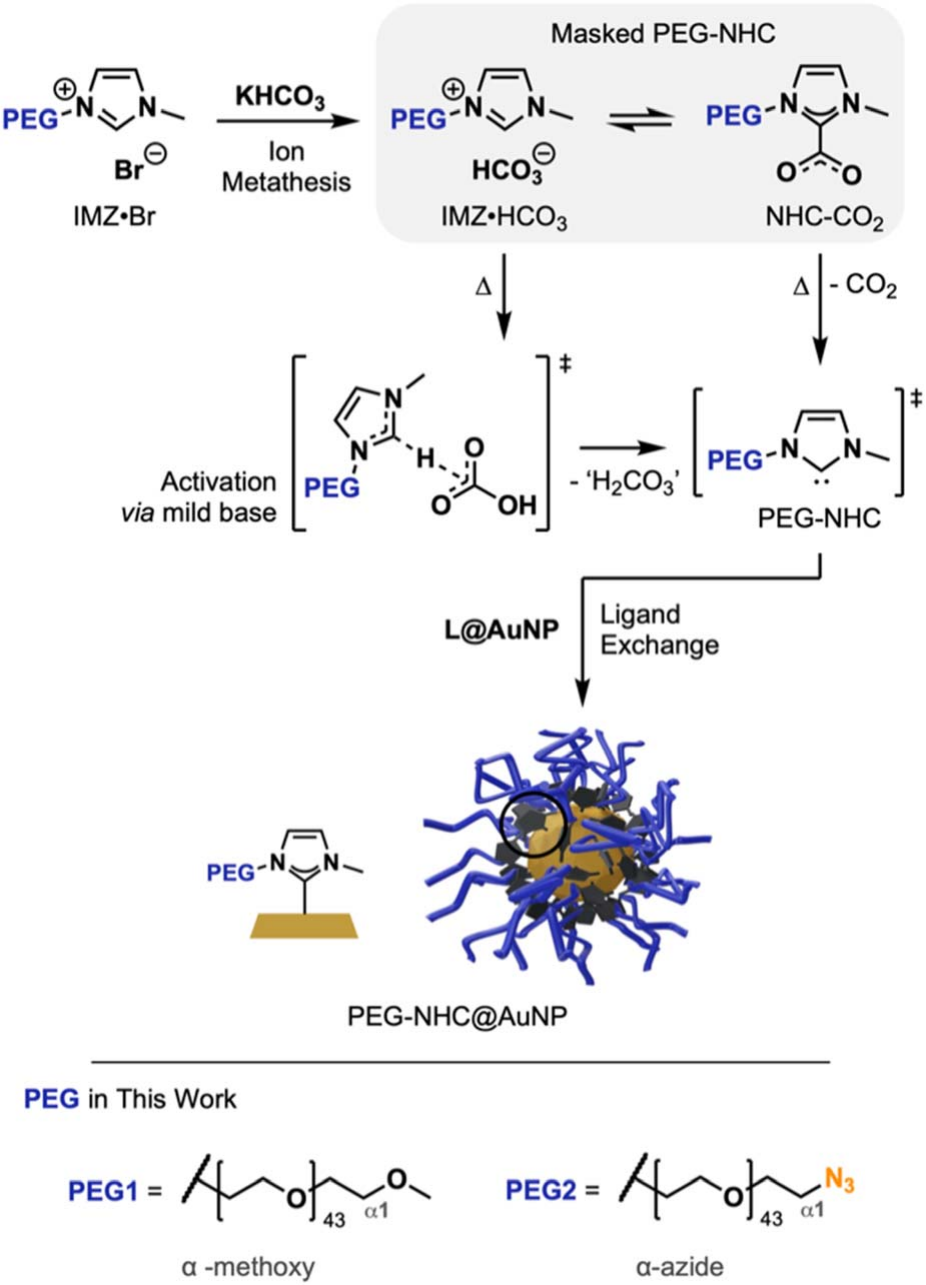
General reaction scheme towards PEG-NHC@AuNP using masked PEG-NHCs. Reaction progresses through ion metathesis, followed by the activation of the free PEG- and subsequent TD attachment to the AuNP surface.

To install structurally intact PEG-NHCs on AuNPs, a mild NHC activation approach is crucial to prevent damage to the NHC precursor structure. Masked NHCs as a mixture of IMZ HCO_3_ and NHC-CO_2_ offer a gentle way to generate active NHCs *via* the HCO_3_^−^ counterion as a mild base and the heat-induced liberation of masking CO_2_, respectively.^15^ Being synthetically facile and accessible through salt metathesis, masked NHCs are practical, air-stable precursors for complexations and Au surface modification (Fig. 1).^9*a*,*b*^

We describe here the successful synthesizes of conjugatable PEG-NHC@AuNPs *via* a top-down protocol. By using PEG with terminal azide modification to replace the oleylamine (OAm) coating on AuNPs, a PEG-NHC@AuNP platform is obtained, which not only grants broad stability in biomedically relevant conditions but also allows the post-synthesis addressability of these AuNPs by the versatile and bioorthogonal ‘click chemistry’ toolbox through alkyne cycloaddition. In order to install PEG-containing NHCs successfully on the AuNP surface and limit base-induced damage of the PEG chain, precursors undergoing mild NHC activation were used, and their mode of action was thoroughly characterized.

## Results and discussion

### Synthesis of PEGylated NHC precursors

The core structure of PEGylated NHC precursors were obtained by an alkylation procedure reported by Jadhav *et al.* using 1-methylimidazol with terminally brominated PEG species (av. *M*_n_ ∼2000 g mol^−1^; for synthesis and characterization of PEG1, α-methoxy and PEG2, with α-azide; refer to Fig. 1 and see ESI for synthesis details, Fig. S3†), yielding imidazolium bromides 1 (with PEG1 wingtip) and 2 (with PEG2 wingtip), respectively.^16^ Purified compounds were characterized by multinuclear nuclear magnetic resonance (NMR), Fourier-transformed infrared spectroscopy (FT-IR) and matrix-assisted laser desorption/ionization (Maldi-TOF). The results are in accordance with literature findings and confirmed the successful synthesis of 1 and azide-containing 2 without decomposition of the PEG chain and α-terminal azide group.^16,17^

Imidazolium bromides (IMZ Br) 1 and 2 were dissolved in methanol (MeOH) and potassium bicarbonate (KHCO_3_) was added. Stirring at 35 °C yielded the respective masked NHC precursors 1′ and 2′ as a mix of PEGylated IMZ HCO_3_ and corresponding NHC-CO_2_ adduct at a ratio of ∼3 : 2 based on integration of the ^1^H-NMR spectrum (in *d*_6_-DMSO, see ESI for details, Fig. S4 and 5†). The observed ratios are in accordance with previously published NMR data.^15^ The identification of masked NHC precursors *via* NMR, as well as the presence of additional HCO_3_^−^/CO_2_ signals in the region of 1680–1620 cm^−1^ in the FT-IR spectra, further proves the successful synthesis of the precursors (see ESI for spectra, Fig. S6–8†).^18^ Moreover, the integrity of the PEG chain was confirmed by Maldi-TOF (see ESI for spectra, Fig. S79–84†).

In parallel, imidazolium bromides were converted *via* transmetallation using silver(i) oxide (Ag_2_O) and chloro(dimethyl sulfide)gold(i) ([Au(DMS)Cl]) into the NHC-Au(i) complexes 1-Au (with terminal –OMe) and 2-Au (with terminal –N_3_) as precursors for the synthesis of PEG-NHC@AuNPs through a bottom-up approach. NHC gold complexes were obtained as a mixture of PEGylated NHC-Au(i) and NHC-Au(iii) complexes, as previously reported by Johnson and coworkers,^10*f*^ without any decomposition of the PEG chain nor loss of the α-azide moiety in case of 2-Au.

### Optimization of top-down fabrication of PEG-NHC@AuNPs

**Fig. 2:**
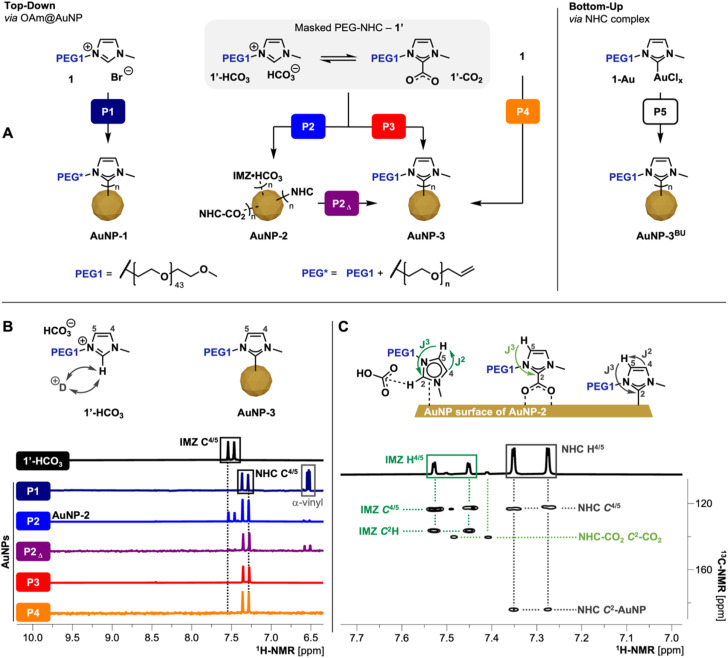
(A) Scheme of different TD ligand exchange and BU procedures; for synthesis details of presented procedures P1–5 refer to Table 1. P1 proceeds through the formation of free NHCs species using KHMDS, resulting in anchored NHCs but damaged PEG chain (PEG*); P2 and P3 utilizes 1′ as mild NHC precursors under heating; P2 utilizes 1′ as precursors at RT resulting in AuNP-2; P2_Δ_ heating of AuNP-2 allowing the conversion of surface bound IMZ HCO_3_/NHC-CO_2_ species to the respective coordinated PEG-NHCs; P4 one-pot approach *via*1 and KHCO_3_; (B) ^1^H-NMR (500 MHz, D_2_O) comparison of precursor 1′ (IMZ HCO_3_ state) with AuNPs obtained by procedures P1–P4; spectra display the respective imidazole-ring backbone protons which reveals the different states of coordination (see ESI for full spectra, Fig. S9†); fast H/D exchange cause disappearance of C^2^H signal of IMZ HCO_3_ species; (C) ^1^H/^13^C-HMBC (500 MHz, D_2_O) of AuNP-2 obtained by P2 revealing the coordination of IMZ HCO_3_/NHC-CO_2_/NHC species on the AuNP surface.

For the optimization of the TD approach (Fig. 2A), imidazolium salts 1 and the corresponding masked NHC 1′ were employed as a model system to mitigate unwanted side reactions caused by the azide moiety in 2 and 2′, respectively. As precursor AuNPs, AuNPs with loosely bound oleylamine (OAm@AuNPs, see ESI† for synthesis details) were used. Ligand exchange processes as well as PEG-NHC@AuNPs purified by dialysis against H_2_O were closely investigated by multinuclear NMR spectroscopy, FT-IR, X-ray photoelectron spectroscopy (XPS) analysis, ultraviolet-visible (UV-Vis) spectroscopy and transmission electron microscopy (TEM).

Initially, a routine TD approach using potassium bis(trimethylsilyl)amide (KHMDS) as a strong base to generate the free NHC was used. Activation of 1 was followed by the addition of the respective free NHC to OAm@AuNPs dispersed in THF, facilitating ligand exchange (procedure P1; Fig. 2A).^12*b*,19^ Using 1 as NHC precursor yielded AuNP-1 upon purification. Characterization by NMR, FT-IR, and XPS of obtained AuNPs reveals successful coordination of PEG-NHCs on the NP surface but also base-induced damage of the PEG chain. Such AuNP-1 display partial α-terminal vinyl groups, suggesting base-triggered elimination of the α-methoxy PEG terminus.^20^ Due to the observed polymer damage, we concluded that procedure P1 is not suitable for clickable ligand 2 containing AuNPs due to the potential loss of the α-terminal azide unit. The observed incompatibility of the PEG chains to the strong base used in the exchange reactions led us to employ a milder TD approach using corresponding masked NHC precursor 1′ (procedure P2; Fig. 2A). The addition of 1′ to dispersed OAm@AuNPs at room temperature (RT) yielded AuNP-2. Such AuNPs show reduced stability of the attached ligand shell, suggesting detachment of loosely bound PEG species and causing subsequent partial aggregation of AuNPs in dispersion. Detailed NMR investigations of AuNP-2 in deuterium oxide (D_2_O) obtained *via*P2 show the presence of IMZ HCO_3_1′-HCO_3_, NHC-CO_2_1′-CO_2_ as well as the desired NHC 1^NHC^ on the AuNP surfaces (Fig. 2C) indicating an incomplete transformation of 1′ to 1^NHC^ during the reaction. As 1′-HCO_3_ is less easily activated than 1′-CO_2_, this transformation is dependent upon two processes: (1) the initial conversion of 1′-HCO_3_ to 1′-CO_2_, and (2) CO_2_ loss from 1′-CO_2_ to yield the unmasked 1^NHC^.

As the equilibrium of the IMZ HCO_3_/NHC-CO_2_ pairs are affected by the presence of H_2_O,^15,21^ we investigated the equilibrium of masked NHCs *via*^1^H-NMR spectroscopy of 1′ in different deuterated solvents. While dry aprotic *d*_6_-DMSO stabilizes the equilibrium between IMZ HCO_3_/NHC-CO_2_ (ratio ∼3 : 2 by integration of the –C^6^H_3_ group), the ^1^H-NMR spectra recorded in D_2_O shows the equilibrium pushed to IMZ HCO_3_ with minimal contributions of NHC-CO_2_ (based on the identification of respective imidazole backbone protons C^4/5^H). Furthermore, the basicity of HCO_3_^−^ counterion in D_2_O leads to a rapid H/D exchange of the imidazolium proton (C^2^–H) and the corresponding disappearance of the signal in the ^1^H-NMR spectra (for spectra see ESI, Fig. S4 and 5†).

These shifts in equilibrium between the NHC-CO_2_ and IMZ HCO_3_ demonstrates why the masked NHC is not activated to form only the desired NHC species on AuNPs when used in protic or non-absolute solvents. Ligand exchange reactions in such conditions allow only the exchange of pre-installed ligands with IMZ HCO_3_ and small contributions of NHC-CO_2_, resulting in AuNPs which do not possess the same properties as AuNPs stabilized solely by NHCs. Furthermore, this also points out the importance of carefully controlling any moisture level during the top-down fabrication of NHC@AuNPs *via* masked NHCs.

Comparing the ^1^H-NMR spectra of P1-prepared AuNP-1 recorded in D_2_O with 1′, NHC backbone signals (C^4/5^H) of AuNP-1 are high field shifted by 0.18 ppm (see Fig. 2B). Both backbone signals show cross peaks with the NHC C^2^ signal in ^1^H/^13^C heteronuclear multiple bond correlation (^1^H/^13^C-HMBC), clearly identifying the key components of the imidazole ring of NHC anchored to AuNPs. In contrast, ^1^H-NMR of AuNP-2 prepared *via*P2 shows a mixture of ligands on the surface. One set of backbone signals observed in the ^1^H-NMR corresponds to the NHC structure, like previously shown for P1 and the second set in the lower field corresponds to an IMZ species (for ^1^H-NMR see Fig. 2B). The absence of the IMZ proton (C^2^H) suggests the presence of masked NHC from 1′ as precursor undergoing insufficient NHC activation.

Conversely, the ratio of observed surface species (1^NHC^/1′-HCO_3_/1′-CO_2_, 6 : 3 : 1; Fig. 2C) indicated that the NHC-CO_2_ adduct is the major reactive component of the precursor 1′ under ambient conditions as previously suggested by He and co-workers.^22^

Based on the observed incomplete activation of 1′ while employed in P2, obtained AuNP-2 were redispersed in THF and heated to 40 °C for 62 h. Heating AuNP-2 (procedure P2_Δ_) leads to the activation of remaining loosely coordinated species including IMZ HCO_3_ and NHC-CO_2_ species and converts them into the desired NHC species firmly attached to the AuNP surface. ^1^H-NMR of the heated sample shows full conversion of IMZ HCO_3_ species (IMZ related backbone signals; see Fig. 2B) into the desired NHC bond to the surface as well as changes in N 1s and Au 4f spectra to AuNPs gained by procedure P1 (see ESI for XPS comparison, Fig. S10†).

Subsequently, an alternative exchange route was tested whereby the masked NHC 1′ was mixed with OAm@AuNPs and heated to 40 °C for 62 h (procedure P3), yielding upon purification stable AuNP-3 covered in the desired PEG-NHC 1^NHC^. To further simplify the ligand exchange procedure, the ion metathesis to generate 1′ was combined with the ligand exchange in a ‘one-pot’ approach. The developed procedure P4, uses the imidazolium bromide 1 and KHCO_3_ in OAm@AuNP dispersed in THF, *in situ* generating masked NHC and which is concomitantly activated at 40 °C to generate AuNP-3 stabilized by 1^NHC^.

NMR, FT-IR, and XPS spectroscopic evidence show that procedures P2 (followed by P2_Δ_), P3, and P4 all lead to full conversion of 1′ into Au surface-bound NHCs. However, the different procedures yield particles with significantly different colloidal quality. While AuNP-3 obtained by P2 and P4 have lower colloidal stability and broader dispersity, AuNP-3 obtained by procedure P3 stand out with higher colloidal stability and minimal decomposition of AuNPs during the exchange procedure (see ESI for UV-vis spectra, Fig. S11†).

Finally, procedures P3 and P4 were tested under aerobic conditions using non-anhydrous THF. All attempts failed and no stable AuNP-3 were isolated. Obtained AuNPs decomposed during the transfer in aq. conditions or during dialysis, suggesting incomplete ligand exchange or only loosely-bound IMZ species on the AuNP surface. We attribute this to the H_2_O content of THF used, which drives the equilibrium of the masked NHC 1′ towards the less active IMZ HCO_3_ species. Simultaneously, H_2_O also promotes the rapid protonation of free 1^NHC^ generated.

### Characterization of PEG-NHC@AuNPs

**Fig. 3:**
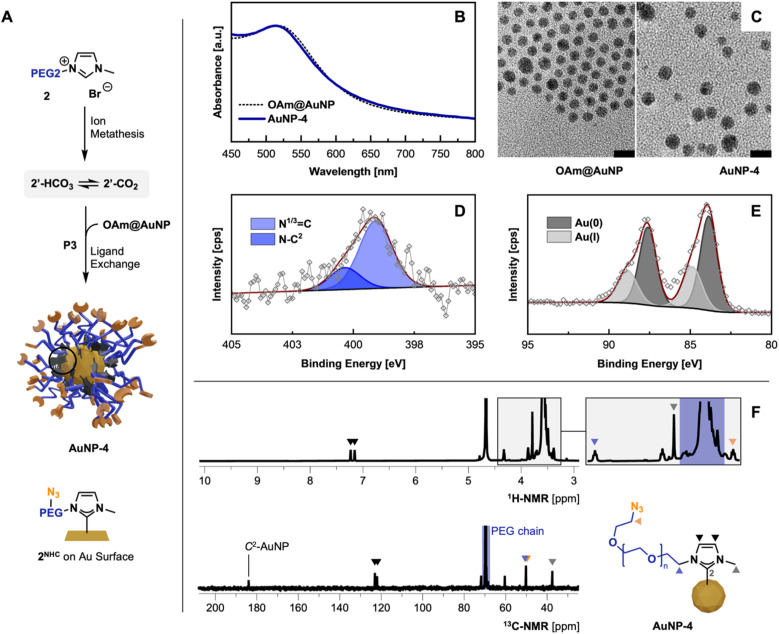
(A) Optimized synthesis of AuNP-4 following P3; ion metathesis – KHCO_3_, MeOH, 35 °C, 48 h; P3 THF, 40 °C, 62 h; characterization of AuNP-4 obtained *via* ligand exchange procedure P3: (B) UV-Vis comparison against OAm@AuNPs in THF; (C) TEM micrographs comparison (scale bar 10 nm); (D and E) high-res. XPS spectra of N 1s and Au 4f region; (F) ^1^H- and ^13^C-NMR (500 MHz, D_2_O) and structural assignment.

Following the optimized route P3, AuNP-3 and AuNP-4 were synthesized using precursors 1′ and azide containing 2′. The absence of the imidazolium proton (C^2^H) in the ^1^H-NMR spectra in combination with the appearance of the C^2^ signal at 184.0 ppm in ^13^C-NMR indicated the successful carbene formation (Fig. 3F).^10*f*,23^ All other NMR signals can be assigned to the remaining structure of the NHC anchor. Subsequently, FT-IR measurements of thin films of AuNP-3 and AuNP-4 show the absence of the imidazolium ring stretch (N^1^–C^2^–N^3^) signal at 1570 cm^−1^, underlining the successful installation of NHCs on the AuNPs surface.^10*f*,24^ In the case of AuNP-4 the IR band associated with the terminal azido group – while compared to the employed 2′ precursor – remained intact (for comparison see ESI, Fig. S8†).

XPS analysis of obtained AuNPs confirms the structural features identified in previous analyses and is consistent with previously reported data by Nazemi, Crudden, and coworkers.^10*b*^

C 1s scans show contributions of C–C/C–H as well as the PEG-associated C–O–C ether bonds at 284.8 eV and 286.4 eV, respectively (see ESI for XPS spectra, Fig. S13 and 14†). Comparing XPS spectra of PEGylated AuNPs and their precursor OAm@AuNP, distinct differences in the N 1s and Au 4f scans are observable, indicating successful NHC anchoring to the AuNP surface. The observed peak in the N 1s scan of AuNP-3 and AuNP-4 can be separated in the N–C^2^ structure associated with the NHC(C^2^)–AuNP bond and the quaternary N (N^1/3^=C) contributions from the nitrogen connecting the NHC wingtips at 400.4 and 399.1 eV.^25^ Distinct contributions of the azido group at ∼405 eV in AuNP-4 were not detectable due to the lability of the azide groups during XPS measurements (Fig. 1D).^26^ High-resolution Au 4f scans show Au(0) and Au(i) contributions (see Fig. 3E), similar to previously reported XPS data obtained from bottom-up synthesized NHC@AuNPs.^10*c*,24,27^ Observed peaks at 88.3 and 84.6 eV in the case of AuNP-3 suggest the presence of Au(i), as they are shifted by *Δ*_BE_ = 1.2 eV compared to Au(0) components (Au 4f_5/2_ 87.1 eV and Au 4f_7/2_ 83.4 eV; see ESI for detailed comparisons, Fig. S10†).^28^ Interestingly, the presence of Au(i) suggests that during ligand exchange, the NHC coordinates onto the Au surface oxidatively, generating partially oxidized Au surface atoms or Au ad-atoms like that observed during the coordination of NHC-CO_2_ adducts to Au(111) surfaces in previous reports.^9*a*,29^ Such an ‘oxidative’ ligand exchange on AuNPs is – to the best of our knowledge – not reported for commonly used surface chemistry (*e.g.* amine^30^ or thiol^31^ based ligands).

The obtained red dispersions of AuNPs coordinated with 1^NHC^ and 2^NHC^ prepared by procedure P3 show a surface plasmon (SP) band at 515 nm in deionized water (H_2_O), and 510 and 515 nm for AuNP-3 and AuNP-4 in THF, respectively (for spectra see ESI, Fig. S22†). Comparing the SP band of obtained AuNPs with OAm@AuNPs (Fig. 3B; SP band, 515 nm) in THF, no significant change in the SP band is visible despite the observed size increase (by TEM) of the gold core of NHC@AuNPs. This is attributed to the change in refractive index of the AuNP system. Due to the anchoring of a thick PEG shell, slight peak broadening is observed which is a common observation upon PEGylation of AuNPs.^32^ TEM micrographs of AuNP-3 and AuNP-4 reveal spherical particles with an average diameter (*d*) of ∼4.6 nm showing minor ripening compared to precursor OAm@AuNPs (*d*, ∼4.4 nm; Fig. 1C). DLS measurements of PEG-NHC@AuNP in H_2_O result in a hydrodynamic diameter of ∼15 nm. Comparing DLS results against TEM micrographs allows the estimation of the PEG shell thickness (∼4–5 nm). The observed thickness of the PEG layer indicates partial extension of utilized PEG chains (calcd. PEG_2000_ length ∼12–13 nm in H_2_O)^33^ and a densely packed PEG brush conformation on the AuNP surface.^34^ The dense PEG coverage was further confirmed by thermogravimetric analysis showing an organic contribution of 69 wt% for AuNP-4. Measured zeta (*ζ*) potentials of PEG-NHC@AuNPs in the neutral range (±10 mV) indicates successful PEG-NHC installation (for values see Table 2).^32,34*a*^

**Table 1:** List of all AuNP synthesis procedures

	Precursor	Reagent	Temperature [°C]	NHC activation mode
P1	1	KHMDS	40	Strong base
P2	1′	—	25 (RT)	Mild base
P2_Δ_	AuNP-2	—	40	
P3	1′	—	40	
P4	1	KHCO_3_	40	*In situ* mild base
P5^a^	1-Au	*t*BuNH_2_·BH_3_	25 (RT)	N/A

a In DCM. All other entries in THF.

**Table 2:** Overview of obtained AuNP diameters (TEM and DLS) and respective *ζ*-potentials

Ligand@AuNP	*d* (TEM) [nm]	*d* (DLS) [nm]	*ζ* potential [mV]
OAm	4.4 ± 0.4	9.1 ± 2.0^a^	—
AuNP-3	4.6 ± 0.7	15.2 ± 3.4	−9.6
AuNP-4	4.6 ± 0.7	14.6 ± 2.8	2.8

a Measured in THF. All other DLS and *ζ*-potentials in H_2_O.

### Stability studies of PEG-NHC@AuNPs

PEG-NHCs as surface ligands for Au nanomaterials have shown increased stabilizing performance in various biomedically relevant conditions, including buffer solutions of a wide range of pH values, oxidative conditions, and the exchange with exogenous natural and PEG-based thiols (PEG-SH).^7*b*,10*c*,10*f*,27*c*^

In this study, AuNP-3 were exposed to a series of conditions and retained colloidal stability in aq. media and easy handling in various organic solvents. In terms of biologically relevant conditions, AuNP-3 were dispersed in 1× PBS (a common physiological buffer), 0.6 M NaCl (a high ion solution), HCl solution (pH 2), NaOH solution (pH 12), 1 M H_2_O_2_ (an oxidative environment), fetal bovine serum (FBS, a complex media for cell culture) and 3 mM glutathione (GSH, a model system for natural occurring thiols). Monitoring the SP band of AuNP-3 over a period up to 7 days revealed minimal to no changes in most of these conditions, showing retained colloidal stability on par with previously published systems (for UV-Vis spectra see ESI; Fig. S26 and 27†).^7*b*,10*c*,10*f*^ Interestingly, AuNP-3 shows a sharpening of the SP band while exposed to H_2_O, 1× PBS, and NaCl (0.6 M) over the course of 7 days, indicating the slow dispersion of small aggregates caused by previous *in vacuo* drying steps or rearrangement of surface-bond PEG chains.^35^AuNP-3 exposed to 3 mM PEG-SH (*M*_n_ = 2000 g mol^−1^) shows a red shift (*Δ*_nm_ ∼3 nm) and peak broadening.

Unlike AuNP-3 that shows no or limited SP shift under all conditions, AuNP-4 only shows comparably retained stability in H_2_O, 1× PBS, at pH 2/12, H_2_O_2,_ and GSH tests, but decreased stability in NaCl (0.6 M), FBS and against PEG-SH, as indicated by the red-shifts and broadening of the SP band (for UV-Vis spectra see ESI; Fig. S30 and 31†). To further confirm the stability of AuNP-3 and azide-terminated AuNP-4 under certain conditions over the period of 7 days, TEM micrographs were taken after the exposure to 1× PBS, H_2_O_2,_ and PEG-SH. TEM images confirm the retention of size and shape in the case of 1× PBS for AuNP-3 and AuNP-4 (*d*, ∼4.9 nm). In the case of oxidative conditions or the presence of excess PEG-SH, AuNP-3 and AuNP-4 show ripening and morphology changes as indicated by previous UV-Vis studies. The size increase observed by TEM correlates well with the observed red shift of the respective SP bands (for TEM micrographs see ESI; Fig. S27 and S31†). To verify that during stability studies no ligand exchange occurs, both AuNP-3 and AuNP-4 were exposed to H_2_O_2_ (1 M) and GSH (3 mM) and monitored by ^1^H-NMR, showing, the resilience of surface bound PEG-NHCs despite observed shifts of the SP band and morphology changes by TEM (see ESI for ^1^H-NMR, Fig. S28, 29, S32 and 33†).

### Comparison of PEG-NHC@AuNPs obtained by top-down and bottom-up approach

Through an established bottom-up protocol,^10*f*^ NHC gold complexes 1-Au (with terminal-OMe) and 2-Au (with terminal-N_3_) were reduced to the corresponding AuNP-3^BU^ and AuNP-4^BU^ (Fig. 2A) allowing direct comparison with NPs obtained by the TD approach. Characterization of AuNP-3^BU^ and AuNP-4^BU^ by ^1^H-NMR and XPS reveals a comparable composition when compared to AuNP-3 and AuNP-4, respectively (see ESI for ^1^H-comparison, Fig. S12†). Despite these comparable chemical compositions, AuNPs obtained from the BU synthesis showed rapid ripening during mild heat exposure (40 °C) as well as after drying and redispersion. Similar to reports by Crudden and coworkers,^36^AuNP-3^BU^ and AuNP-4^BU^ obtained directly after purification by dialysis only showed a very weak and broad SP band indicating particles with a diameter <3 nm (for UV-Vis see ESI, Fig. S37†). Subsequently, TEM micrographs obtained by drying of AuNP-3^BU^ and AuNP-4^BU^ on the TEM grid show a broad size distribution with distinct populations at ∼1.5 and ∼5 nm. The presence of larger NPs is attributed to digestive ripening during concentrating and drying (40 °C) for TEM grid preparation. Identical behaviour is observed during careful drying *in vacuo* followed by redispersion which can be followed by UV-Vis spectroscopy through the appearance of a corresponding SP band at ∼510 nm. Exposing aq. dispersions of AuNP-3^BU^ and AuNP-4^BU^ obtained after dialysis to 40 °C for 4 h, causes further ripening indicated by a red shift of SP band to ∼520 nm. In contrast, AuNP-3 and AuNP-4 obtained by the TD route P3 treated under identical conditions show no changes of the SP band.

Redispersing AuNP-3^BU^ and AuNP-4^BU^ in 1× PBS, GSH (3 mM) and H_2_O_2_ (1 M) further confirms the lower colloidal stability when compared with their counterparts obtained by procedure P3.

### Conjugation *via* click chemistry

After confirming successful PEG-NHC installation on the AuNP surface and requisite stability assessment, the utility of AuNP-4 as a conjugatable platform was tested. In order to facilitate rapid and selective conjugation and prove the addressability of the available terminal azide group of AuNP-4, two routine click chemistry approaches were employed,^37^ namely (1) copper-catalyzed azide–alkyne cycloaddition (CuAAC)^38^ and (2) strain-promoted azide–alkyne cycloaddition (SPAAC)^39^ reactions.

**Fig. 4:**
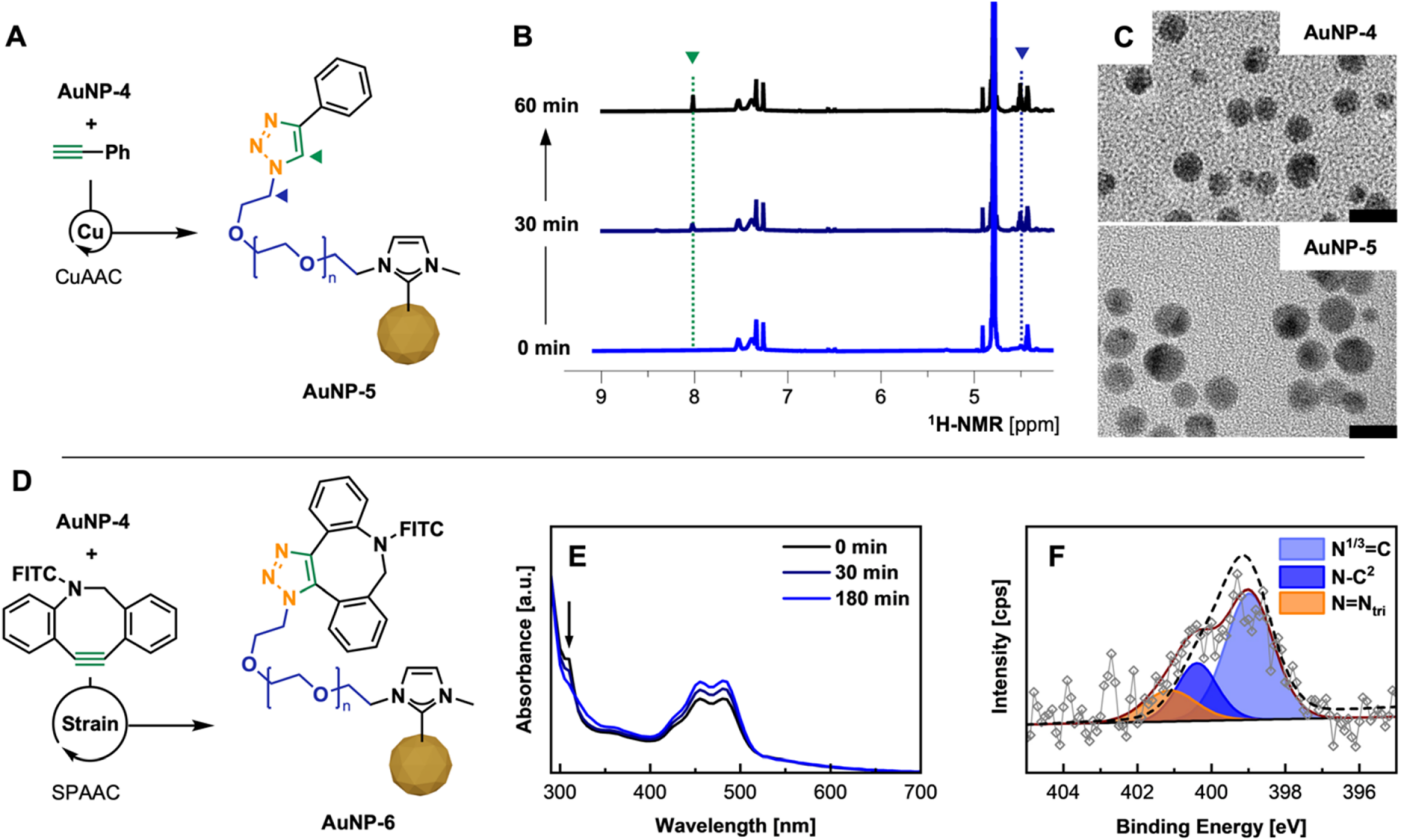
(A) CuAAC of AuNP-4 and phenylacetylene using Cu(i)/THPTA as catalyst; (B) NMR kinetics showing the formation of triazole by the appearance of C^7^H (green) and C^α1^H_2_ (blue) over the 60 min (for details see ESI; Fig. S40†); (C) TEM micrographs of AuNP-4 and AuNP-5 after CuAAC (scale bar 10 nm). (D) SPAAC of AuNP-4 with DBCO-FITC; (E) UV-Vis kinetics allowing the tracking of the click reaction by the consumption of DBCO (band at 310 nm); (F) high-res. XPS spectrum of N 1s region of purified AuNP-6 including envelope of AuNP-4 (---) visualizing peak change.

CuAAC experiments were conducted under aerobic conditions in a mixture of H_2_O/MeOH, using 5 mol% Cu loading, with tris(3-hydroxypropyltriazolylmethyl)amine (THPTA) as a robust and water soluble Cu(i) ligand and phenylacetylene as alkyne.^40^ Upon mixing all components followed by the reductive-activation of Cu(ii) with l-ascorbic acid,^38*a*,41^ the reaction progress was monitored by ^1^H-NMR *via* the appearance of the triazole C^7^–H proton as well as a low field shifted signal for the C^α1^H_2_ protons at 8.00 and 4.50 ppm (Fig. 4B).^10*c*,42^ Despite the poor solubility of phenylacetylene in given reaction conditions, ^1^H-NMR kinetics suggest rapid conversion underlining the facile addressability of accessible α-terminal N_3_ groups incorporated in the polymer shell of AuNP-4, yielding clicked AuNP-5.

We then moved on to the bioorthogonal SPAAC approach, which grants easier handling and reduces the reaction components to AuNPs and strained-alkyne modified biomolecules.^43^ SPAAC reactions were scaled down to perform the reaction in well plates common for assay-based studies.

Described SPAAC experiments were conducted by mixing solutions of AuNP-4 with fluorescein-DBCO^44^ (FITC-DBCO; ratio of N_3_/DBCO of 2 : 1) in H_2_O/EtOH. The process of the conjugation was monitored by UV-vis through the decrease of the DBCO specific absorbance band at 310 nm over a period of 3 h (Fig. 4E) and further confirmed by FT-IR (see ESI for spectrum, Fig. S44†) and XPS.^45^ Disappearance of the band confirmed the full conversion of the free DBCO moiety in the corresponding triazole species attached to the PEG shell of AuNPs. Subsequent fluorescence emission spectra of such conjugated AuNP-6 show a clear emission at 524 nm compared to unconjugated AuNP-4, which does not show any fluorescence when exited at 460 nm (see ESI for spectra; Fig. S43†). Comparing the signal intensity of conjugated FITC to FITC-DBCO at the initially used concentration, a significant emission signal enhancement by Förster resonance energy transfer (FRET) is observed, which is supported by the structural features of AuNP-6 (AuNP *d*_TEM_, ∼6 nm, ligand shell thickness 4–5 nm, see ESI for TEM, Fig. S46†) and the good overlap of the emission of FITC with the SP band of employed AuNPs (*λ*_max_, 515 nm).^46^

## Conclusion

Herein, we report a top-down approach for the synthesis of α-terminal azide PEG-NHC stabilized AuNPs, using well-defined OAm@AuNPs and a mild NHC activation strategy. The target AuNPs, PEGylated NHC@AuNP, were obtained upon a thorough optimization path by mixing the respective masked NHCs as precursors with OAm@AuNPs under mild heating (40 °C), where the masked PEGylated NHCs were activated but without structural damage. The released surface-reactive NHC species^15^ undergo ligand exchange with the OAm coating on the AuNP surface. The necessity for an aprotic environment and heat-induced activation of masked NHCs was shown through the isolation of AuNP-2 containing a mixture of loosely-bond IMZ HCO_3_, NHC-CO_2_ and the desired NHCs. Subsequently, heating of AuNP-2 facilitates the activation of the weakly bound species and their conversion in the respective surface-anchored NHCs.

The azide-containing PEGylated AuNPs AuNP-4 produced through this ligand exchange procedure retain colloidal stability in biologically relevant media and show greater stability when compared with their counterparts obtained by the BU synthesis. The presence of azide groups on AuNP-4 allows robust and versatile conjugation, employing two common click chemistry approaches (CuAAC and SPAAC). With its synthetic robustness, retained colloidal stability and bioorthogonal addressability, the presented PEG-NHC@AuNP platform offers a unique opportunity for its use in complex applications, *e.g.* biomedical sensing, diagnosis and delivery.

## Author contributions

M. R. R. designed the study. C. E. performed the experiments. C. E. and M. R. R. analyzed the data. M. R. R., J. C., B. K. K., and X. S. supervised the study. The manuscript was written through contributions of all authors. All authors have given approval to the final version of the manuscript.

## Conflicts of interest

There are no conflicts to declare.

## Supplementary Material

SC-015-D4SC04112G-s001

## Data Availability

Experimental and analytical data supporting this article are available in the ESI.†
