# Supplementary material for: Fabrication of azido-PEG-NHC stabilized gold nanoparticles as a functionalizable platform
Source: Chem Sci. 2024 Sep 26;15(44):18524–33. doi: 10.1039/d4sc04112g (PMC11487300; doi:10.1039/d4sc04112g)
Supplement: SC-015-D4SC04112G-s001 [file SC-015-D4SC04112G-s001.pdf]

Supplementary Information (SI) for Chemical Science.
This journal is © The Royal Society of Chemistry 2024

Electronic Supporting Information

Fabrication of Azido-PEG-NHC stabilized Gold Nanoparticles as a functionalizable Platform

Constantin Eisen[a,b], Bernhard K. Keppler[a], Jia Min Chin[c], Xiaodi Su[b]* and Michael R. Reithofer[a]**

[a] Institute of Inorganic Chemistry, Faculty of Chemistry, University of Vienna Währinger Straße 42, 1090 Vienna, Austria E-mail: michael.reithofer@univie.ac.at

[b] Institute of Materials Research and Engineering (IMRE) / Agency for Science, Technology and Research (A*STAR) 2 Fusionopolis Way, Innovis #08-03, Singapore 138634, Singapore, Email: xd-su@imre.a-star.edu.sg

[c] Department of Functional Materials and Catalysis, Faculty of Chemistry, University of Vienna, Währinger Straße 42, 1090 Vienna, Austria, Email: jiamin.chin@univie.ac.at

Table of Contents

Materials and Methods **3**
Synthesis of Brominated PEG **4**
Synthesis of PEGylated Imidazolium and NHC Precursors **4**
General Procedure for the Synthesis of Imidazolium Bromides 4
General Procedure for the Synthesis of Masked NHCs 5
General Procedure for the Synthesis of PEGylated NHC-Au complexes 6
Synthesis of Oleylamine stabilized AuNPs **6**
Synthesis of PEG-NHC@AuNPs *via* Top-down Approach **7**
Synthesis of PEG-NHC@AuNP *via* Procedure P1 7
Synthesis of PEG-NHC@AuNPs *via* Procedure P2 7
General Procedure for the Synthesis of AuNP-3 (P3) 8
Synthesis of PEG-NHC@AuNPs *via* One-pot Approach (P4) 9
General Procedure for the Synthesis of PEG-NHC@AuNP *via* Bottom-up Approach (P5) **9**
Setup of Stability Studies **9**
Conjugation of AuNPs *via* Click Chemistry **10**
Additional Figures **11**
General Reaction Scheme of Imidazolium Bromide Synthesis 11
^{1}H-NMR Comparison of Masked NHC 1' in Different Solvents 11
FT-IR Comparison of Precursors and AuNPs Obtained by TD Procedures 12
^{1}H-NMR Comparison of Imidazolium Precursor and AuNPs Obtained by TD Procedures 14
XPS Comparison of Precursors and AuNPs obtained by TD Procedures 14
Comparison of UV-Vis Spectra of AuNPs Obtained by Procedures P1-P4 15
Comparison of ^{1}H-NMR Spectra of OAm@AuNP and AuNPs from TD and BU Procedures 15
High Resolution XPS Spectra **16**
TEM Micrographs **18**
UV-Vis Spectra in THF and H_2O **19**
Dynamic Light Scattering **20**
Thermogravimetric Analysis and NHC Coverage **21**
Stability Studies – UV-Vis spectra, TEM micrographs and ^{1}H-NMR Spectra **22**
Characterization of Bottom-up AuNPs **28**
Characterization of Click Conjugation Procedures **32**
CuAAC 32
SPAAC 34
^{1}H- and ^{13}C-NMR Spectra **36**
High-resolution Mass Spectra **52**
References **54**

Caution: Presented research contains the synthesis of organic azides. While we did not encounter any difficulties, all azide-containing polymers were considered potentially hazardous due to potential explosive decomposition. Reactions should not be scaled-up without carefully evaluating their safety. Molecular samples were stored under the exclusion of light at 4 °C or a N_2 filled glovebox.

Materials and Methods

All experiments, if not stated otherwise, were performed using standard Schlenk technique or a nitrogen (N_2) filled MBraun MB-200B glovebox using dry solvents and flame dried glassware. Commercially available reagents were used without further purification. Dry solvents were obtained from Sigma Aldrich (THF, MeOH and toluene). Deuterated solvents ($CDCl_3$, D_2O and d_6-DMSO) were obtained by Cambridge Isotopes Laboratories. Ultra-pure water (18.2 MΩ.cm) was obtained by Elga PureLab Option-Q water purification system.

PEG1 and **PEG2** were synthesized according to literature[1] procedures, using MeO-PEG-OH (Sigma Aldrich, 202509, LOT MKCK9434, M_n ~2000) and HO-PEG-OH (TCI, P2034, LOT 5MZ7C-EE, M_n ~2000), respectively, as starting materials.

^1H-, ^{13}C- and *2D-NMR* spectra were recorded at 25 °C on a Jeol ECA500II FT NMR spectrometer at 500.32 MHz (^1H) and 125.81 MHz (^{13}C). Residual protic solvent peaks ($CDCl_3$ δ_{1H} = 7.26, δ_{13C} = 77.16; D_2O δ_{1H} = 4.69; d_6-DMSO δ_{1H} = 2.50, δ_{13C} = 39.52 ppm) were used as internal standard. Chemical shifts are given in ppm (δ) and coupling constants (J) are given in Hertz (Hz). Jeol DELTA NMR v5.0.4 was used to process and MestreNova v14.1.2 to analyse and visualize the NMR spectra.

Maldi-TOF measurements were conducted on a Bruker Autoflex Speed LRF (matrix DCTB for **PEG1**/**PEG2** and HCCA for **1**/**2** and **1'**/**2'**, laser power 15-24%) at the Mass Spectrometry Centre, Faculty of Chemistry, University of Vienna.

FT-IR were recorded on a Bruker Vertex 80v in solid form or thin films *in vacuo*. Opus v7.2 was used for data collection and processing.

UV-Vis spectra were recorded using a BioTek Synergy 2 plate reader and transparent polypropylene 96/384 well plates (Corning 3365). Spectra are recorded from 400-800 nm with a step size of 2 or 5 nm.

Fluorescence Emission spectra were recorded using a Tecan infinite M200 plate reader and black polystyrene 384 well plates (Greiner 781079). Spectra are recorded from 400-800 nm with a step size of 2 nm.

DLS and *Zeta Potential* were recorded of stable AuNP dispersions with a Malvern Zetasizer Nano ZS90.

XPS was performed on a Theta-Probe photoelectron spectrometer system (Thermo Fisher Scientific). Element specific high-resolution spectra for Carbon (C 1s 280-298 eV), Nitrogen (N 1s 395-405 eV) and Gold (Au 4f 80-95 eV) were recorded with step sizes of 0.1 eV and a pass energy of 40 eV at 1×10^{-9} mbar were acquired. All measurements were performed using monochromated and micro-focused Al-Kα X-rays (hv = 1486.6 eV) with a spot size of 400 µm. For Au 4f spectra etching was performed with monoatomic Ar (3 keV, 4x4 mm raster size). Samples were prepared by drop-casting samples in THF onto solvent cleaned silicon wafers (5x5 mm), followed by drying at 60 °C. Obtained spectra were evaluated using the Advantage software package v5.9922 provided by Thermo Fisher Scientific.

TEM was performed using a FEI TECNAI G2 F20 (200 kV) TEM equipped with a 2k×2k CCD camera from Gatan. Prior to measurement, dispersed samples were drop cast onto carbon film on 200 mesh copper grids and dried at 60 °C. TEM micrographs were analysed using Image J v1.53k.

TGA measurements were performed using a Netzsch Jupiter STA 449-F1 analyser. Measurements were performed under argon flow (RT-800 °C, rate 5 °C/min). Weight loss was recorded between 120-800 °C to remove contributions of residual solvents and H_2O. Samples were prepared by drying sample dispersion in a ceramic crucible at 60 °C.

Synthesis of Brominated PEG

Synthesis of PEG1

The synthesis protocol for **PEG1** was adapted from Velluto *et al.* with slight modifications.[1a] Pre-dried methoxy PEG-OH (M_n 2000 g/mol, 10.000 g, 5 mmol, 1 eq.) was dissolved in toluene (abs.,100 mL) and thionyl bromide (0.97 mL, 12.5 mmol, 2.5 eq.) was slowly added. The resulting mixture was heated to reflux for 16 h. After cooling to RT and concentration of the crude product under reduced pressure, Et_2O was added affording a white solid to principate from solution. Collection of the solid *via* centrifugation and subsequent drying *in vacuo* yields **PEG1** (9.348 g, 4.5 mmol, 90 %).

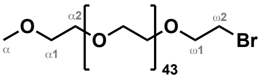

PEG1
$C_{91}H_{183}O_{45}Br$
2077.32 g/mol

^{1}H-NMR ($CDCl_3$, 500 MHz) δ = 3.77 (t, J = 6.4 Hz 2 H, $C^{\omega1}H_2CH_2Br$), 3.61 (broad s, 194 H, $(O(CH_2)_2)_{43}$), 3.52-3.50 (m, 2 H, $C^{\alpha1}H_2OMe$) 3.44 (t, J = 64 Hz, 2 H, $C^{\omega2}H_2Br$), 3.35 (s, 3 H, $OC^{\alpha}H_3$) ppm.
^{13}C-NMR ($CDCl_3$, 500 MHz) δ = 72.0 ($C^{\alpha1}H_2OMe$), 71.2 ($C^{\omega1}H_2CH_2Br$), 70.6 (broad, $(O(CH_2)_2)_{43}$), 59.1 ($OC^{\alpha}H_3$), 30.4 ($C^{\omega2}H_2Br$) ppm.
m/z (Maldi-TOF) Calcd for $C_{91}H_{183}O_{45}Br$ [M+nNa, $C_{91}H_{183}O_{45}BrNa^+$]: 2100.1119; Found: 2100.1119.

Synthesis of PEG2

The synthesis protocol for **PEG2** was adapted from Velluto *et al.*[1a] and Semple *et al.*[1b] with slight modifications. Pre-dried HO-PEG-OH (M_n 2000 g/mol, 10.000 g, 5 mmol, 1 eq.) was dissolved in toluene (abs., 100 mL) and thionyl bromide (1.93 mL, 25 mmol, 5 eq.) was slowly added. The resulting mixture was heated to reflux for 16 h. After cooling to RT and concentration of the crude product under reduced pressure, Et_2O was added causing the bi-brominated intermediate (**Br-PEG-Br**) to precipitate from solution. The solid was collected by centrifugation and dried *in vacuo*. **Br-PEG-Br** (8.000 g, 3.763 mmol, 1 eq.) was dissolved in EtOH (50 mL), sodium azide (0.269 g, 4.139 mmol, 1.1 eq.) was added and the resulting mixture was stirred at 60 °C for 16 h. The final mixture was cooled and filtered through a syringe filter (PTFE, pore size 0.4 µm). Removal of all volatiles yields **PEG2** (6.129 g, 2.935 mmol, 78%) and was used without further purification.

Characterization of Intermediate Br-PEG-Br

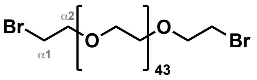

Br-PEG-Br
$C_{90}H_{180}O_{44}Br_2$
2126.19 g/mol

^{1}H-NMR ($CDCl_3$, 500 MHz) δ = 3.77 (t, J = 6.4 Hz 4 H, $C^{\alpha2}H_2CH_2Br$), 3.61 (broad s, 155 H, $(O(CH_2)_2)_{43}$), 3.44 (t, J = 6.4 Hz, 4 H, $C^{\alpha1}H_2Br$) ppm.
^{13}C-NMR ($CDCl_3$, 500 MHz) δ = 71.2 ($C^{\alpha2}H_2CH_2Br$), 70.6 (broad, $(O(CH_2)_2)_{43}$), 30.4 ($C^{\alpha1}H_2Br$) ppm.

Characterization of PEG2

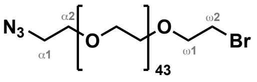

PEG2
$C_{90}H_{180}O_{44}BrN_3$
2088.31 g/mol

^{1}H-NMR ($CDCl_3$, 500 MHz) δ = 3.77 (t, J = 6.3 Hz 2 H, $C^{\omega1}H_2CH_2Br$), 3.61 (broad s, 181 H, $(O(CH_2)_2)_{43}$), 3.45 (t, J = 6.3 Hz, 2 H, $C^{\omega2}H_2Br$), 3.36 (t, J = 5.1 Hz, 2 H, $C^{\alpha1}H_2N_3$) ppm.
^{13}C-NMR ($CDCl_3$, 500 MHz) δ = 71.2 ($C^{\omega1}H_2CH_2Br$), 70.5 (broad, $(O(CH_2)_2)_{43}$), 70.1 ($C^{\alpha2}H_2CH_2N_3$), 50.7 ($C^{\alpha1}H_2N_3$), 30.4 ($C^{\omega2}H_2Br$) ppm.
FT-IR (ATR) 2101 (s, ν_s(N=N), α-terminal N_3) cm^{-1}.
m/z (Maldi-TOF) Calcd for $C_9H_{180}O_{44}BrN_3$ [M+nNa, $C_9H_{180}O_{44}BrN_3^+$]: 2111.1027; Found: 2111.1059.

Synthesis of PEGylated Imidazolium and NHC Precursors

General Procedure for the Synthesis of Imidazolium Bromides

Alkylation procedure was adapted from Jadhav *et al.* with slight modifications.[2] Under ambient conditions in a screw cap vial, brominated PEG (**PEG1** or **PEG2**, 1.000 g, 1 eq.) was dissolved in ACN (10 mL) and 1-methylimidazole (1.05 eq.) was added. The resulting solution was heated to 95 °C for 16 h, cooled to RT and concentrated under reduced pressure. Final PEGylated imidazolium bromides were obtained by addition of diethyl ether (Et_2O), followed by collection of precipitated solids *via* centrifugation and subsequent drying *in vacuo*.

Synthesis of Imidazolium Bromide 1

Reacting **PEG1** (1.000 g, 0.481 mmol) with 1-methylimidazole (0.040 mL, 0.505 mmol) yielded **1** as beige solid (0.894 g, 0.414 mmol, 86%).

^{1}H-NMR ($CDCl_3$, 500 MHz) δ = 9.92 (s, 1 H, C^2H), 7.71 (s, 1 H, C^5H), 7.44 (s, 1 H, C^4H), 4.56-4.54 (m, 2 H, $NC^{\omega2}H_2$), 4.00 (s, 3 H, NC^6H_3), 3.86-3.84 (m, 2 H, $NCH_2C^{\omega1}H_2$), 3.77-3.71 (m, 2 H, $C^{\alpha2}H_2CH_2OMe$), 3.59 (broad s, 183 H, $(O(CH_2)_2)_n$), 3.50-3.49 (m, 2 H, $C^{\alpha1}H_2OMe$), 3.32 (s, 3 H, $OC^{\alpha}H_3$) ppm.
^{13}C-NMR ($CDCl_3$, 500 MHz) δ = 137.7 (C^2H), 123.7 (C^5H), 123.0 (C^4H), 71.9 ($C^{\alpha1}H_2OMe$), 70.5 (broad, $(O(CH_2)_2)_n$), 70.4 ($C^{\alpha2}H_2CH_2OMe$), 69.1 ($NCH_2C^{\omega1}H_2$), 59.0 ($OC^{\alpha}H_3$), 49.7 ($NC^{\omega2}H_2CH_2$), 36.5 (NC^6H_3) ppm.
FT-IR (ATR) 1570 (s, ring stretch (N^1-C^2-N^3), imidazole ring) cm^{-1}.
m/z (Maldi-TOF) Calcd for $C_{95}H_{189}O_{45}N_2Br$ [M-nBr, $C_{95}H_{189}O_{45}N_2^+$]: 2079.2591; Found: 2079.2581.

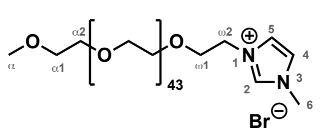

1
$C_{95}H_{189}O_{45}N_2Br$
2159.43 g/mol

Synthesis of Imidazolium Bromide 2

Reacting **PEG2** (1.000 g, 0.479 mmol) with 1-methylimidazole (0.040 mL, 0.503 mmol,) yielded **2** as beige solid (0.737 g, 0.340 mmol, 71%).

^{1}H-NMR ($CDCl_3$, 500 MHz) δ = 9.73 (s, 1 H, C^2H), 7.71 (s, 1 H, C^5H), 7.46 (s, 1 H, C^4H), 4.55-4.53 (m, 2 H, $NC^{\omega2}H_2$), 4.02 (s, 3 H, NC^6H_3), 3.88-3.86 (m, 2 H, $NCH_2C^{\omega1}H_2$), 3.78-3.72 (m, 2 H, $NCH_2C^{\alpha2}H_2$), 3.60 (broad s, 189 H, $(O(CH_2)_2)_n$), 3.47-3.43 (m, 2 H, 2 H, $C^{\alpha2}H_2CH_2N_3$), 3.35 (t, J = 5.1 Hz, 2 H, $C^{\alpha1}H_2N_3$) ppm.
^{13}C-NMR ($CDCl_3$, 500 MHz) δ = 137.5 (C^2H), 123.7 (C^5H), 123.2 (C^4H), 70.5 (broad, $(O(CH_2)_2)_n$), 70.1 ($C^{\alpha2}H_2CH_2N_3$), 69.0 ($NCH_2C^{\omega1}H_2$), 50.7 ($C^{\alpha1}H_2N_3$), 49.8 ($NC^{\omega2}H_2CH_2$), 36.7 (NC^6H_3) ppm.
FT-IR (ATR) 2101 (s, v_s(N=N), α-terminal N_3), 1570 (s, ring stretch (N^1-C^2-N^3), imidazole ring) cm^{-1}.
m/z (Maldi-TOF) Calcd for $C_{94}H_{186}O_{44}N_5Br$ [M-nBr, $C_{94}H_{186}O_{44}N_5^+$]: 2090.2498; Found: 2090.2510.

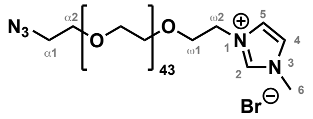

2
$C_{94}H_{186}O_{44}N_5Br$
2170.42 g/mol

General Procedure for the Synthesis of Masked NHCs

Ion metathesis procedure was adapted from Fevre *et al*. with slight modifications.[3] PEG-IMZ (0.500 g, 1 eq.) was dissolved in MeOH (abs., 5 mL), $KHCO_3$ (1.05 eq.) was added, and the obtained suspension was stirred for 48 h at 35 °C. The final mixture was cooled to 4 °C and filtered through a syringe filter (PTFE, pore size 0.4 µm). Final, masked NHCs was obtained upon removal of all volatiles under reduced pressure and used in subsequent steps without further purification.

Note: Described procedure yields a mixture of IMZ•HCO_3 and NHC-CO_2 in a H_2O dependant equilibrium (see figure S4-5).

Synthesis of Masked NHC 1'

Stirring of **1** (0.500 g, 0.232 mmol) with $KHCO_3$ (0.024 g, 0.243 mmol) yielded **1'** was obtained as beige solid (0.462 g, 0.218 mmol, 94%) *via* general procedure.

^{1}H-NMR ($CDCl_3$, 500 MHz) δ = 10.01 (s, 1 H, C^2H), 7.73 (s, 1 H, C^5H), 7.45 (s, 1 H, C^4H), 4.57-4.55 (m, 2 H, $NC^{\omega2}H_2$), 4.01 (s, 3 H, NC^6H_3), 3.86-3.84 (m, 2 H, $NCH_2C^{\omega1}H_2$), 3.76-3.71 (m, 2 H, $C^{\alpha2}H_2$), 3.59 (broad s, 187 H, $(O(CH_2)_2)_n$), 3.51-3.49 (m, 2 H, $C^{\alpha1}H_2OMe$), 3.33 (s, 3 H, $OC^{\alpha}H_3$) ppm.
^{13}C-NMR ($CDCl_3$, 500 MHz) δ = 137.6 (C^2H), 123.7 (C^5H), 123.0 (C^4H), 71.9 ($C^{\alpha1}H_2OMe$), 70.5 (broad, $(O(CH_2)_2)_n$), 70.3 ($C^{\alpha2}H_2CH_2OMe$), 69.1 ($NCH_2C^{\omega1}H_2$), 59.0 ($OC^{\alpha}H_3$), 49.7 ($NC^{\omega2}H_2CH_2$), 36.5 (NC^6H_3) ppm.
FT-IR (ATR) 1680-1620 (broad, C^2-CO_2 & $v_{as}(COO^-)$, HCO_3^-), 1570 (s, ring stretch (N^1-C^2-N^3), imidazole ring) cm^{-1}.
m/z (Maldi-TOF) Calcd for $C_{96}H_{190}O_{48}N_2$ [M-nHCO_3, $C_{95}H_{189}O_{45}N_2^+$]: 2079.2591; Found: 2079.2626.

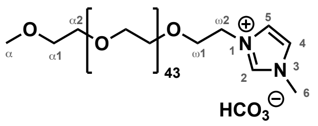

1'
$C_{96}H_{190}O_{48}N_2$
2140.54 g/mol

Synthesis of Masked NHC 2'

Stirring of **2** (0.500 g, 0.230 mmol) with $KHCO_3$ (0.024 g, 0.242 mmol) yielded **2'** was obtained as beige solid (0.403 g, 0.189 mmol, 82%) *via* procedure P2.

^{1}H-NMR ($CDCl_3$, 500 MHz) δ = 10.14 (s, 1 H, C^2H), 7.75 (s, 1 H, C^5H), 7.45 (s, 1 H, C^4H), 4.59-4.57 (m, 2 H, $NC^{\omega2}H_2$), 4.02 (s, 3 H, NC^6H_3), 3.87-3.86 (m, 2 H, $NCH_2C^{\omega1}H_2$), 3.75-3.71 (m, 2 H, $NCH_2C^{\alpha2}H_2$), 3.61 (broad s, 203 H, $(O(CH_2)_2)_n$), 3.47-3.45 (m, 2 H, 2 H, $C^{\alpha2}H_2CH_2N_3$), 3.35 (t, J = 5.2 Hz, 2 H, $C^{\alpha1}H_2N_3$) ppm.

^{13}C-NMR ($CDCl_3$, 500 MHz) δ = 137.7 (C^2H), 123.7 (C^5H), 123.0 (C^4H), 70.6 (broad, ($O(CH_2)_2)_n$), 70.4 ($C^{\alpha2}H_2CH_2N_3$), 69.1 ($NCH_2C^{\omega1}H_2$), 50.7 ($C^{\alpha1}H_2N_3$), 49.7 ($NC^{\omega2}H_2CH_2$), 36.5 (NC^6H_3) ppm.
FT-IR (ATR) 2101 (s, v_s(N=N), α-terminal N_3), 1680-1620 (broad C^2-CO_2 & $v_{as}(COO^-)$, HCO_3^-),1570 (s, ring stretch (N^1-C^2-N^3), imidazole ring) cm^{-1}.
m/z (Maldi-TOF) Calcd for $C_{99}H_{187}O_{47}N_5$ [M-nHCO$_3$, $C_{94}H_{186}O_{44}N_5^+$]: 2090.2498; Found: 2090.2494.

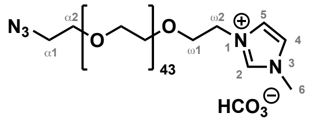

2'
$C_{95}H_{187}O_{47}N_5$
2151.53 g/mol

General Procedure for the Synthesis of PEGylated NHC-Au complexes

Transmetalation procedure was adapted Johnson and coworkers with slight modifications.[4] PEGylated imidazolium bromides (0.100 g, 1 eq.) was dissolved in DCM (abs., 2 mL) and Ag_2O (1 eq.) was added. The resulting mixture was stirred under exclusion of light at RT for 16 h and filtered through a syringe filter (PTFE, pore size 0.4 µm). DMS-Au-Cl (1.2 eq.) was added and the resulting mixture was stirred for an additional 16 h at RT. The final suspension was filtered through a syringe filter (PTFE, pore size 0.4 µm) and concentrated under reduced pressure. Addition of cold Et_2O, collection *via* centrifugation and drying *in vacuo* affords PEG-NHC-Au(I).

Note: Synthesis yields as previously described by Johnson and coworkers in a mixture of NHC-Au(I) and NHC-Au(III) complexes.[4]

Synthesis of Complex 1-Au
Transmetalation using PEG1-IMZ (0.100 g, 0.046 mmol, 1 eq.), Ag_2O (0.010 g, 0.046 mmol, 1 eq.) and DMS-Au-Cl (0.016 g, 0.056 mmol, 1.2 eq.) in DCM, yields **1-Au** (0.076 g) as pale yellow solid.

^{1}H-NMR ($CDCl_3$, 500 MHz) δ = 7.20 (d, J = 1.9 Hz, 1 H, C^5H), 6.91 (d, J = 1.9 Hz, 1 H, C^4H), 4.34-4.31 (m, 2 H, $NC^{\omega2}H_2$), 3.80 (s, 3 H, NC^6H_3), 3.77-3.74 (m, 2 H, $NCH_2C^{\omega1}H_2$), 3.62 (broad s, 269 H, ($O(CH_2)_2)_n$), 3.54-3.52 (m, 2 H, $C^{\alpha1}H_2OMe$), 3.36 (s, 3 H, $OC^{\alpha}H_3$) ppm.
^{13}C-NMR ($CDCl_3$, 500 MHz) δ = 170.7 (C^2-Au(I)), 122.6 (C^5H), 121.4 (C^4H), 71.9 ($C^{\alpha1}H_2OMe$), 70.6 (broad, ($O(CH_2)_2)_n$), 70.3 ($C^{\alpha2}H_2CH_2OMe$), 69.2 ($NCH_2C^{\omega1}H_2$), 59.1 ($OC^{\alpha}H_3$), 51.2 ($NC^{\omega2}H_2CH_2$), 38.3 (NC^6H_3) ppm.

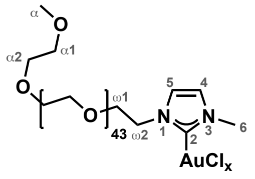

Spectra contains 2 species (NHC-Au(I) and NHC-Au(III)) with a ratio ~2:1 based on integration of C^5H imidazole-backbone signals. Reported signals correspond to NHC-Au(I) species.

1-Au
$C_{95}H_{188}O_{45}N_2AuCl$
2310.93 g/mol

Synthesis of Complex 2-Au
Transmetalation using PEG2-IMZ (0.100 g, 0.046 mmol, 1 eq.), Ag_2O (0.010 g, x mmol, 1 eq.) and DMS-Au-Cl (0.016 g, 0.055 mmol, 1.2 eq.) in DCM, yields **2-Au** (0.067 g) as pale yellow solid.

^{1}H-NMR ($CDCl_3$, 500 MHz) δ = 7.22 (d, J = 2.0 Hz, 1 H, C^2H), 6.92 (s, J = 1.9 Hz 1 H, C^5H), 7.45 (s, 1 H, C^4H), 4.35-4.33 (m, 2 H, $NC^{\omega2}H_2$), 3.82 (s, 3 H, NC^6H_3), 3.80-3.76 (m, 4 H, $NCH_2C^{\omega1}H_2$ & $NCH_2C^{\alpha2}H_2$), 3.64 (broad s, 215 H, ($O(CH_2)_2)_n$), 3.50-3.45 (m, 2 H, $C^{\alpha2}H_2CH_2N_3$), 3.38 (t, J = 5.1 Hz, 2 H, $C^{\alpha1}H_2N_3$) ppm.
^{13}C-NMR ($CDCl_3$, 500 MHz) δ = 170.8 (C^2-Au(I)), 122.6 (C^5H), 121.5 (C^4H), 70.6 (broad, ($O(CH_2)_2)_n$), 70.5 ($C^{\alpha2}H_2CH_2N_3$), 69.2 ($NCH_2C^{\omega1}H_2$), 51.2 ($C^{\alpha1}H_2N_3$), 50.8 ($NC^{\omega2}H_2CH_2$), 38.3 (NC^6H_3) ppm.

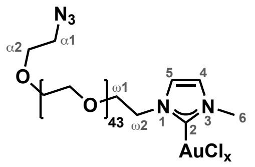

Spectra contains 2 species (NHC-Au(I) and NHC-Au(III)) with a ratio ~3:2 based on integration of C^5H imidazole-backbone signals. Reported signals correspond to NHC-Au(I) species.

2-Au
$C_{94}H_{185}O_{44}N_5AuCl$
2321.92 g/mol

Synthesis of Oleylamine stabilized AuNPs

The synthesis protocol for OAm@AuNPs was adapted from Peng *et al*. with slight modifications.[5] Under N_2 atmosphere, $HAuCl_4\cdot3H_2O$ (0.025 g, 0.063 mmol, 1 eq.) was dissolved in tetralin/OAm (1:1, 5 mL) followed by the addition of tertbutyl amino borane complex (tBuNH$_2\cdot$BH$_3$, 0.011 g, 0.126 mmol, 2 eq.) in tetralin/OAm (1:1, 1 mL) as a single shot. The resulting mixture was stirred for 1 h at RT. The red dispersion was added to MeOH (40 mL) and centrifuged (7500 rpm, 5 min). The collected

dark principate was resuspended in acetone and collected by centrifugation (7500 rpm, 5 min). The final dark solid (0.032 g) was dried *in vacuo*.

FT-IR (ATR) 3311 (w, ν_s(N-H), primary $-NH_2$), 2922 (s, ν_{as}(C-H), $-CH_2-$), 2856 (s, ν_s(C-H), $-CH_2-$) cm^{-1}.
UV-Vis (THF, λ_{max}) 515 nm.
TEM (d, n=100): 4.7 ± 0.8 nm.

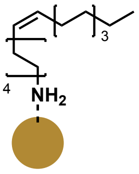

OAm@AuNP

Synthesis of PEG-NHC@AuNPs *via* Top-down Approach

Employed NHC amount in top-down procedures **P1**-**P4** equals 10 equivalents OAm content of used **OAm@AuNPs.** OAm content based on TGA 22 wt%.

All crude solid PEG-NHC@AuNPs obtained by the following TD exchange approaches were transferred into dialysis tubing (cellulose, MWCO 13.5k) and dialyzed against H_2O (24 h, 5 × H_2O exchange). Solid PEG-NHC@AuNPs were obtained by lyophilization or drying *in vacuo*.

Synthesis of PEG-NHC@AuNP *via* Procedure P1

Imidazolium bromide **1** (0.032 g, 1 eq.) was dissolved in THF (abs., 0.5 mL) followed by the addition of solid KHMDS (0.003 g, 1 eq.). The mixture was stirred at RT for 1 h. The resulting mixture was filtered through a syringe filter (PTFE, pore size 0.4 µm) into **OAm@AuNPs** (0.72 mL, ~2.5 mg/mL in abs. THF) and stirred at 40 °C for 62 h. Dialysis yielded **AuNP-1** with partial damage of the PEG chain.

^{1}H-NMR (D_2O, 500 MHz) δ = 7.37 (s, C^5H), 7.29 (s, C^4H), 4.48-4.46 (m, $NC^{\omega 2}H_2$), 4.02-4.00 (m, $NCH_2C^{\omega 1}H_2$), 3.93 (s, NC^6H_3), 3.87-3.84 (m, $CH_2C^{\alpha 1}H_2OMe$), 3.72 (broad s, $(O(CH_2)_2)_n$), 3.58 (m, $C^{\alpha 2}H_2CH_2OMe$), 3.40 (s, $OC^{\alpha}H_3$) ppm.
^{13}C-NMR (D_2O, 500 MHz) δ = 184.0 (C^2-AuNP), 123.1 (C^4H), 122.0 (C^5H), 70.2 ($NCH_2C^{\omega 1}H_2$) 69.7 (broad, $(O(CH_2)_2)_n$), 58.3 ($OC^{\alpha}H_3$), 50.4 ($NC^{\omega 2}H_2CH_2$), 37.7 (NC^6H_3) ppm.
FT-IR (ATR): 1680-1620 (broad, $\nu_{as}(COO^-)$, HCO_3^- surface bond; ν_s(C=C), α-terminal vinyl),), 1570 (w, ring stretch (N^1-C^2-N^3), imidazole ring) cm^{-1}.

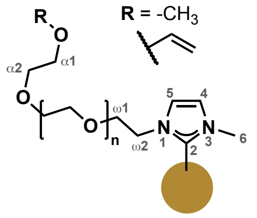

AuNP-1

Spectra contains secondary species with α-terminal vinyl group instead of α-MeO (ratio α-MeO/vinyl ~3:1, ^{13}C-NMR data extracted from ^{1}H/^{13}C HMBC).

^{1}H-NMR$_{\alpha\text{-}vinyl}$ (D_2O, 500 MHz) δ = 6.53 (dd, J = 14.3, 6.8 Hz, 1 H, $CH_2CH{=}CH_2$), 4.36 (dd, J = 6.7, 2.2 Hz, 1 H, $C^{\alpha 1}H_2CH{=}CH^a_2$), 4.17 (dd, J = 14.3, 2.3 Hz, 1 H, $C^{\alpha 1}H_2CH{=}CH^b_2$), 3.94 (m, 2 H, $C^{\alpha 1}H_2OCH{=}CH_2$) ppm.
^{13}C-NMR$_{\alpha\text{-}vinyl}$ (D_2O, 500 MHz) δ = 151.1 ($C^{\alpha 1}H_2CH{=}CH_2$), 88.1 ($C^{\alpha 1}H_2CH{=}CH_2$), 67.3 ($C^{\alpha 1}H_2CH{=}CH_2$) ppm.

Synthesis of PEG-NHC@AuNPs *via* Procedure P2

NHC Precursor **1'** (0.032 g) was suspended in THF (abs., 0.5 mL) and **OAm@AuNPs** (0.72 mL, ~2.5 mg/mL in abs. THF) was added. The resulting mixture was stirred at RT for 62 h. Obtained particles were dried under reduced pressure, redispersed in H_2O (5 mL) and washed with hexanes (2 × 5 mL). The aqueous phase was dialysed, yielding **AuNP-2** as aqueous dispersion. **AuNP-2** were dried *in vacuo* transferred to the glovebox and redispersed in THF. Subsequent, heating to 40 °C for 62 h (procedure **P2**$_{\Delta}$) yielded **AuNP-3** without further purification.

Characterization of AuNP-2

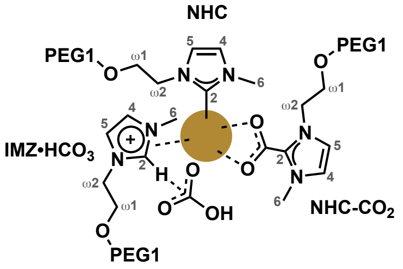

AuNP-2

^{1}H-NMR (D_2O, 500 MHz) δ = 7.54 (IMZ•HCO_3, d, J = 2.0 Hz, C^5H), 7.51 (NHC-CO_2, d, J = 1.7 Hz, C^5H), 7.46 (IMZ•HCO_3, d, J = 2.3 Hz, C^4H), 7.42 (NHC-CO_2, d, J = 2.3 Hz, C^4H), 7.36 (NHC, d, J = 1.7 Hz, C^5H), 7.29 (NHC, d, J = 1.7 Hz, C^4H), 4.46 (NHC, t, J = 5.15 Hz, $NC^{\omega 2}H_2$), 4.42-4.40 (IMZ•HCO_3, m, $NC^{\omega 2}H_2$), 4.01-3.99 (NHC& IMZ•HCO_3, m, $NCH_2C^{\omega 1}H_2$), 3.92 (NHC, s, NC^6H_3), 3.91 (IMZ•HCO_3, s, NC^6H_3) ppm.

^{13}C-NMR (D_2O, 500 MHz) δ = 184.0 (NHC, C^2-AuNP), 141.7* (NHC-CO_2, C^2-CO_2), 137.0* (IMZ•HCO_3, C^2H), 123.5 (IMZ•HCO_3, C^4H), 123.1 (NHC, C^4H), 122.6 (IMZ•HCO_3, C^5H), 122.0 (NHC, C^5H), 50.3 (NHC, $NC^{\omega 2}H_2CH_2$), 49.1 (IMZ•HCO_3, $NC^{\omega 2}H_2CH_2$), 37.5 (NHC, NC^6H_3), 35.7 (IMZ•HCO_3, NC^6H_3) ppm.

Spectra contains 3 species including NHC/IMZ•HCO_3/NHC-CO_2 attached to the AuNP with a ratio of ~6:3:1 based on integration of C^5H imidazole-backbone signals. *Signals extracted from ^{1}H/^{13}C-HMBC spectra.

FT-IR (ATR) 1680-1620 (broad, C^2-CO_2 & $\nu_{as}(COO^-)$, HCO_3^- surface bond), 1570 (w, ring stretch (N^1-C^2-N^3), imidazole ring) cm^{-1}.
UV-Vis (H_2O, λ_{max}) 578 nm.
TEM (d, n=100) 5.1 ± 1.3 nm.

General Procedure for the Synthesis of AuNP-3 (P3)

The corresponding masked NHC (0.032 g) was suspended in THF (abs., 0.5 mL), and **OAm@AuNP**s (0.72 mL, ~2.5 mg/mL in abs. THF) was added. The resulting mixture was heated to 40 °C for 62 h, cooled to RT and all volatiles were removed under reduced pressure. The obtained dark purple solid was redispersed in H_2O (5 mL) and washed with hexanes (2 × 5 mL). The aqueous phase was dialysed, yielding PEG-NHC@AuNPs as aqueous dispersion.

Synthesis of AuNP-3
Upon purification, **AuNP-3** was obtained as red aq. dispersion or dark purple solid *via* procedure **P3**.

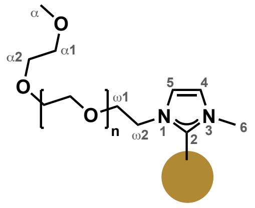
AuNP-3

^{1}H-NMR (D_2O, 500 MHz) δ = 7.37 (s, C^5H), 7.30 (s, C^4H), 4.48-4.46 (m, $NC^{\omega 2}H_2$), 4.02-4.00 (m, $NCH_2C^{\omega 1}H_2$), 3.93 (s, NC^6H_3), 3.87-3.84 (m, $C^{\alpha 2}H_2$), 3.72 (broad s, $(O(CH_2)_2)_n$), 3.58 (broad, $C^{\alpha 1}H_2OMe$), 3.40 (s, $OC^{\alpha}H_3$) ppm.
^{13}C-NMR (D_2O, 500 MHz) δ = 184.0 (C^2-AuNP), 123.1 (C^4H), 122.0 (C^5H), 71.0 ($C^{\alpha 2}H_2CH_2OMe$), 69.64 (broad s, 182 H, $(O(CH_2)_2)_n$), 69.5 ($NCH_2C^{\omega 1}H_2$), 58.1 ($OC^{\alpha}H_3$), 50.2 ($NC^{\omega 2}H_2CH_2$), 37.5 (NC^6H_3)ppm.
FT-IR (ATR) 1680-1620 (broad, $\nu_{as}(COO^-)$, HCO_3^- surface bond), 1570 (w, ring stretch (N^1-C^2-N^3), imidazole ring) cm^{-1}.
UV-Vis (H_2O, λ_{max}) 515 nm.
TEM (d, n=100) 4.6 ± 0.7 nm.

Synthesis of Azide-containing AuNP-4
Upon purification, **AuNP-4** was obtained as red aq. dispersion or dark purple solid *via* procedure **P3**.

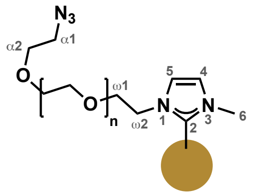

AuNP-4

^{1}H-NMR (D_2O, 500 MHz) δ = 7.35 (d, J = 2.2 Hz, C^5H), 7.28 (d, J = 2.0 Hz, C^4H), 4.46-4.44 (m, $NC^{\omega 2}H_2$), 4.00-3.98 (m, $NCH_2C^{\omega 1}H_2$), 3.91 (s, NC^6H_3), 3.86-3.83 (m, $CH_2C^{\alpha 1}H_2N_3$), 3.71 (broad s, $(O(CH_2)_2)_n$), 3.57-3.55 (m, $C^{\alpha 2}H_2CH_2N_3$), 3.51 (t, J = 4.9 Hz, $C^{\alpha 1}H_2N_3$) ppm.
^{13}C-NMR (D_2O, 500 MHz) δ = 184.0 (C^2- AuNP), 123.1 (C^4H), 122.0 (C^5H), 71.8 ($C^{\alpha 2}H_2CH_2N_3$), 69.64 (broad, $(O(CH_2)_2)_n$), 60.4 ($NCH_2C^{\omega 1}H_2$), 50.2 ($C^{\alpha 1}H_2N_3$ & $NC^{\omega 2}H_2CH_2$), 37.5 (NC^6H_3) ppm.
FT-IR (ATR) 2101 (s, ν_s(N=N), α-terminal N_3), 1680-1620 (broad C^2-CO_2 & $\nu_{as}(COO^-)$, HCO_3^- surface bond) cm^{-1}.
UV-Vis (H_2O, λ_{max}) 515 nm.
TEM (d, n=100) 4.6 ± 0.7 nm.

Synthesis of PEG-NHC@AuNPs *via* One-pot Approach (P4)

Imidazolium salt **1** (0.032 g, 1 eq.) was dissolved in THF (abs., 0.5 mL), $KHCO_3$ (0.004 g, 3 eq.) and **OAm@AuNPs** (0.72 mL, ~2.5 mg/mL in abs. THF) were added. The resulting mixture was heated to 40 °C for 62 h, worked up according to procedure P3 and yielded **AuNP-3** as aq. dispersion.

General Procedure for the Synthesis of PEG-NHC@AuNP *via* Bottom-up Approach (P5)

PEG-NHC-Au complexes (0.020 g, 1 eq.) were dissolved in THF (1.5 mL) and vigorously stirred at RT. *t*$BuNH_2$•BH_3 (2 eq.) was dissolved in THF (0.5 mL) and added as a single shot to the PEG-NHC-Au(I) solution. The resulting clear solution was stirred at RT for 16 h and was quenched by the addition of H_2O (1 mL) resulting a brown dispersion. Removal of all THF under reduced pressure, dilution in additional H_2O (9 mL) and subsequent dialysis (cellulose, MWCO 13.5k, 24 h, 5 × H_2O exchange) yields the final PEG-NHC@AuNP as a red dispersion. Solid PEG-NHC@AuNPs were obtained by lyophilization or drying *in vacuo*.

Note: PEG-NHC@AuNPs obtained *via* the explained bottom-up approach show significant ripening during applied workup procedures (e.g. concentration and/or drying under reduced pressure). Experimental data below was collected from AuNPs after full workup.

Synthesis of AuNP-3^{BU}

Upon purification, **AuNP-3^{BU}** was obtained as red aq. dispersion or dark purple solid *via* procedure **P5**.

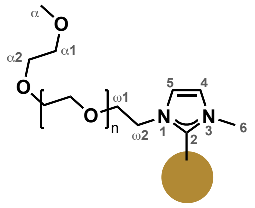

AuNP-3^{BU}

^{1}H-NMR (D_2O, 500 MHz) δ = 7.34 (d, J = 1.8 Hz, C^5H), 7.26 (d, J = 1.8 Hz, C^4H), 4.45 (t, J = 5.1 Hz, $NC^{\omega 2}H_2$), 3.97 (t, J = 5.1 Hz, $NCH_2C^{\omega 1}H_2$), 3.89 (s, NC^6H_3), 3.87-3.80 (m, $C^{\alpha 2}H_2$), 3.69 (broad s, $(O(CH_2)_2)_n$), 3.52-3.50 (broad, $C^{\alpha 1}H_2OMe$), 3.37 (s, $OC^{\alpha}H_3$) ppm.
UV-Vis (H_2O, λ_{max}) 510 nm.
TEM (d, n=100) 3.2 ± 1.6 nm.

Synthesis of Azide-containing AuNP-4^{BU}

Upon purification, **AuNP-4^{BU}** was obtained as red aq. dispersion or dark purple solid *via* procedure **P5**.

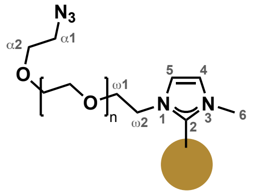

AuNP-4^{BU}

^{1}H-NMR (D_2O, 500 MHz) δ = 7.34 (s, C^5H), 7.26 (s, C^4H), 4.44 (s, $NC^{\omega 2}H_2$), 3.98 (s, $NCH_2C^{\omega 1}H_2$), 3.90 (s, NC^6H_3), 3.86 (s, $CH_2C^{\alpha 1}H_2N_3$), 3.69 (broad s, $(O(CH_2)_2)_n$), 3.60 (s, $C^{\alpha 2}H_2CH_2N_3$), 3.50 (s, $C^{\alpha 1}H_2N_3$) ppm.
UV-Vis (H_2O, λ_{max}) 508 nm.
TEM (d, n=100) 3.2 ± 2.0 nm.

Setup of Stability Studies

Used conditions were adapted form Johnson and coworkers.[4] Dry PEG-NHC@AuNPs were dissolved in DI H_2O and kept as stock (~1 mg/ml) at RT. AuNP stock (0.15 mL) were mixed with analytes (0.15 mL) and vortexed (1 min) giving a final mixture with desired analyte concentration and AuNP concentration of ~0.5 mg/mL. UV-Vis absorbance spectra of aliquots (0.03 mL) kept at RT were measured at certain time intervals (0, 1, 2, 4, 6, 24, 48 h and 1 week). Used analyte stocks 10xPBS, HCl at pH 2, NaOH at pH 12, NaCl (1.2 M), GHS (6 mM) and PEG-SH (M_n = 2000 g/mol, 6 mM).

For FBS and H_2O_2 stability tests, PEG-NHC@AuNPs (0.15 mL, ~1 mg/mL in H_2O) were centrifuged (14.5k rpm, 30 min) and the supernatant was discarded. The obtained pellet was redispersed in 0.3 mL FBS or H_2O_2 (~1 M), respectively. UV-Vis absorbance spectra of aliquots (0.03 mL) kept at RT were recorded at certain time intervals (0, 1, 2, 4, and 6 h).

Conjugation of AuNPs *via* Click Chemistry

CuAAC Procedure

CuAAC experiments were carried out in NMR tubes under ambient conditions. Experiments were conducted in D_2O/MeOD 32:1 and a molar ratio of N_3/alkyne 1:1.

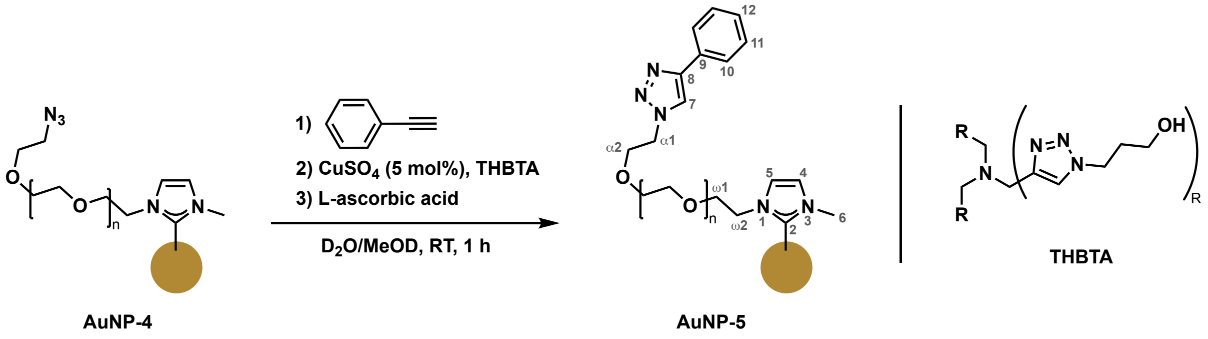

Figure S1 Reaction scheme of CuAAC of **AuNP-4** with phenylacetylene.

For CuAAC reactions, THBTA (2 mg/mL, 50 µL in D_2O) and $CuSO_4 \cdot 5H_2O$ (5 mg/mL, 6 µL in D_2O) were premixed in a HPLC vial. The resulting mixture was vortexed for 1 min and left undisturbed under exclusion of light for 30 mins. The TBTA/$CuSO_4$ mixture was prepared with a target Cu loading of 5 mol%. **AuNP-4** (N_3 content 0.002 mmol, 0.005 g in 508 µL D_2O) and phenylacetylene (10 mg/mL, 20 µL in MeOH) were mixed in an NMR tube. The premixed TBTA/$CuSO_4$ solution was added to the NMR tube, followed by L-ascorbic acid (5 mg/mL 16 µL in D_2O). Reaction kinetics were monitored by ^{1}H-NMR for 8 h.

^{1}H-NMR (D_2O, 500 MHz) δ = 8.00 (s, C^7H), 7.53 (broad s, $C^{10}H$), 7.39 (broad s, $C^{11/12}H$), 7.34 (d, J = 1.9 Hz, C^5H), 7.24 (d, J = 1.9 Hz, C^4H), 4.50 (t, J = 7.0 Hz, $C^{\alpha1}H_2N$), 4.43 (t, J = 5.3 Hz, $NC^{\omega2}H_2$), 4.02-4.00 (m, $C^{\alpha2}H_2CH2N$), 3.97 (t, J = 5.2 Hz, $NCH_2C^{\omega1}H_2$), 3.89 (s, C^6H_3), 3.84-3.48 (remaining PEG chain signals) ppm.

SPAAC Procedure

SPAAC experiments were carried out under ambient conditions. Experiments were conducted in a mix of H_2O/EtOH (1:1) and a molar ratio of N_3/DBCO 2:1.

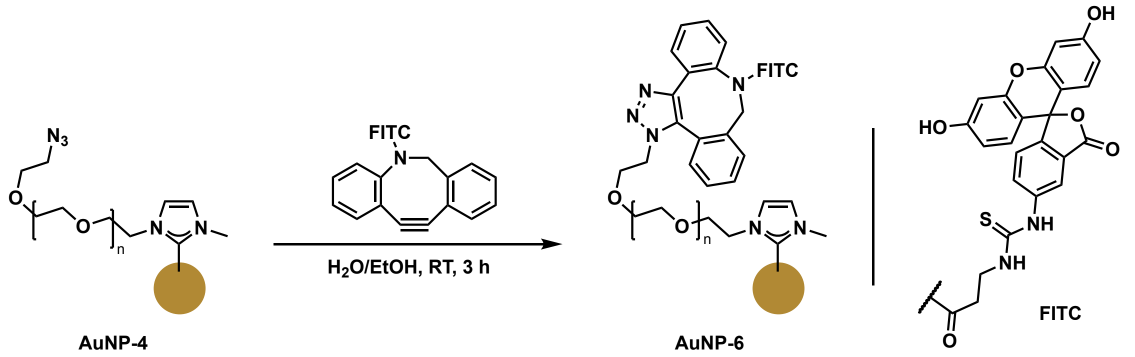

Figure S2 Reaction scheme of SPAAC of **AuNP-4** with DBCO-FITC.

AuNP-4 (150 µL, 0.5 mg/mL in EtOH/H_2O 1:1) were added to a well and mixed with DBCO-FITC (22.5 µL, 0.5 mg/mL in H_2O/EtOH 1:1). 30 µL of the reaction mixture was immediately transferred into a well of a 384 well plate and the reaction process at RT was tracked *via* the DBCO specific band at 310 nm over 3 h *via* UV-vis.The remaining solution was kept at RT under the exclusion of light for an identical time period (3 h). 100 µL of the final bright yellow reaction mixture was transferred into an Amicon Ultra centrifugal filter (MWCO 3k) and concentrated to 20 µL *via* centrifugation (14k rpm, 1 h). The concentrate was washed with additional H_2O/EtOH (1:1) and concentrated with prior described conditions. The final concentrate was diluted in H_2O/EtOH (100 µL, 1:1) and used without further purification.

Additional Figures

General Reaction Scheme of Imidazolium Bromide Synthesis

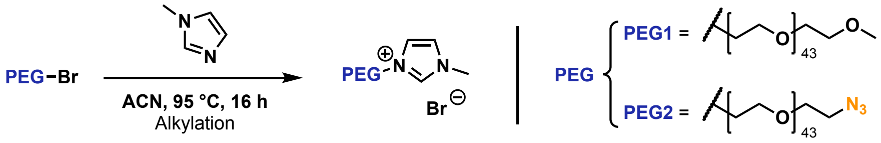

Figure S3 Alkylation procedure of 1-methylimidazol using brominated PEG chains.

^{1}H-NMR Comparison of Masked NHC 1' in Different Solvents

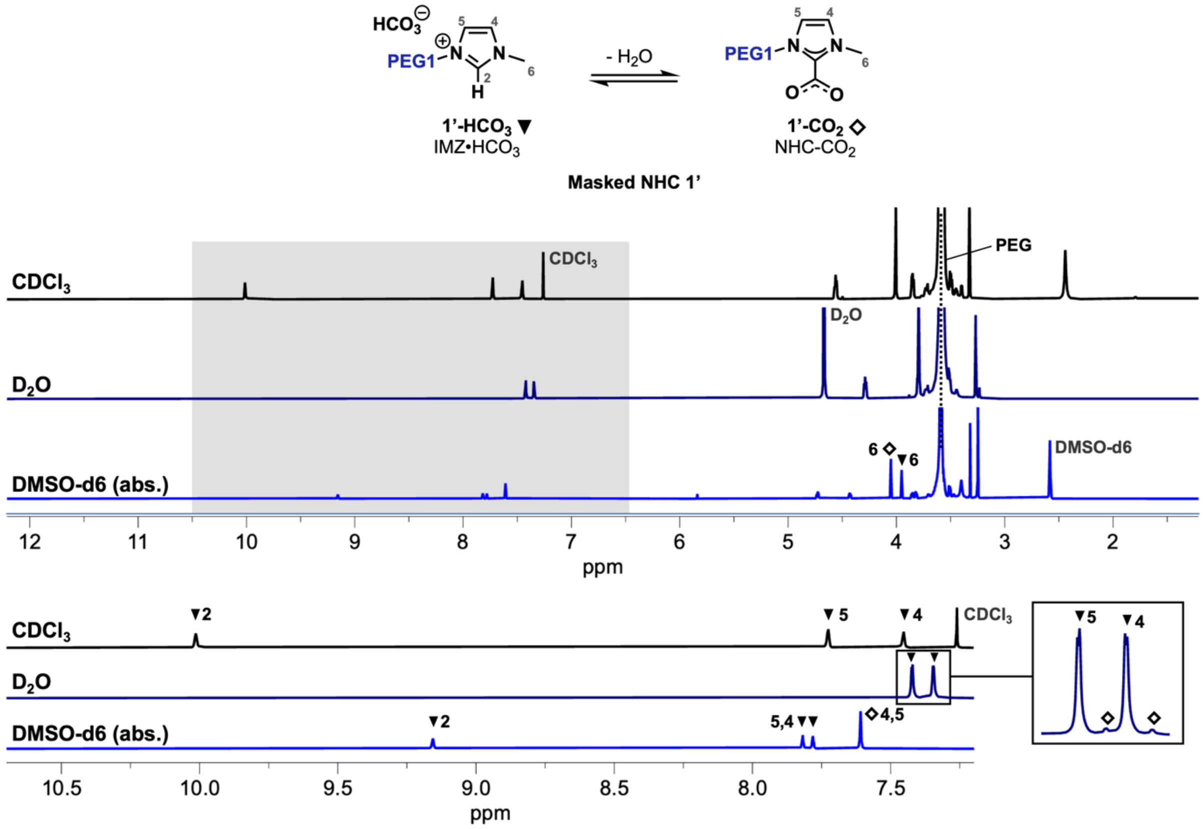

Figure S4 ^{1}H-NMR (500 MHz) comparison of **1'** in different deuterated solvents. For comparison reasons, all spectra are referenced to the PEG chain peak position (3.59 ppm) from the d_6-DMSO spectrum.

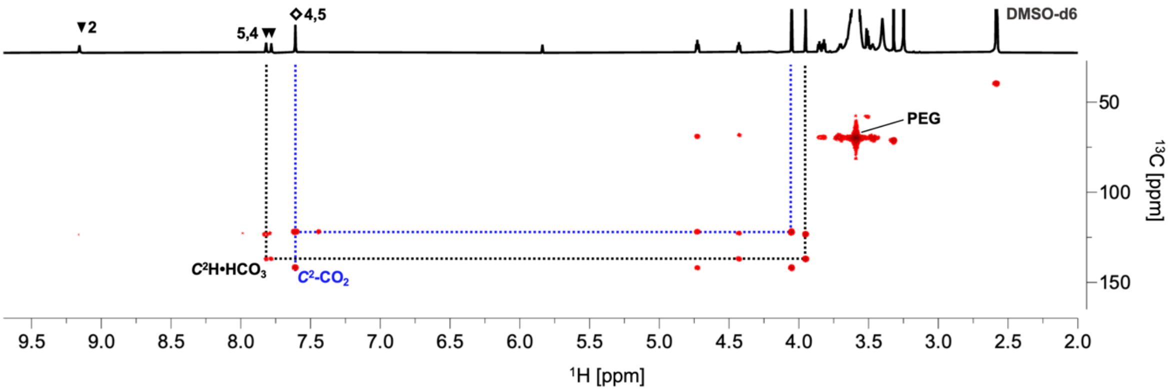

Figure S5 $^1H/^{13}C$-HMBC (500 MHz, d_6-DMSO) of **1'**.

Recorded ^1H-NMR spectra highlight the equilibrium of the masked NHC **1'** based on the presence of water. The initially recorded spectra in non-absolute $CDCl_3$ shows only the **1'-HCO$_3$** with a low field shift of the C^2H proton indicating successful ion metathesis from Br^- to HCO_3^-. The residual water content in $CDCl_3$ is sufficient to shift the equilibrium solely to **1'-HCO$_3$** (IMZ•HCO_3).[3] The same equilibrium shift is observed using D_2O as solvent. However, rapid H/D exchange at the C^2H position this signal can no longer be observed. Despite the protic conditions in D_2O, traces of **1'-CO$_2$** (NHC-CO_2) remain detectable. Finally, **1'** was measured in dry aprotic d_6-DMSO revealing the ratio of NHC-CO_2 adduct and IMZ•HCO_3 as ~3:2 (based on integration of the respective -C^6H_3 signals). Further, ^1H-/^{13}C-HMBC spectra reveal the presence of characteristic peaks of C^2-CO_2 and C^2H•HCO_3 at 141.7 and 137.0 ppm, respectively.[3]

FT-IR Comparison of Precursors and AuNPs Obtained by TD Procedures

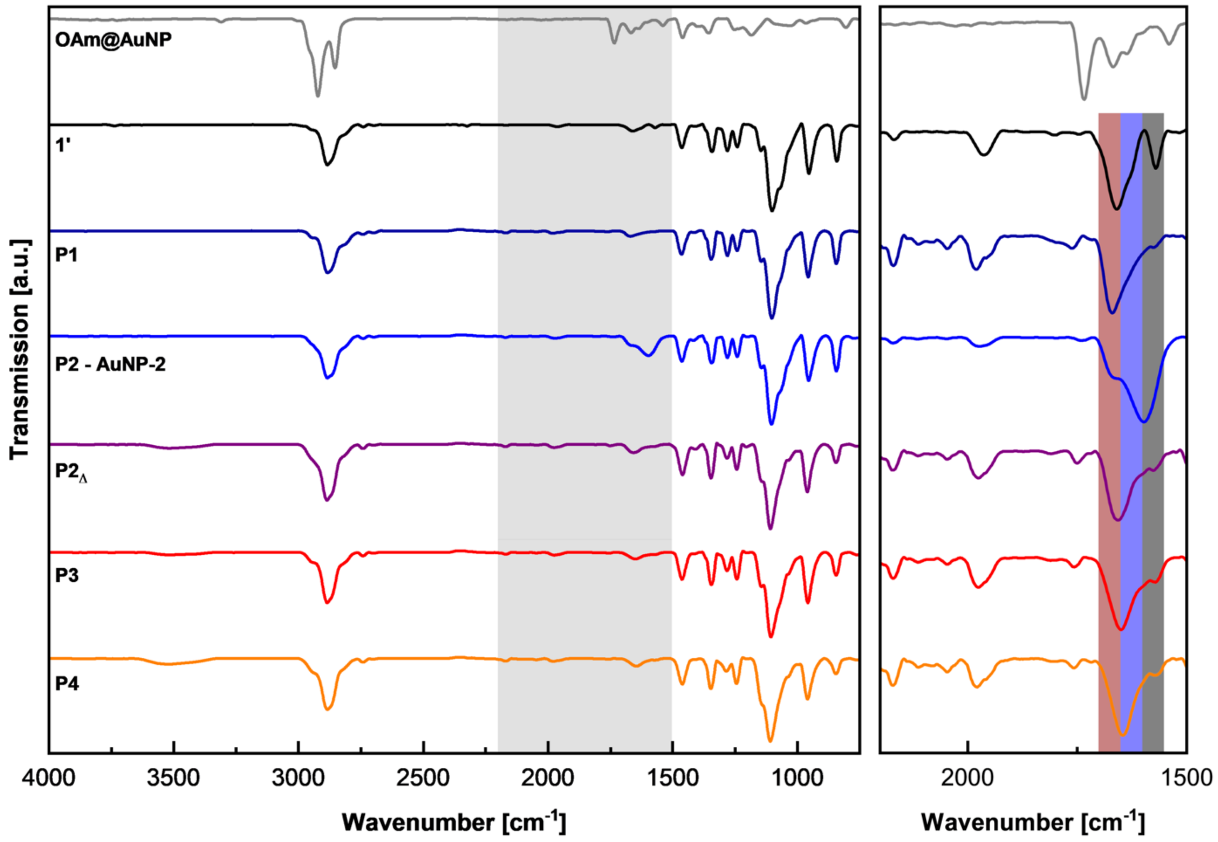

Figure S6 FT-IR spectra of **Aunp-3** obtained by procedures **P1-P4** and their precursors **1'** and **OAm@AuNP**s.

Installation of PEG-NHCs on the surface of **OAm@AuNP**s are indicated by the disappearance or significant lowering in intensity of the ring stretch (N^1-C^2-N^3,1570 cm^{-1}) upon coordination (Figure S5).[4, 6] The retention of the HCO_3^- band (■) at ~ 1650 cm^{-1} is associated with HCO_3^- and related species coordinated to the AuNP surface (**P1-P4**).[7] **AuNP-2** obtained by **P2** show still a

significant contribution of surface bound NHC-CO_2 (■) and IMZ•HCO_3 (■). Furthermore, full ligand exchange is indicated by the absence of any OAm related signals (3311, 2922, 2856 and 1750-1500 cm^{-1}) in the spectra of final PEG-NHC@AuNPs.

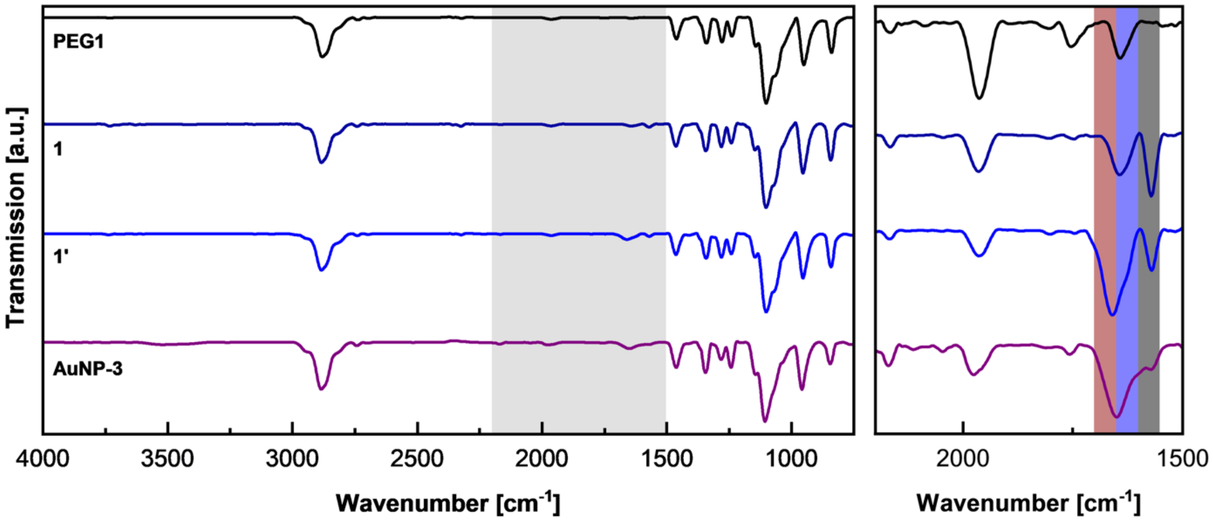

Figure S7 FT-IR Spectra of **PEG1** containing molecular compounds and AuNPs.

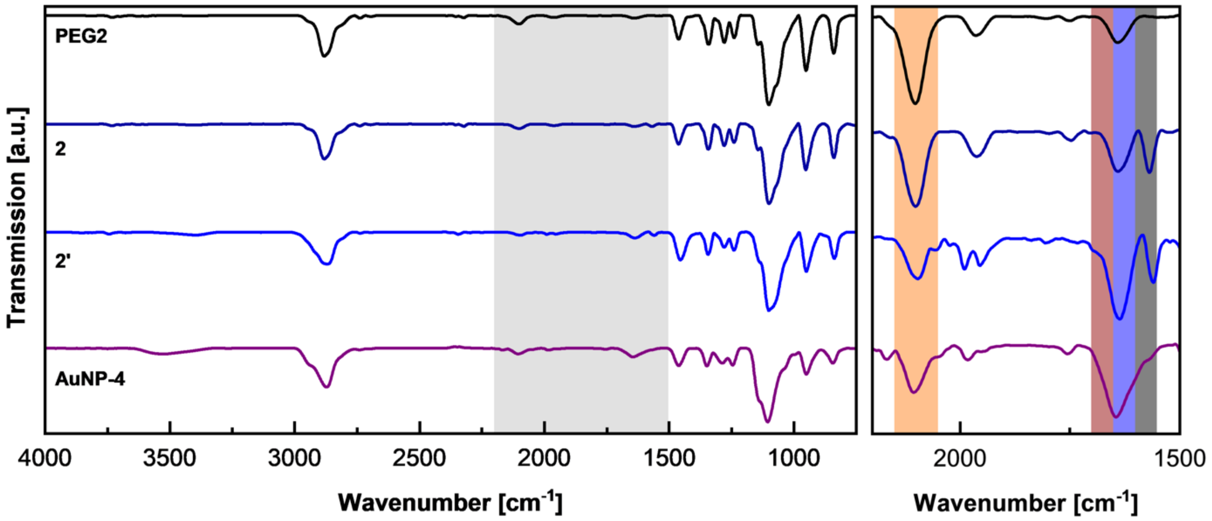

Figure S8 FT-IR Spectra of **PEG2** containing molecular compounds and AuNPs.

FT-IR spectra comparison of compounds including **PEG1** and **PEG2** (Figure S2-3), show the successful alkylation step, with the appearance of the imidazolium ring stretch (N^1-C^2-N^3, ■) at 1570 cm^{-1}.[4, 6] Successful ion metathesis is indicated by the appearance of a broad band at ~1650 cm^{-1} associated with the formation of NHC-CO_2 adduct (1600-1625 cm^{-1}, ■)[8] and the presence of the HCO_3^- counterion as $v_{as}(COO^-$, ■) in the region of 1680-1640 cm^{-1}. In case of **PEG2**-containing compounds and AuNPs, the band at 2101 cm^{-1} v_s(N=N) characteristic for terminal N_3 (■) moieties is retained throughout all synthesis steps.

^{1}H-NMR Comparison of Imidazolium Precursor and AuNPs Obtained by TD Procedures

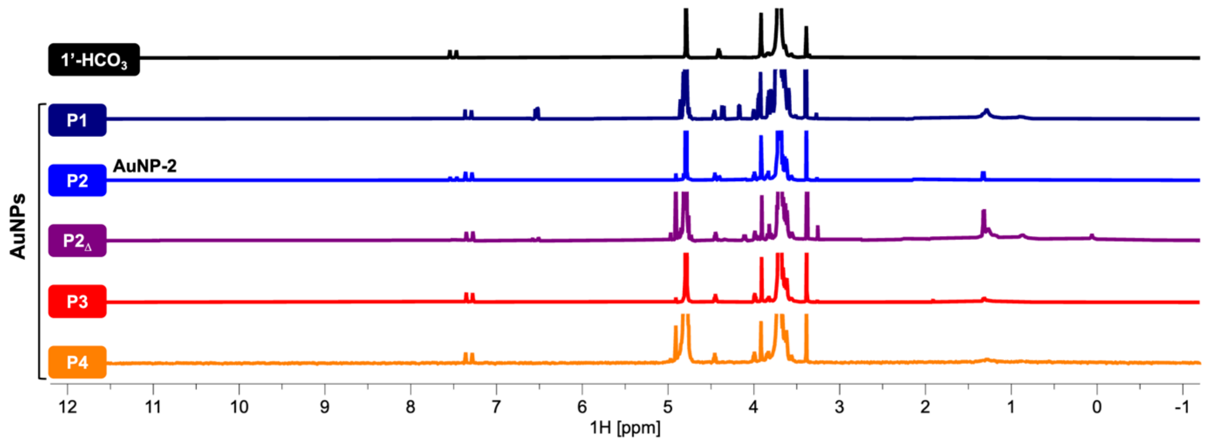

Figure S9 Full ^{1}H-NMR spectra of **1'** and AuNPs obtained by procedures **P1-P4**.

XPS Comparison of Precursors and AuNPs obtained by TD Procedures

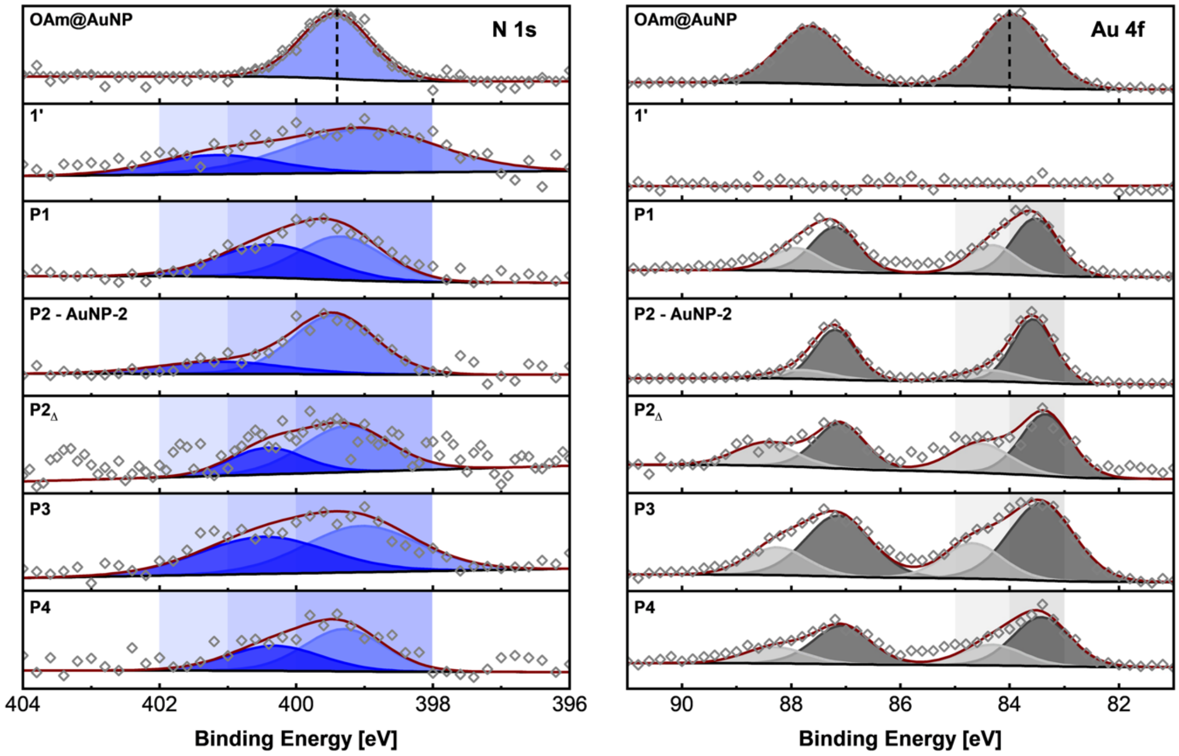

Figure S10 Left: Comparison of N 1s XPS spectra - quaternary N / IMZ / NHC / -NH_2 lit. **---** (399.4 eV); Right: Comparison of Au 4f XPS spectra - Au(0) / Au(I) / Au(0) lit. **---** (84 eV).

Comparing N 1s and Au 4f XPS traces of **AuNP-3** obtained by different procedures as well as their precursors, visualizes the presences of the imidazole ring structure in different chemical states. Ligand precursor **1'** as measured shows an IMZ contribution (402-401 eV,) due to exposure of the sample to ambient humidity during transfer to XPS machine. Procedures **P1-P4** show the conversion of the IMZ structure into the surface bound NHC structure (401-400 eV,). **AuNP-2** obtained by **P2** show not only the conversion from IMZ to a NHC structure but also the increase of detectable Au(I) () associated with the successful coordination of NHCs.[9]

Comparison of UV-Vis Spectra of AuNPs Obtained by Procedures P1-P4

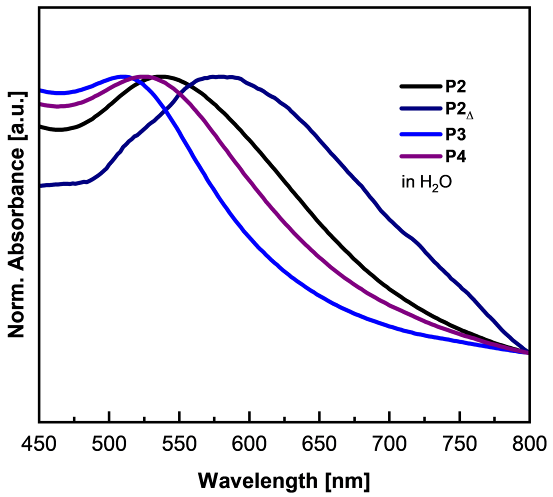

Figure S11 Comparison of UV-Vis absorbance spectra of **AuNP-3** obtained by procedures **P2**-**P4**.

Comparison of ^{1}H-NMR Spectra of OAm@AuNP and AuNPs from TD and BU Procedures

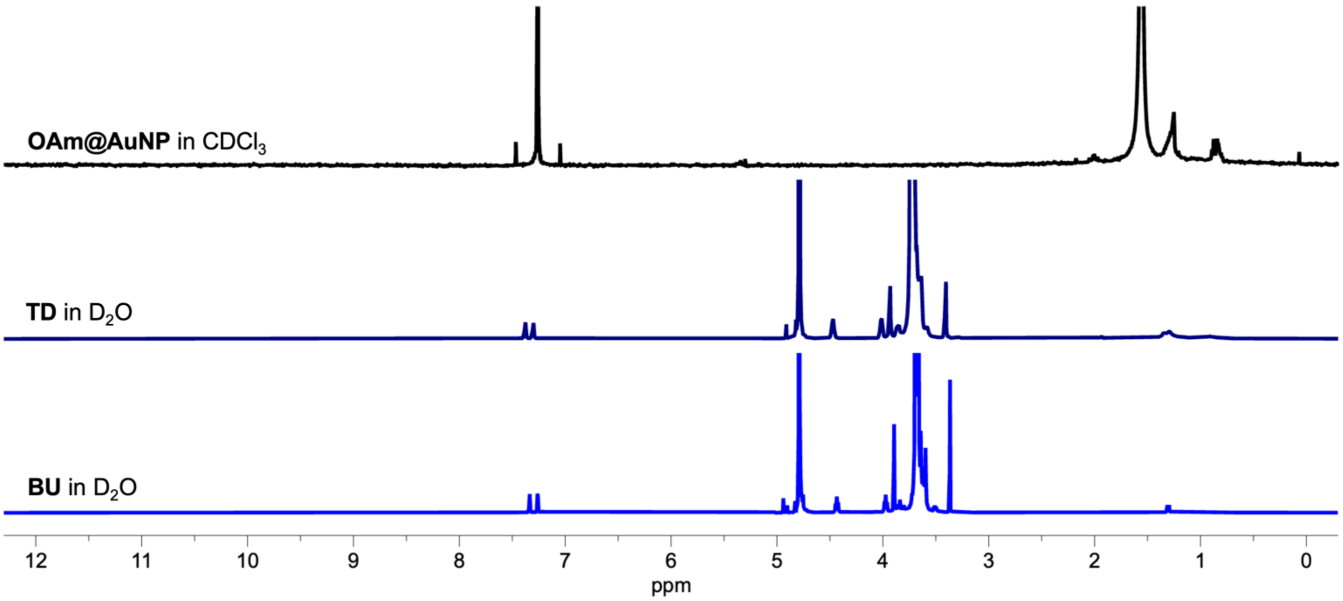

Figure S12 Comparison of ^{1}H-NMR (500 MHz) of **OAm@AuNP**, **AuNP-3** and **AuNP-3^{BU}**.

High Resolution XPS Spectra

All displayed spectra were deconvoluted using the Avantage software package provided by Thermo Fisher. Displayed baselines were generated by using the Shirley baseline. Raw data is displayed as gray symbol, generated envelope as red line and peak fits as coloured areas. All high resolution XPS spectra were calibrated on the C-C contribution in C 1s spectra at 284.8 eV.

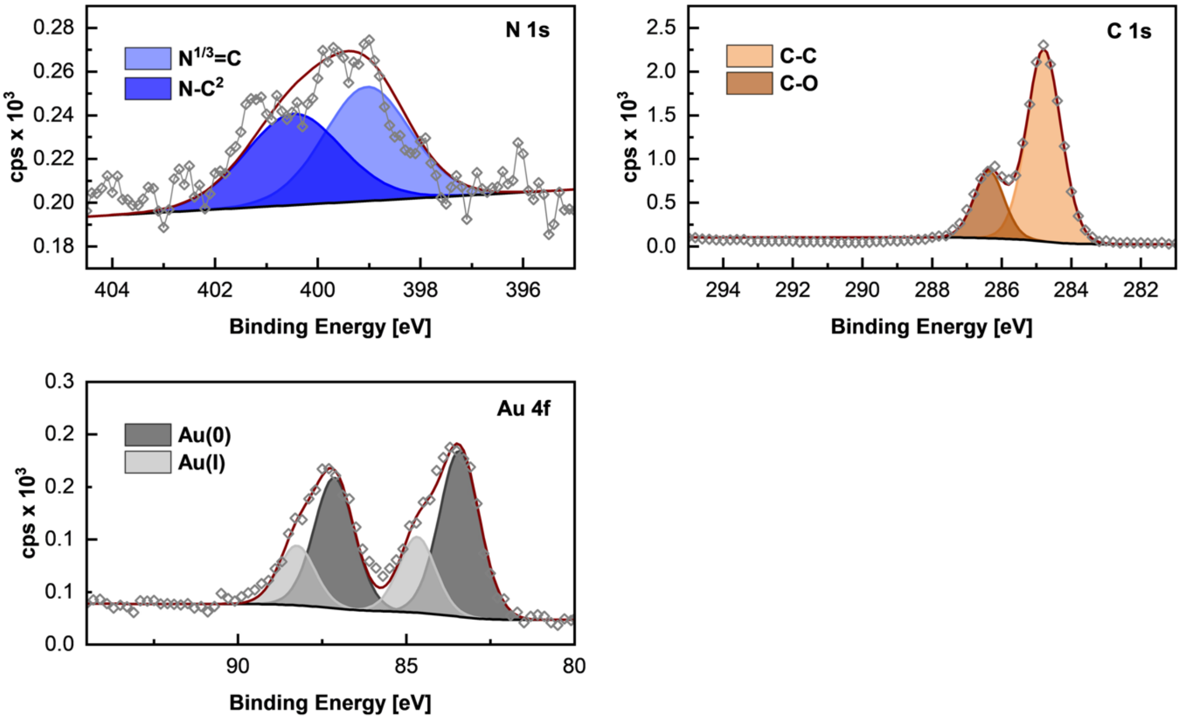

Figure S13 High resolution XPS spectra of N 1s, C 1s and Au 4f regions of **AuNP-3** obtained by procedure **P3**.

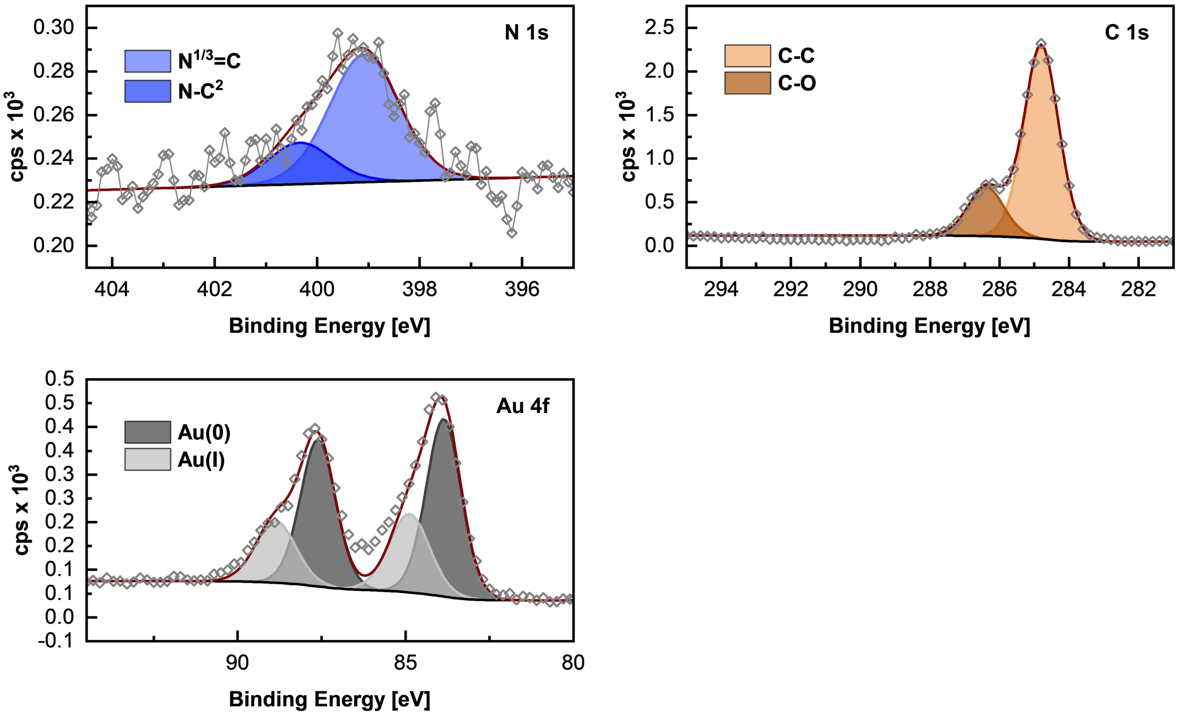

Figure S14 High resolution XPS spectra of N 1s, C 1s and Au 4f regions of **AuNP-4** obtained by procedure **P3**.

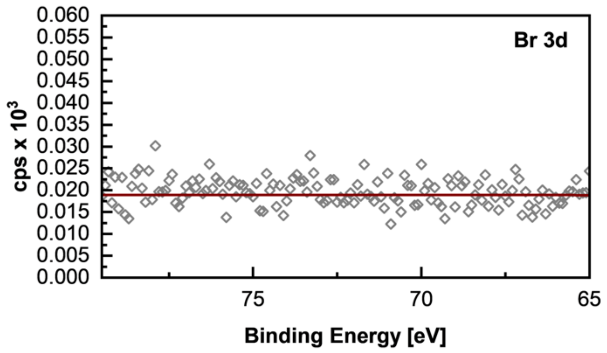

Figure S15 High resolution XPS spectrum of Br 3d region of **AuNP-3** obtained by procedure **P4.**

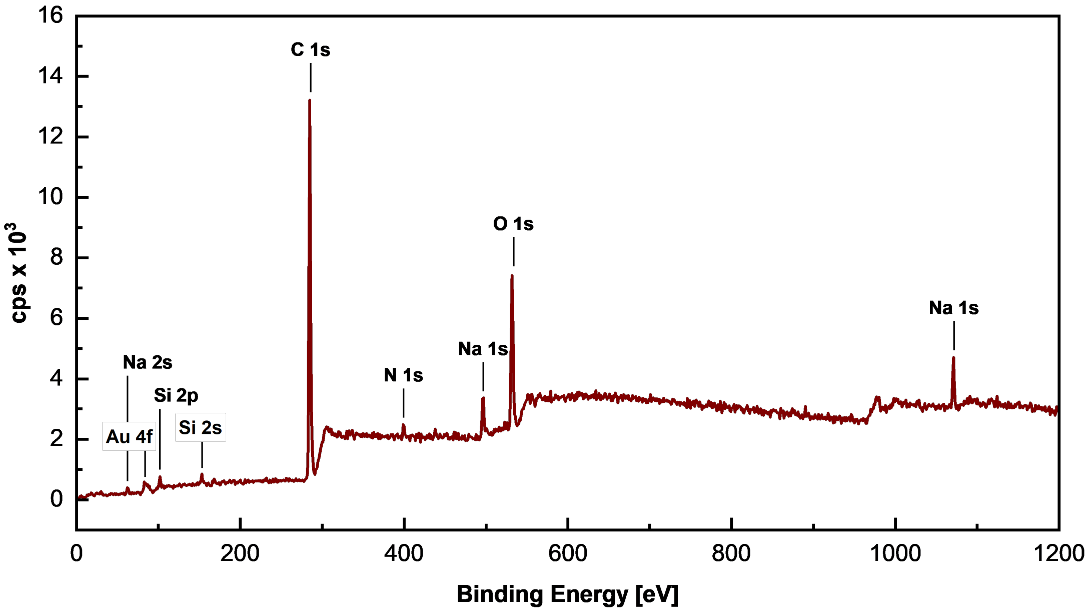

Figure S16 XPS survey spectrum of **AuNP-3** obtained by procedure **P4**.

TEM Micrographs

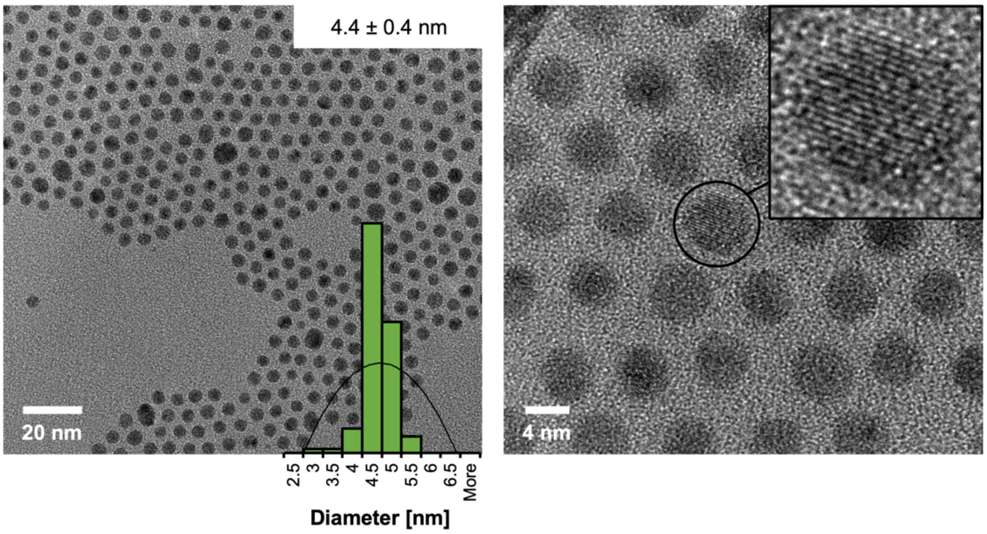

Figure S17 TEM micrographs of **OAm@AuNP**s.

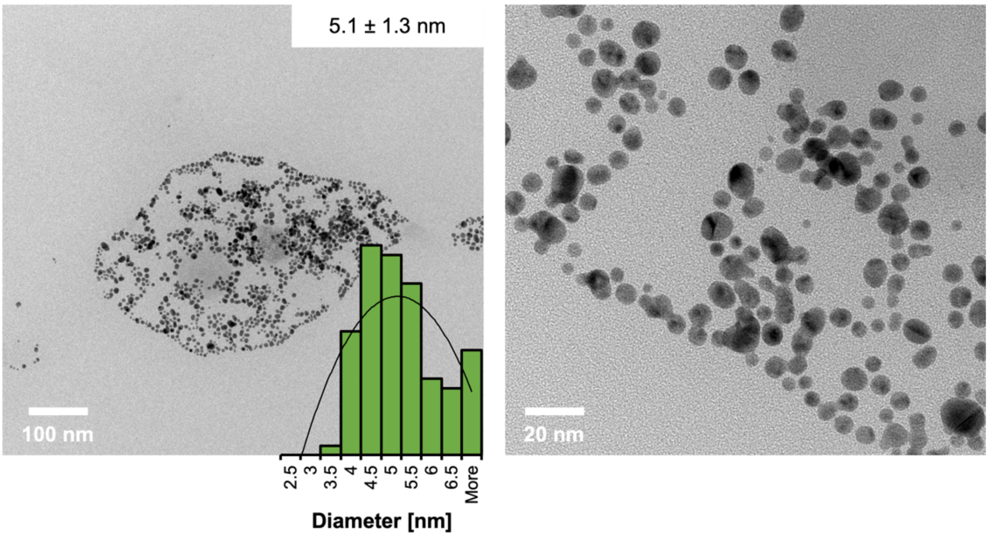

Figure S18 TEM micrographs of **AuNP-2** obtained by procedure **P2**.

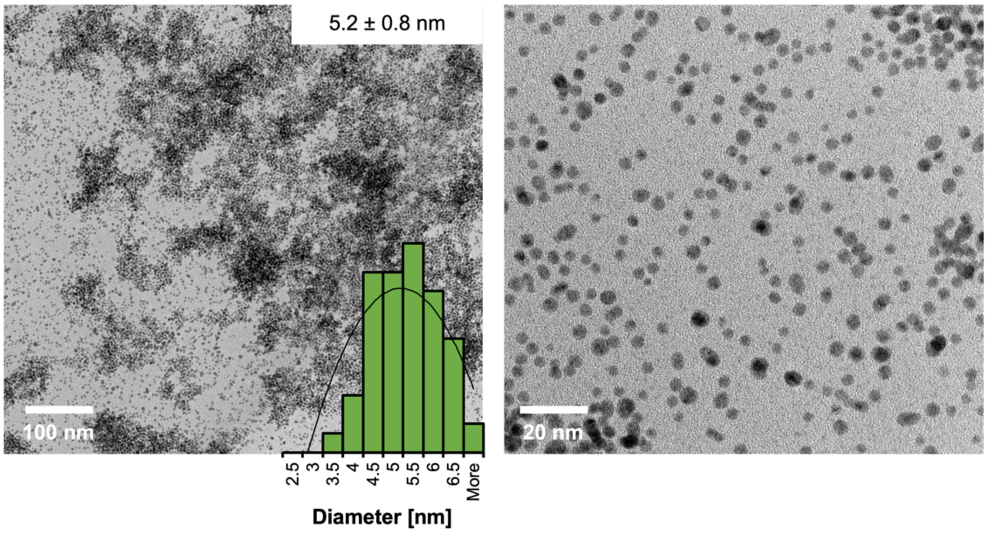

Figure S19 TEM micrographs of **AuNP-2** obtained by procedure $\mathbf{P2}_{\Delta}$.

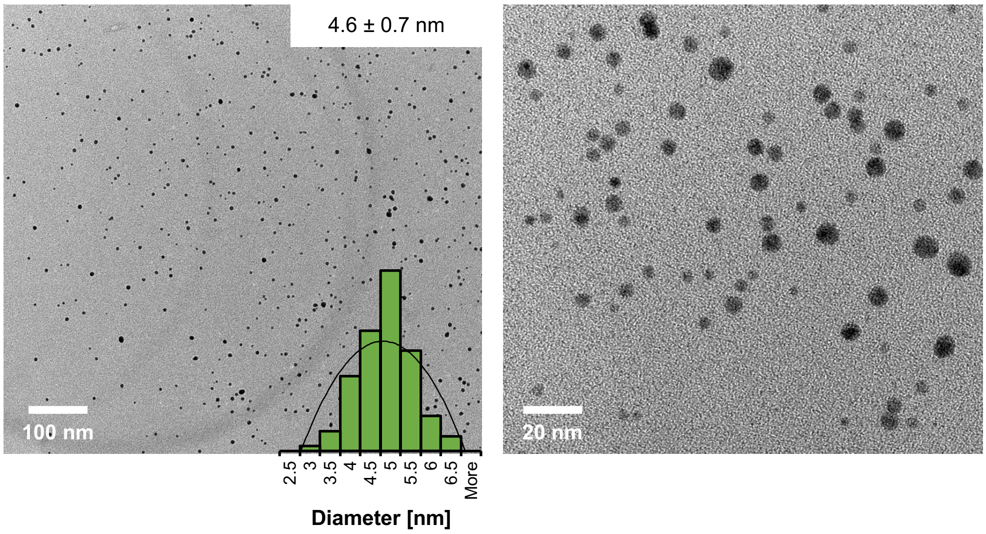

Figure S20 TEM micrographs of **AuNP-3** obtained by procedure **P3**.

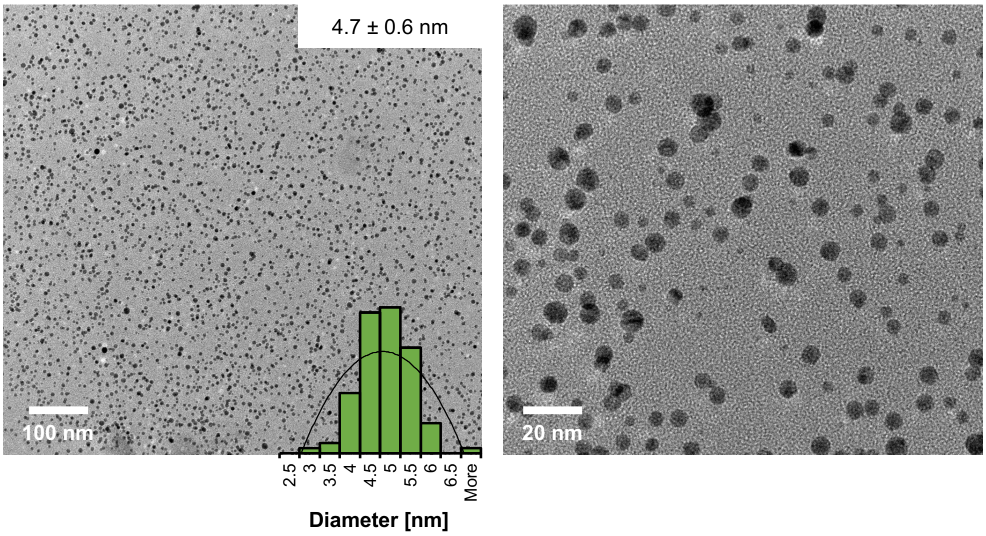

Figure S21 TEM micrographs of **AuNP-4** obtained by procedure **P3**.

UV-Vis Spectra in THF and H_2O

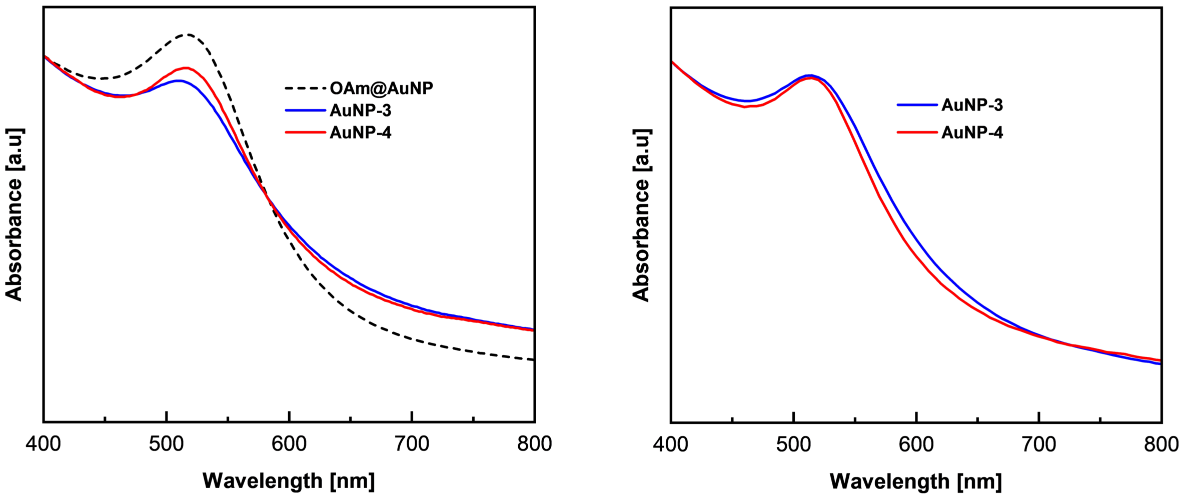

Figure S22 UV-Vis Spectra of **OAm@AuNP**s and PEG-NHC@AuNPs.

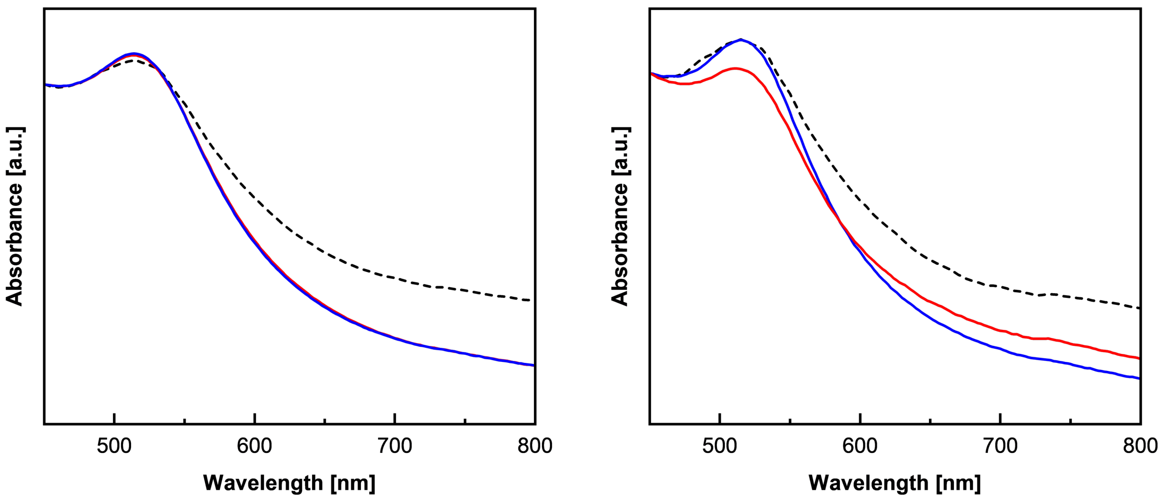

Figure S23 Normalized UV-Vis Spectra of AuNPs obtained by **P3** in H_2O. **AuNP-3** (*left*) and **AuNP-4** (*right*), both containing trace after dialysis (---), drying and redispersion (–) as well as heat exposure for 4 h at 40 °C (–)

Dynamic Light Scattering

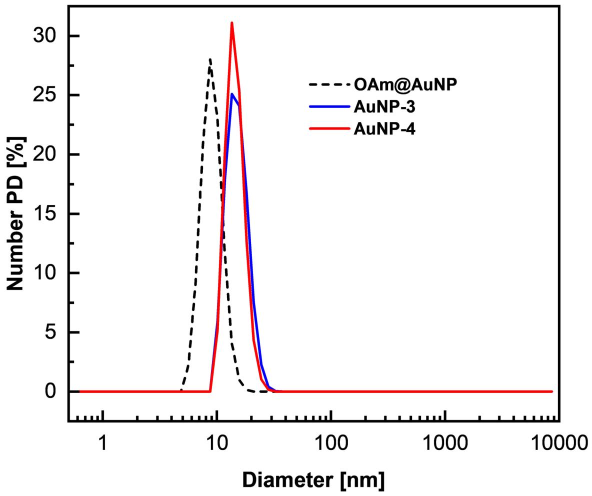

Figure S24 DLS traces of **OAm@AuNP** in THF; **AuNP-3** and **AuNP-4** in H_2O.

Thermogravimetric Analysis and NHC Coverage

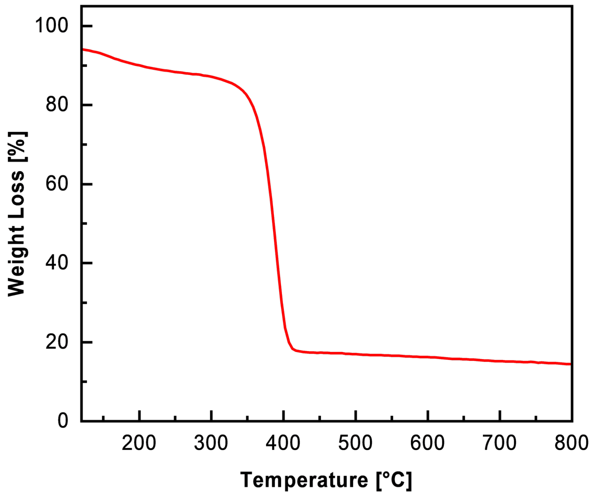

Figure S25 TGA curves of **AuNP-4**. TGA curve indicates an organic content of 69 wt%.

NHC coverage was approximated according to Johnson and coworkers[4] resulting in 1055 NHC per AuNP using the following formula and parameters without considering errors of measurement.

$$X = \frac{N_{NHC}}{N_{Au}}$$

$$NHC/AuNP = \left(\frac{V_{AuNP} \cdot \rho_{Au}}{M_{Au}}\right) \cdot N_A \cdot X$$

Ratio NHC/Au X; organic content from TGA = 69 wt%; radius (r_{AuNP}) = 2.3 nm; molar mass gold (M_{Au}) = 196.97 g mol^{-1}; density gold (ρ_{Au}) = 19.3 g cm^{-3}; molar mass NHC (M_{NHC}) 2089 g mol^{-1}.

Stability Studies – UV-Vis spectra, TEM micrographs and ^{1}H-NMR Spectra

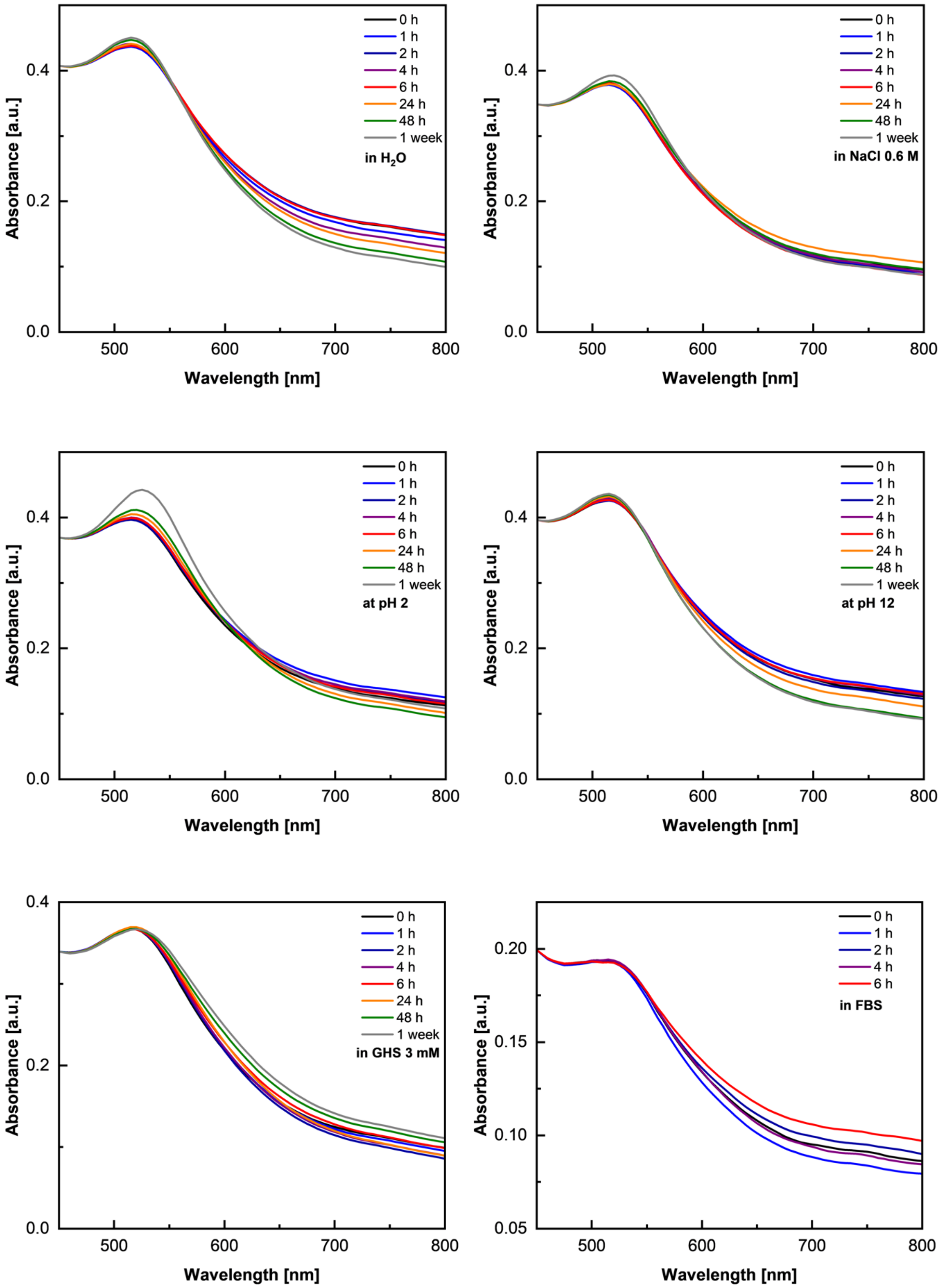

Figure S26 UV-Vis stability study of **AuNP-3** under various conditions.

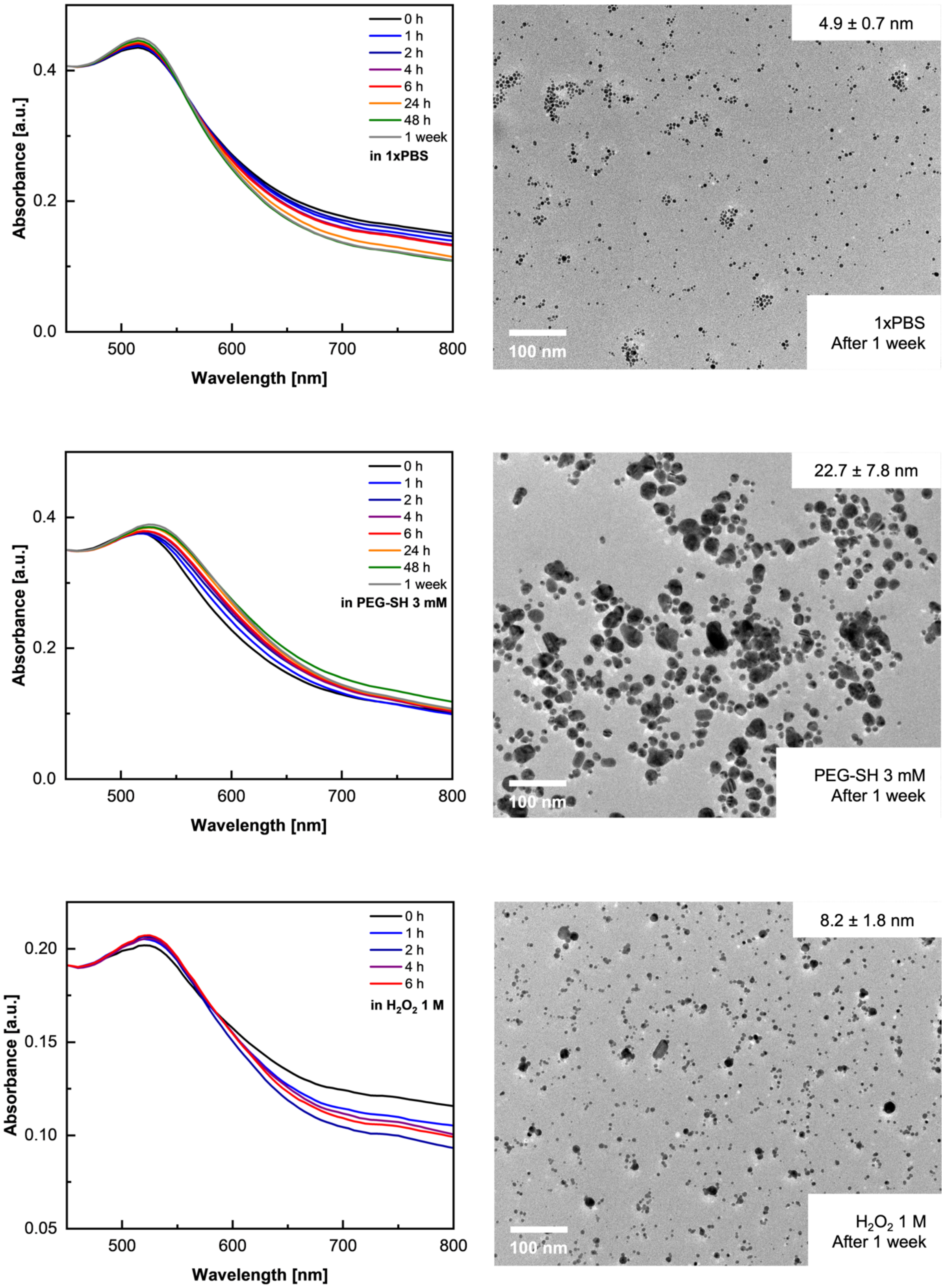

Figure S27 UV-Vis stability study of **AuNP-3** under various conditions and corresponding TEM micrographs.

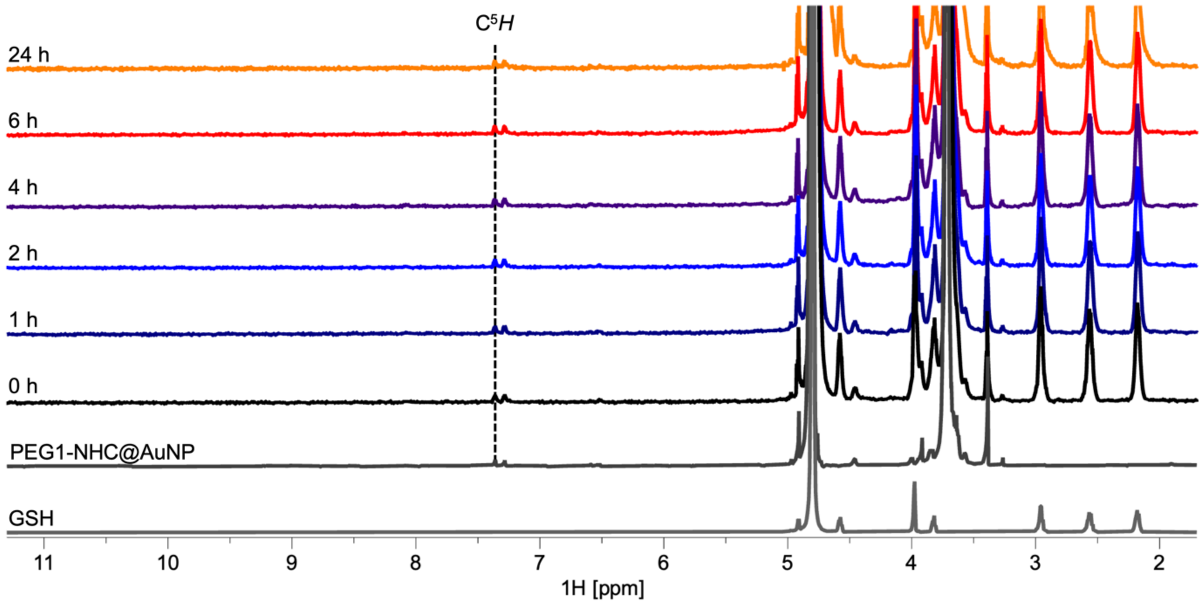

Figure S28 ^{1}H-NMR (D_2O, 500 MHz) kinetics of **AuNP-3** against GSH (3 mM) over a period of 24 h.

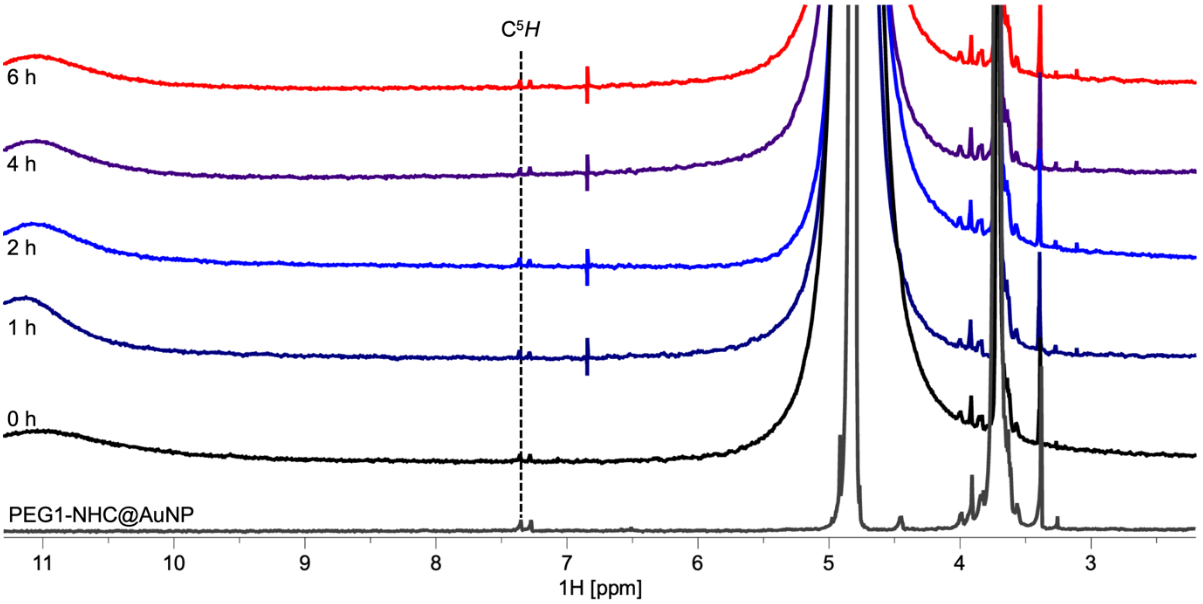

Figure S29 ^{1}H-NMR (D_2O, 500 MHz) kinetics of **AuNP-3** against H_2O_2 (1 M) over a period of 6 h.

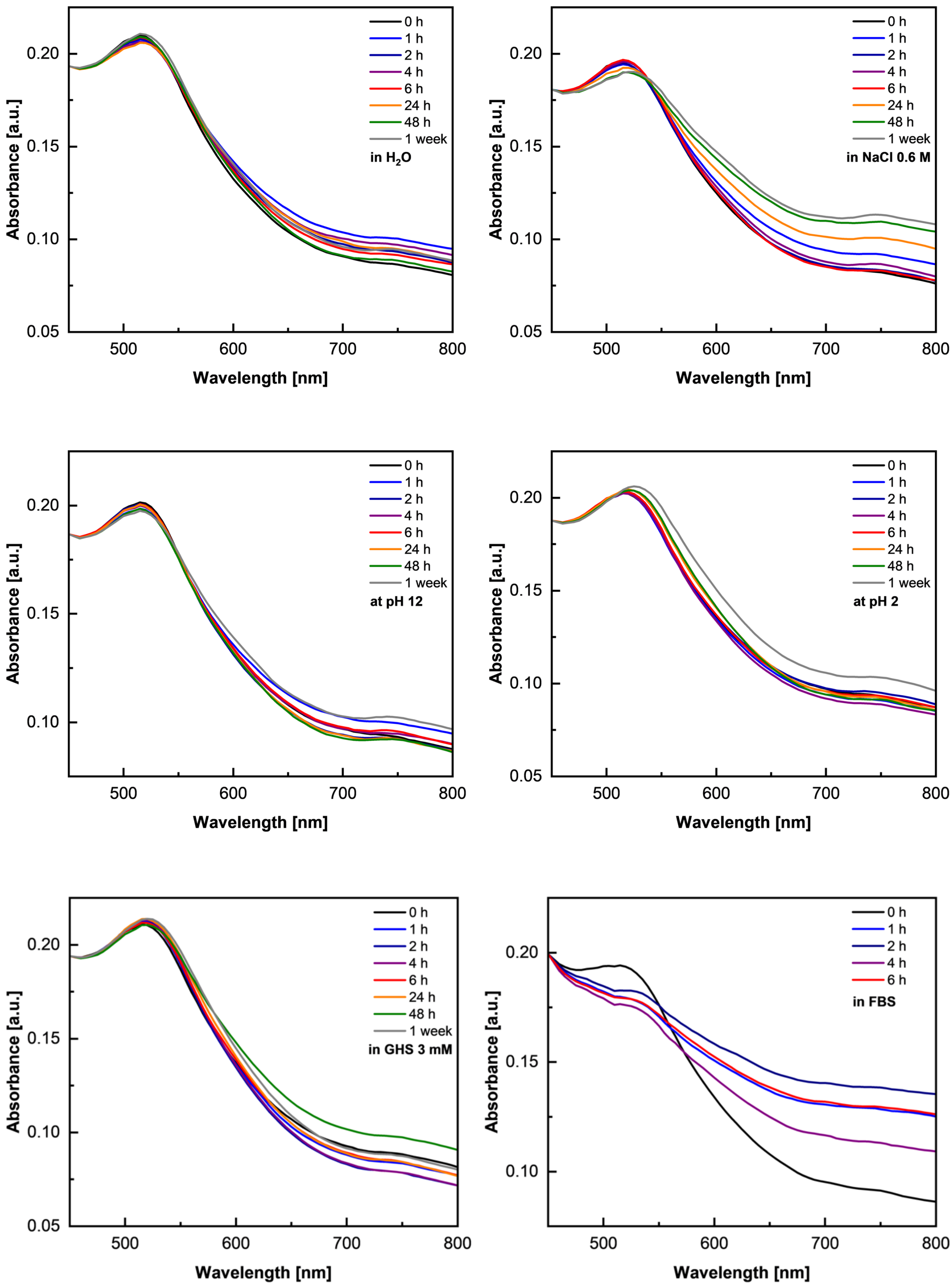

Figure S30 UV-Vis stability study of **AuNP-4** under various conditions.

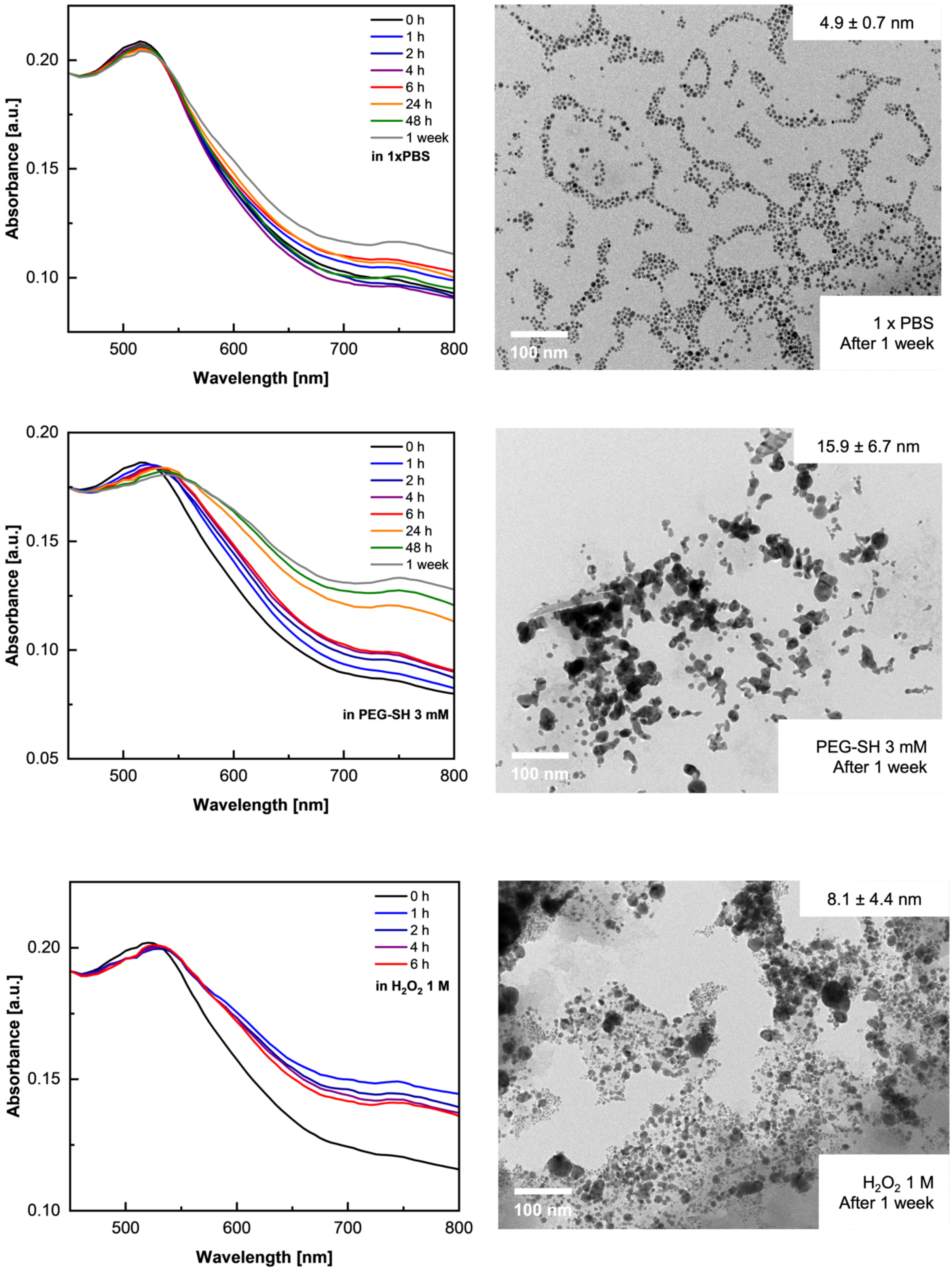

Figure S31 UV-Vis stability study of **AuNP-4** under various conditions and corresponding TEM micrographs.

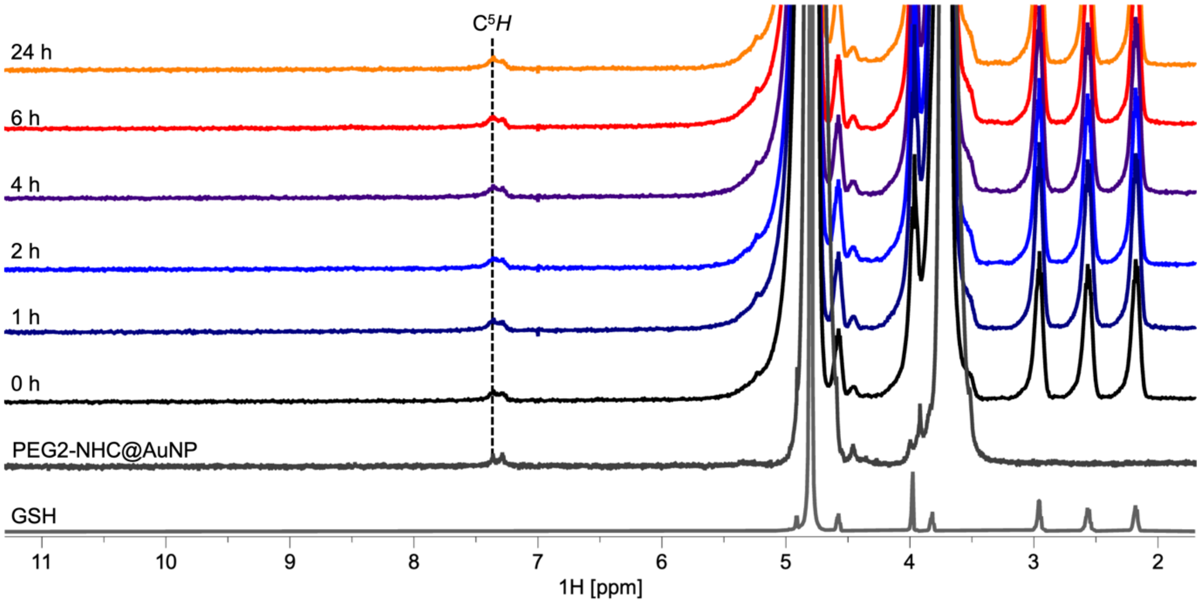

Figure S32 ^{1}H-NMR (D_2O, 500 MHz) kinetics of **AuNP-4** against GSH (3 mM) over a period of 24 h.

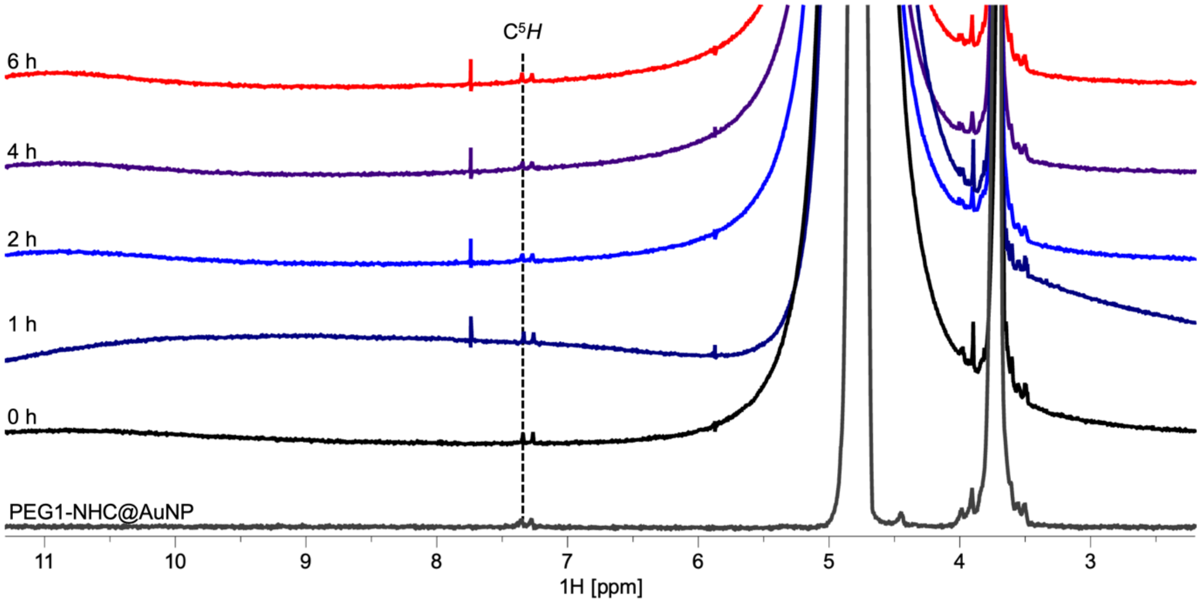

Figure S33 ^{1}H-NMR (D_2O, 500 MHz) kinetics of **AuNP-4** against H_2O_2 (1 M) over a period of 6 h.

Characterization of Bottom-up AuNPs

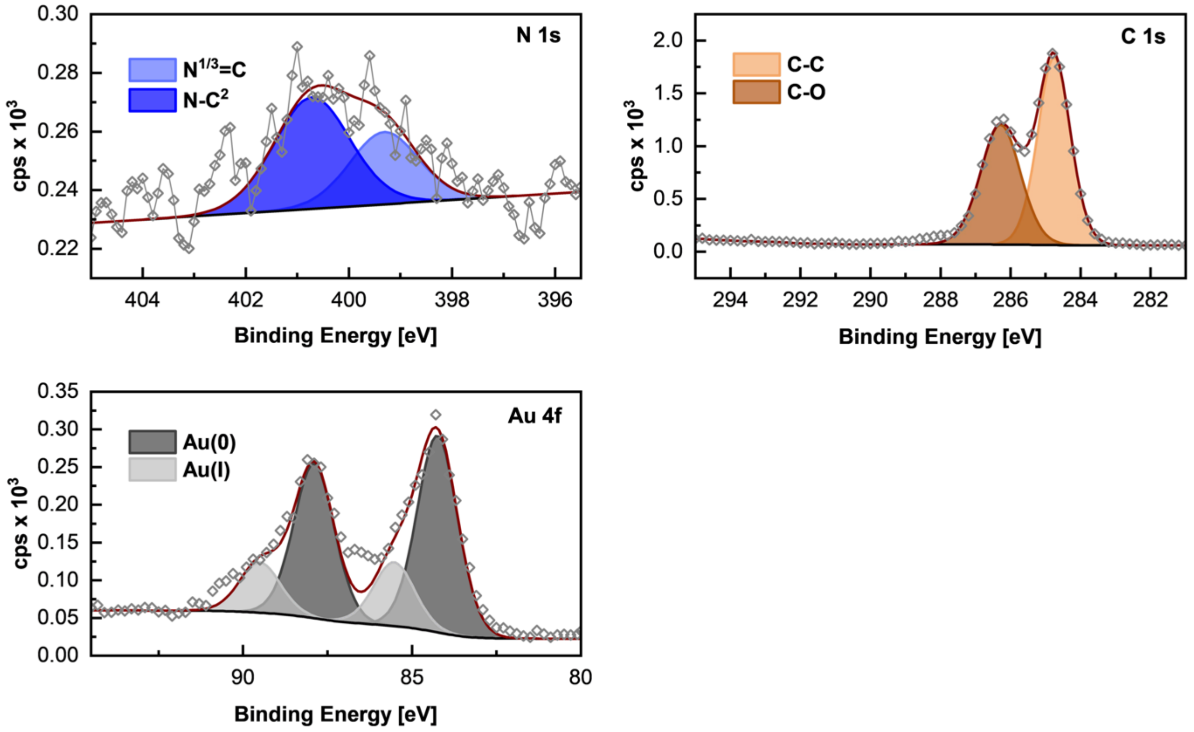

Figure S34 High resolution XPS spectra of N 1s, C 1s and Au 4f regions of **AuNP-3^{BU}** obtained by procedure **P5**.

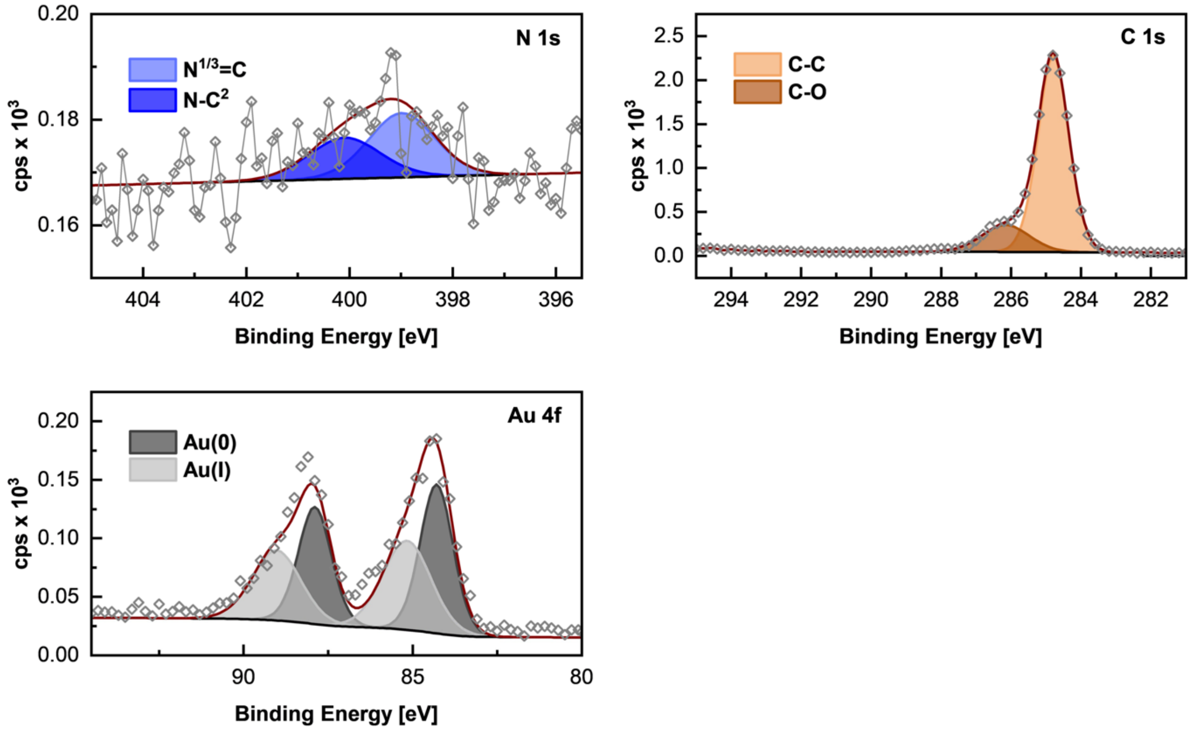

Figure S35 High resolution XPS spectra of N 1s, C 1s and Au 4f regions of **AuNP-4^{BU}** obtained by procedure **P5**.

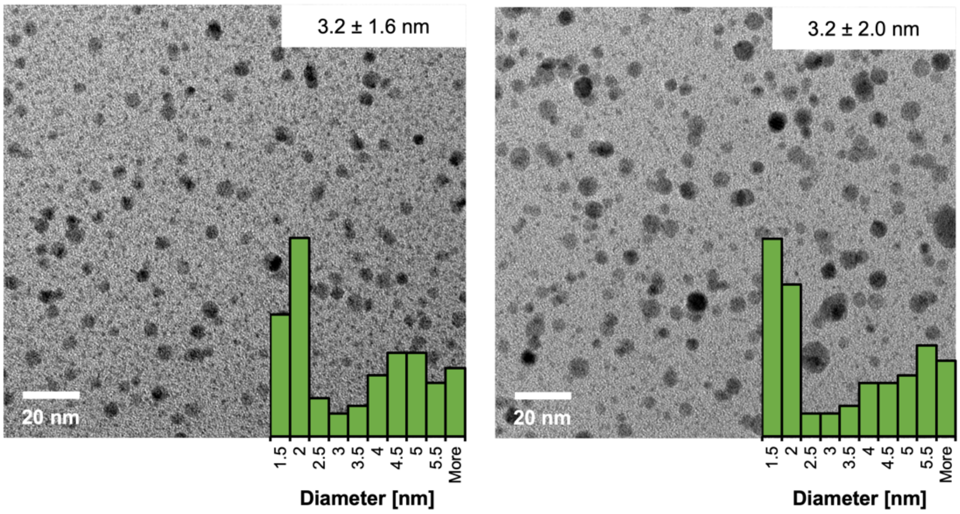

Figure S36 TEM micrographs of **AuNP-3^BU^** (*left*) and **AuNP-4^BU^** (*right*), both micrographs indicate two particle populations at ~1.5 and 5 nm, resulting in a broad size distribution.

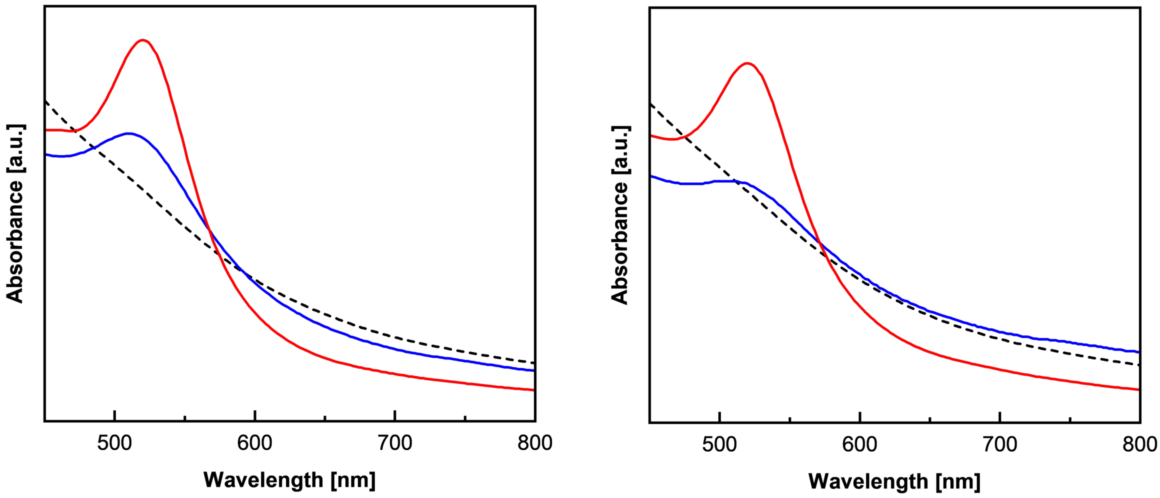

Figure S37 Normalized UV-Vis Spectra of AuNPs obtained by **P5** in H_2O. **AuNP-3^BU^** (*left*) and **AuNP-4^BU^** (*right*), both containing trace after dialysis (---), drying and redispersion (–) as well as heat exposure for 4 h at 40 °C (–).

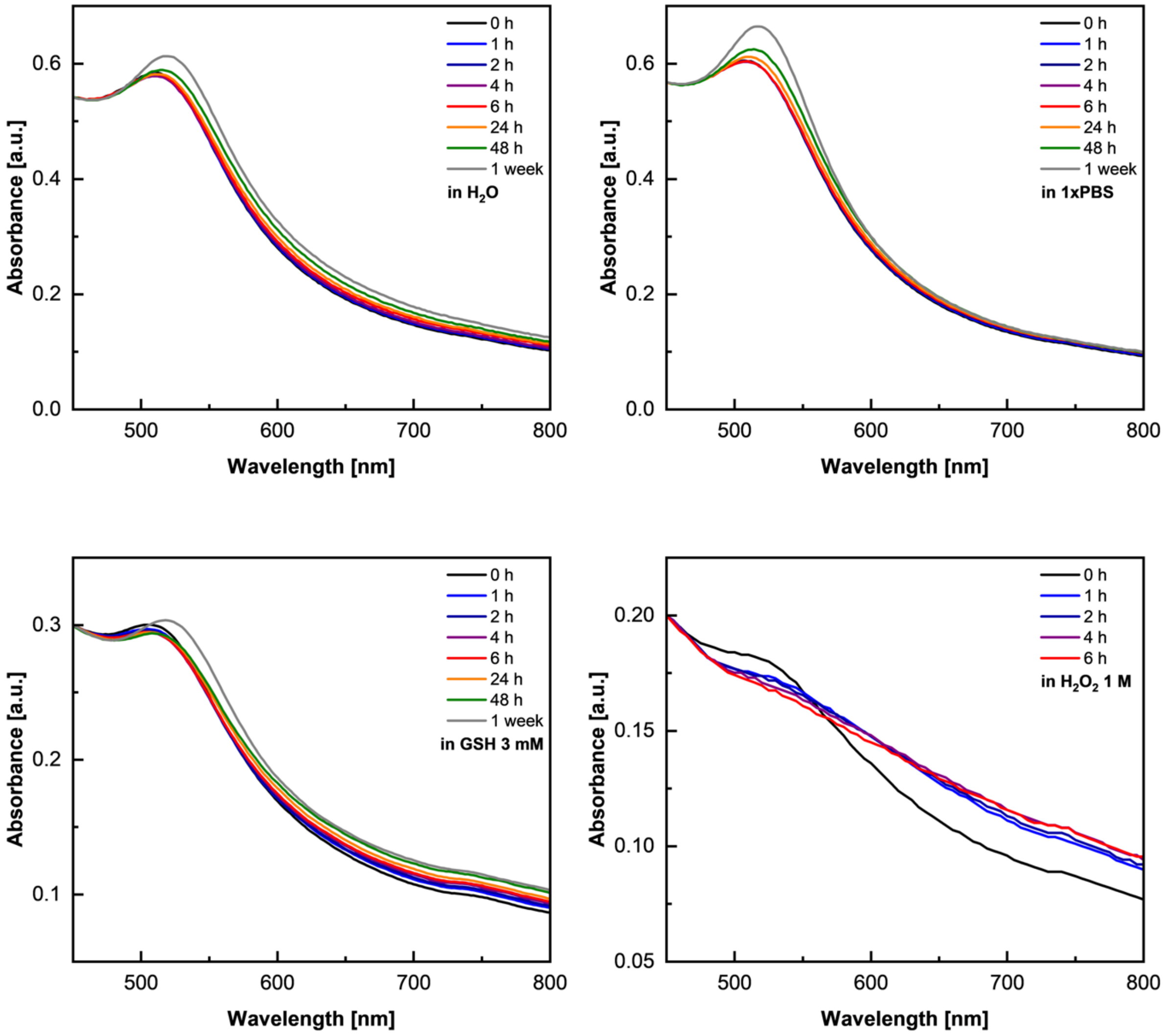

Figure S38 UV-Vis stability study of **AuNP-3^{BU}** exposed to various conditions.

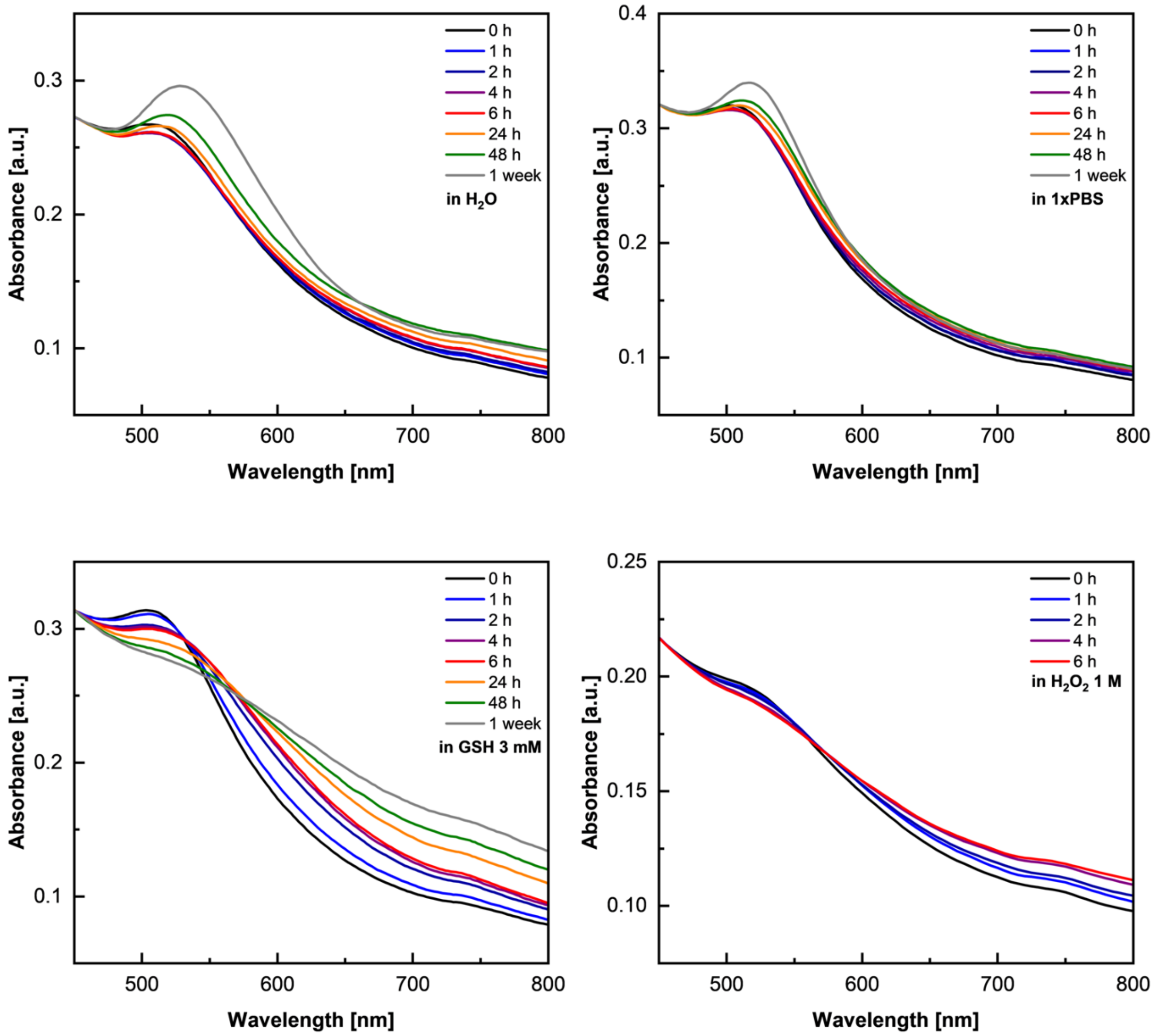

Figure S39 UV-Vis stability study of **AuNP-4**^{BU} exposed to various conditions.

Characterization of Click Conjugation Procedures

CuAAC

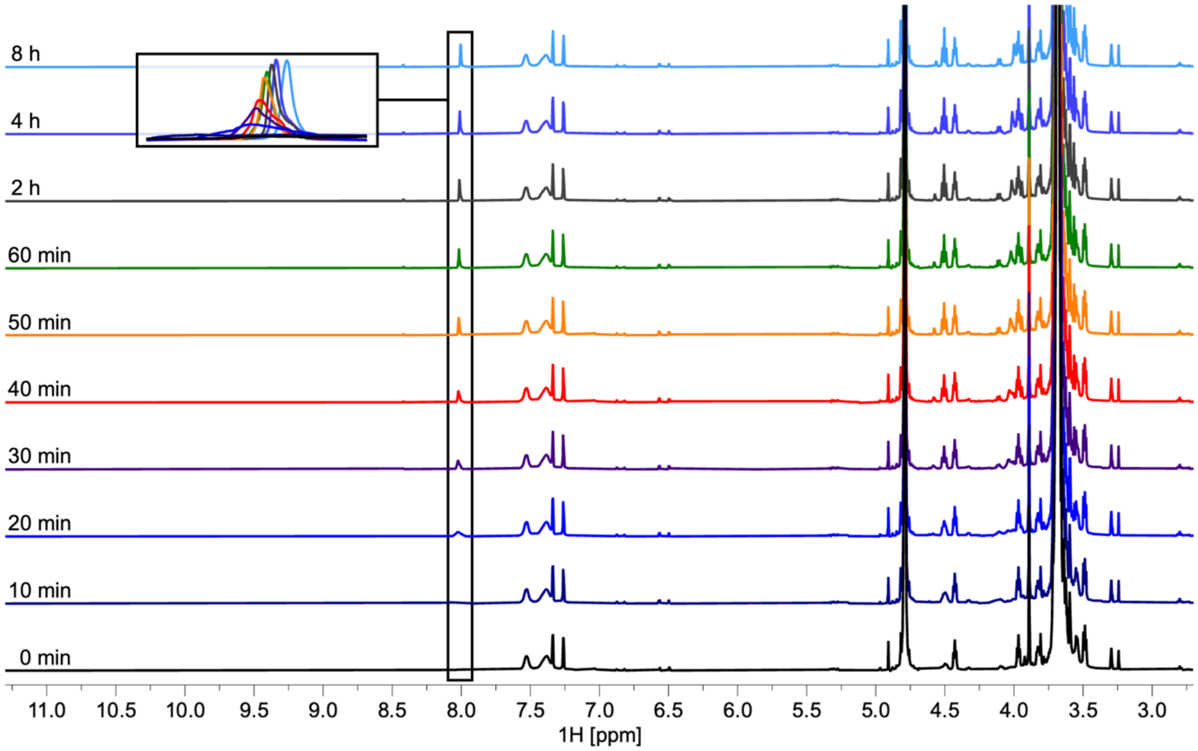

Figure S40 ^{1}H-NMR (500 MHz, D_2O) kinetics of **AuNP-4** reacting with phenylacetylene under CuAAC conditions. Insert = close up of forming triazole proton (C^7H) peak at 8.0 ppm.[4, 10]

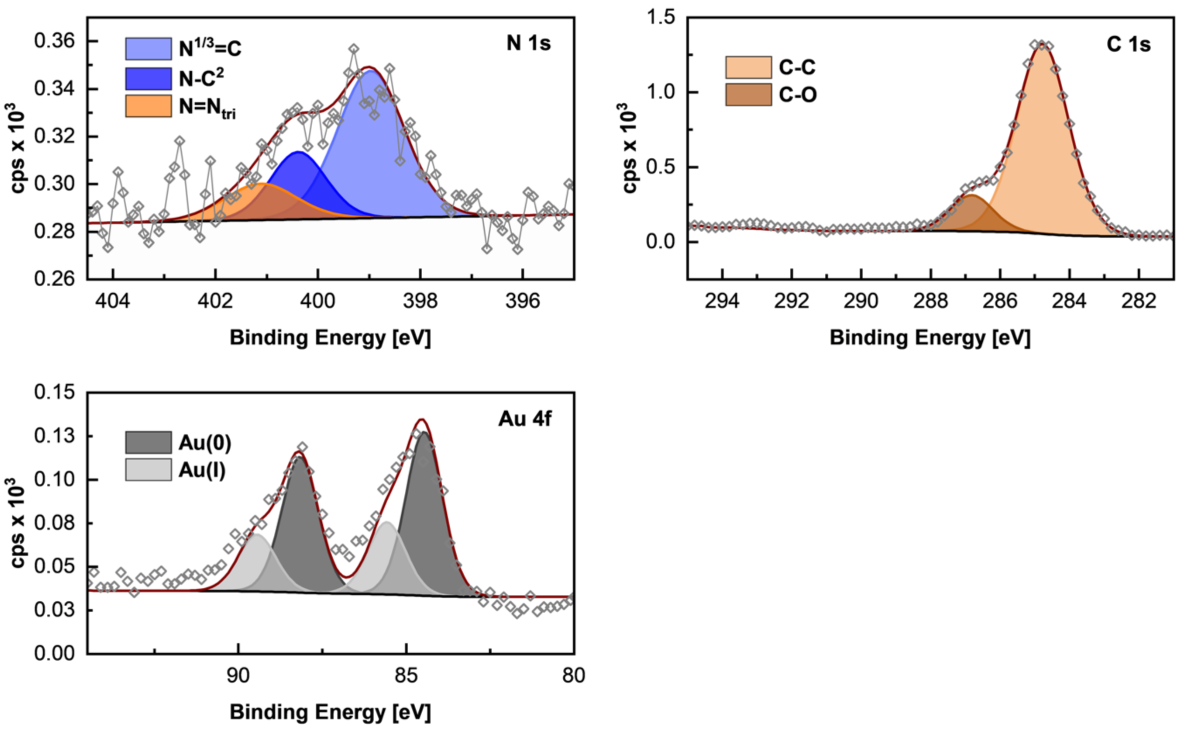

Figure S41 High resolution XPS spectra of N 1s, C 1s and Au 4f regions of clicked **AuNP-5**.

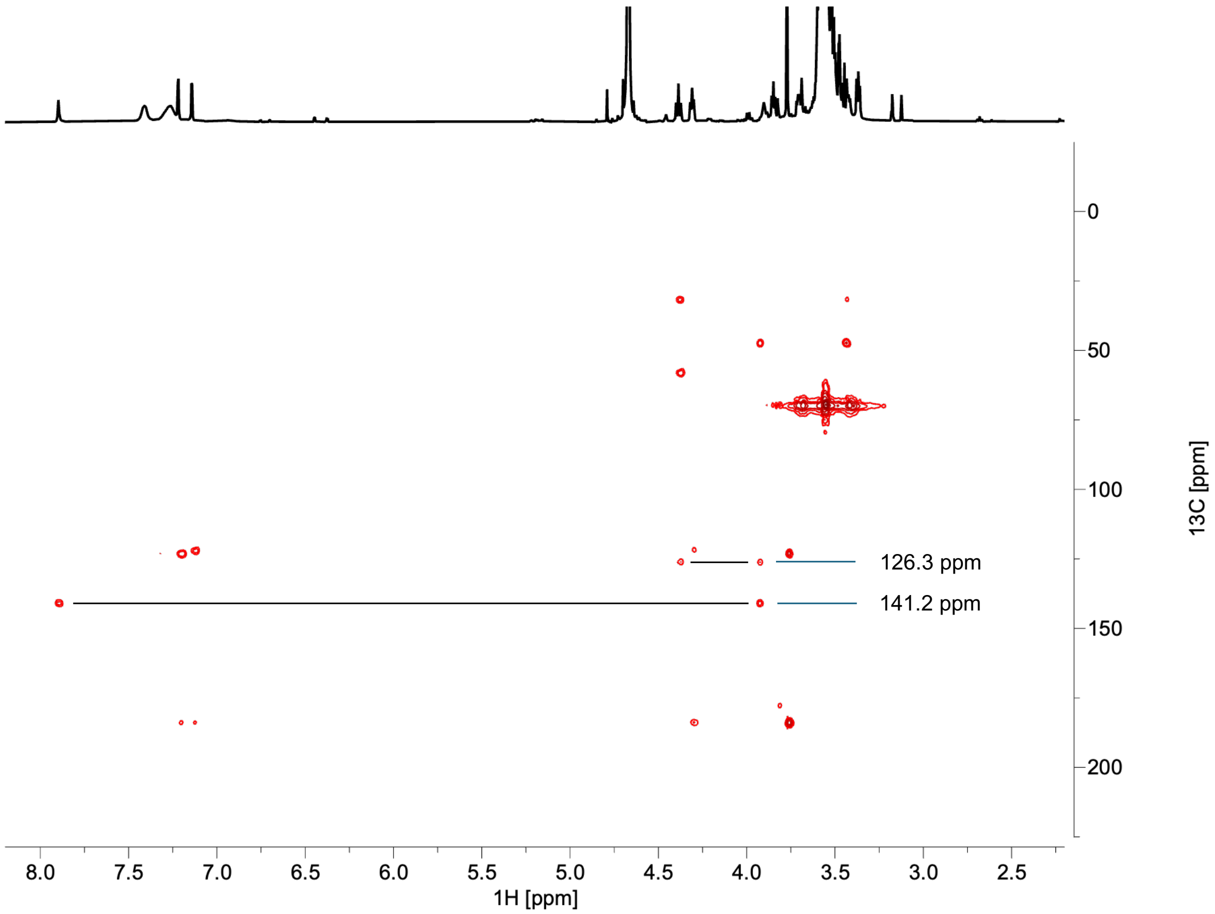

Figure S42 $^{1}H/^{13}C$-HMBC (500 MHz, D_2O) of **AuNP-4** with phenylacetylene under CuAAC conditions after 8 h.

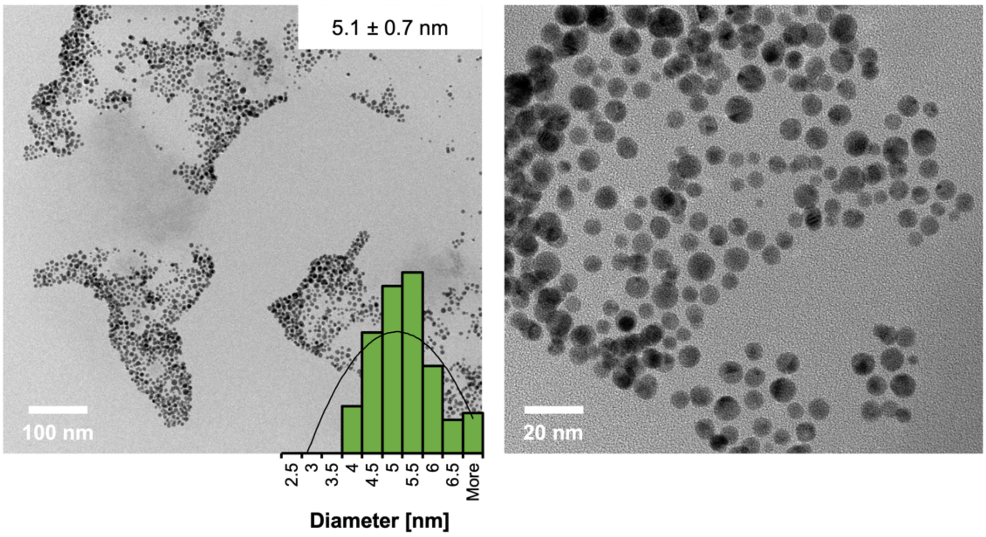

Figure S43 TEM micrograph of clicked **AuNP-5**.

SPAAC

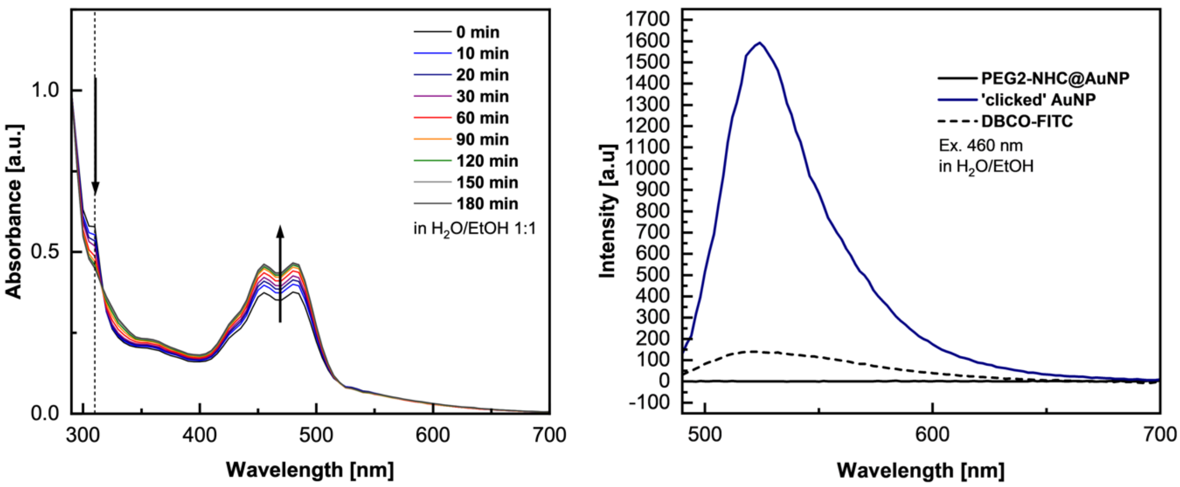

Figure S44 *Left:* UV-Vis kinetics of the reaction progress of **AuNP-4** with **DBCO-FITC** under SPAAC conditions. Reference line (**---**) at 310 nm indicates unbound DBCO specific absorbance.[11] *Right:* Emission spectra (ex. 460 nm) of clicked **AuNP-6** and as made **AuNP-4**; DBCO-FITC (**---**, em.$_{max}$ 522 nm) measured in concentration used in AuNP conjugation experiments.

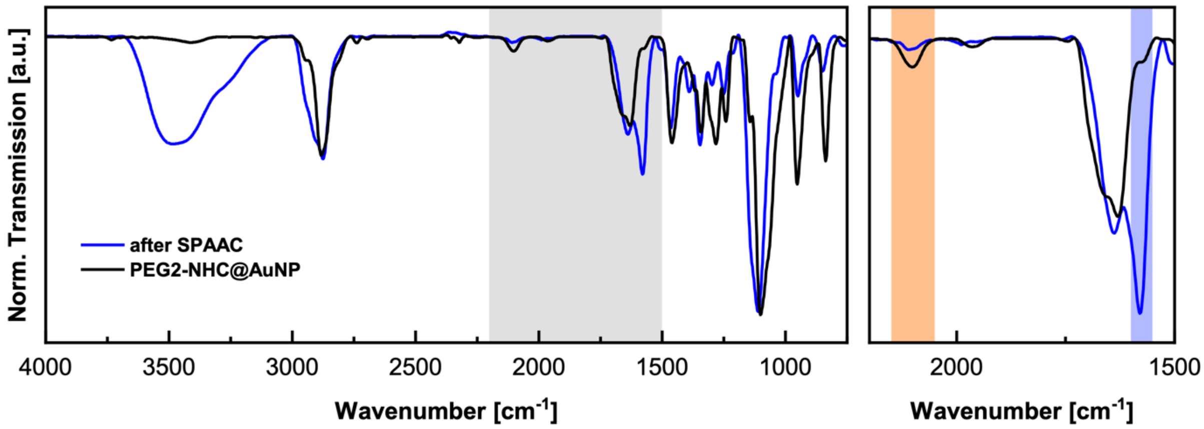

Figure S45 FT-IR Comparison of **AuNP-4** and clicked **AuNP-6** after SPAAC. SPAAC progress monitored *via* change of N_3 signal (■, 2100 cm^{-1}) and appearance of DBCO triazole related band (■, 1577 cm^{-1}).

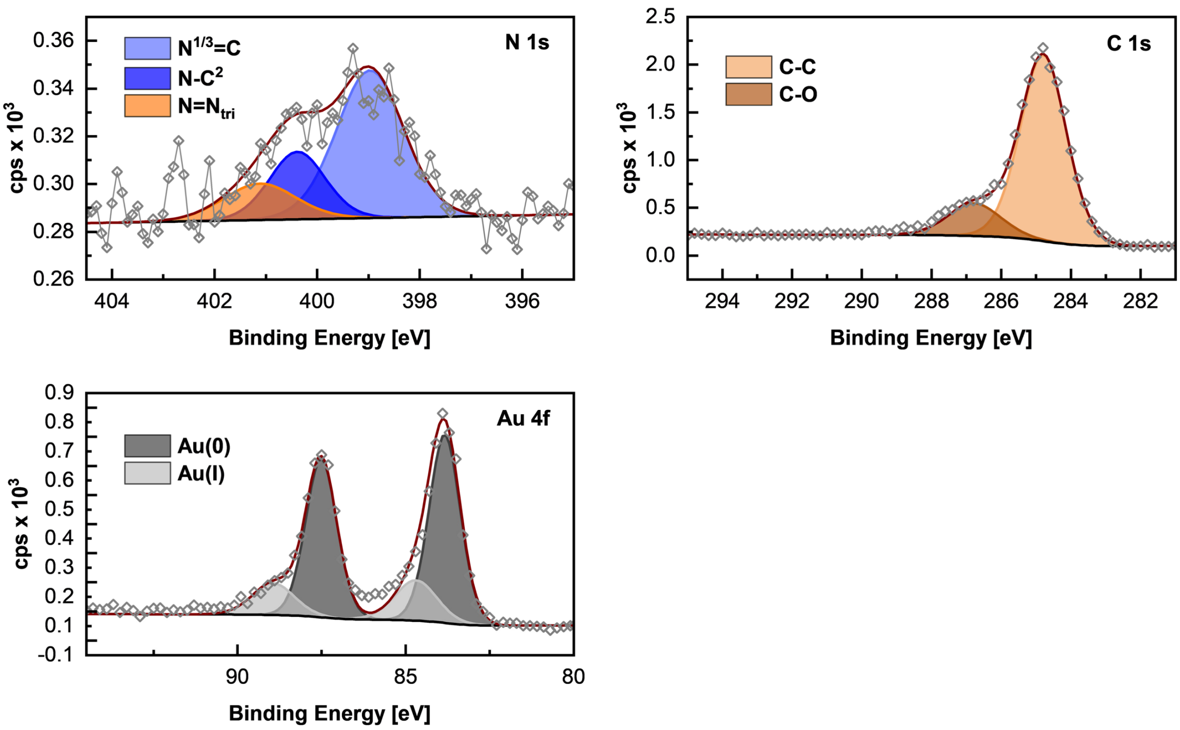

Figure S46 High resolution XPS spectra of N 1s, C 1s and Au 4f regions of clicked **AuNP-6**.

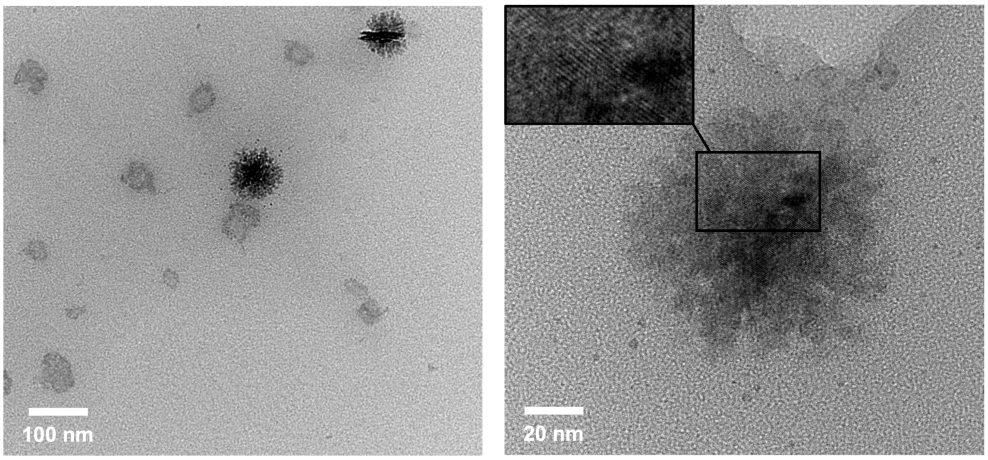

Figure S47 TEM micrograph of **AuNP-6**.

^{1}H- and ^{13}C-NMR Spectra

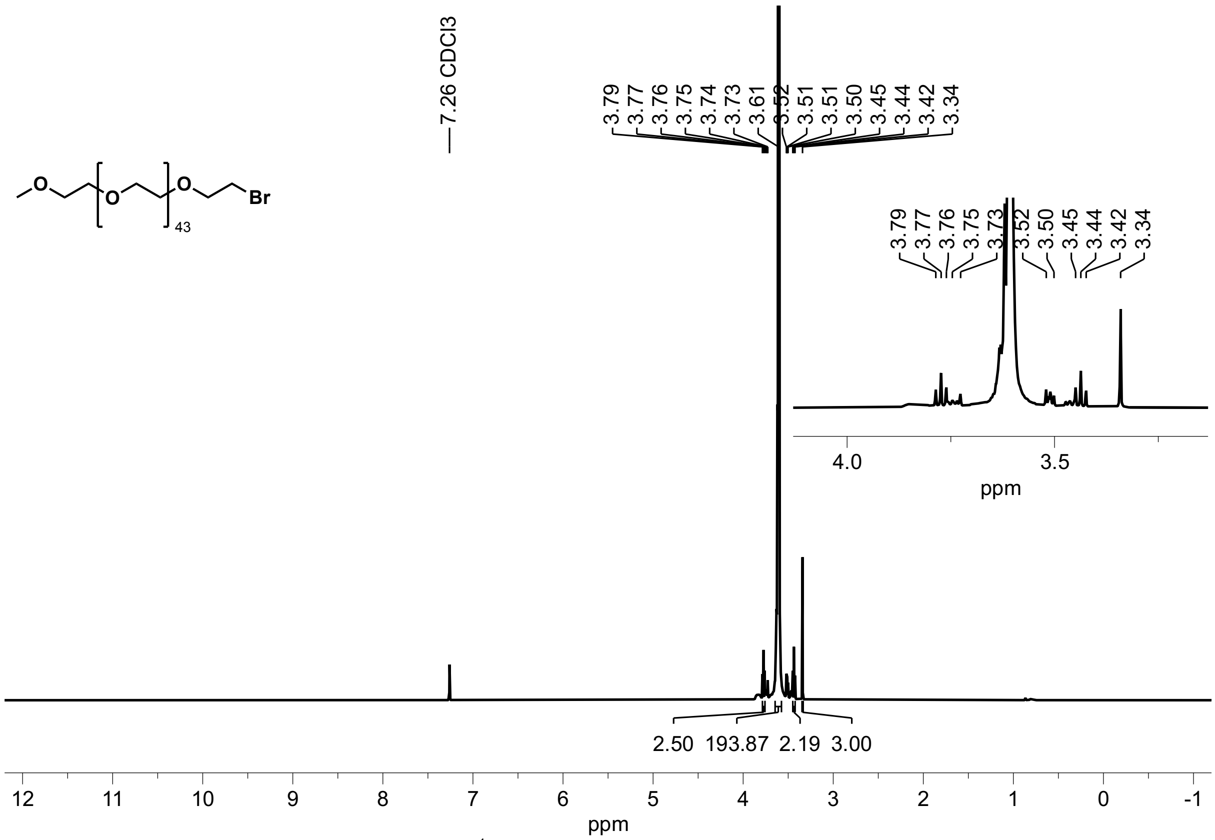

Figure S48 ^{1}H-NMR (500 MHz, $CDCl_3$) of **PEG1**.

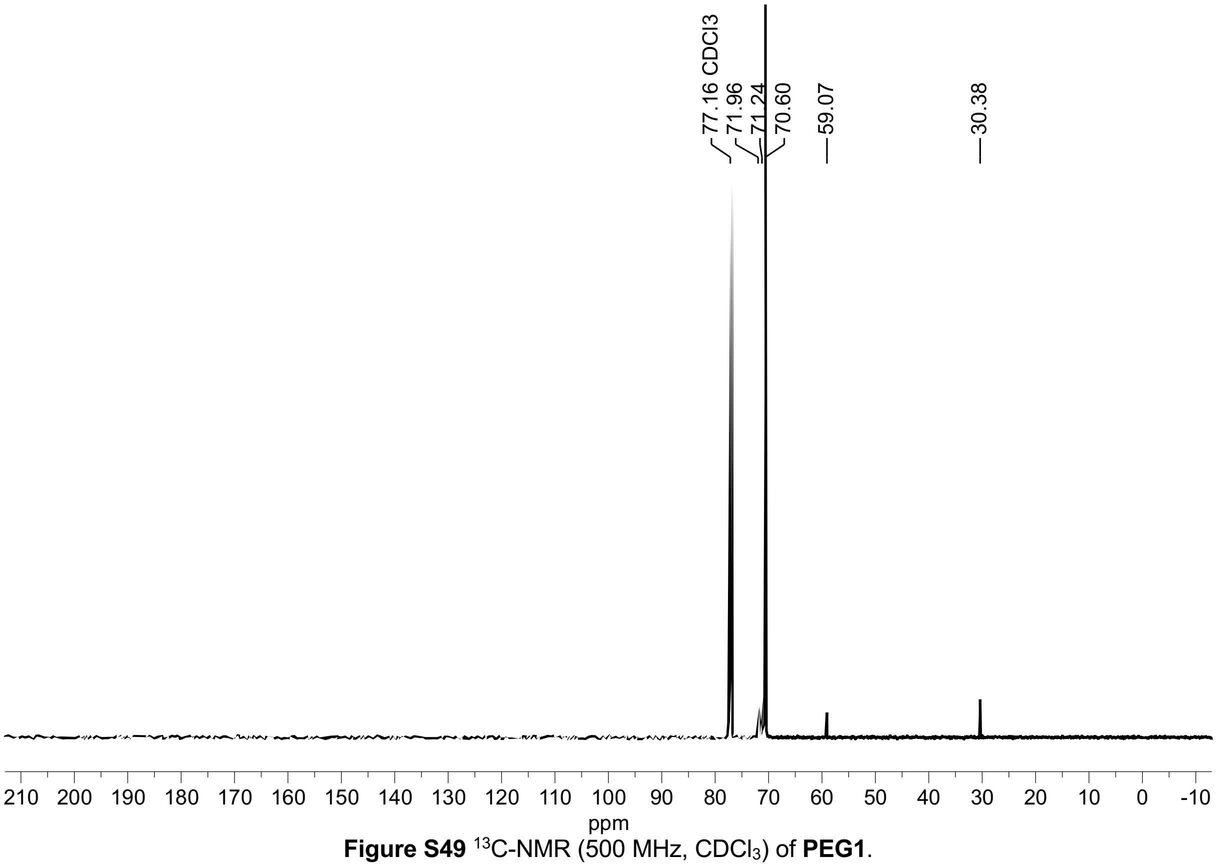

Figure S49 ^{13}C-NMR (500 MHz, $CDCl_3$) of **PEG1**.

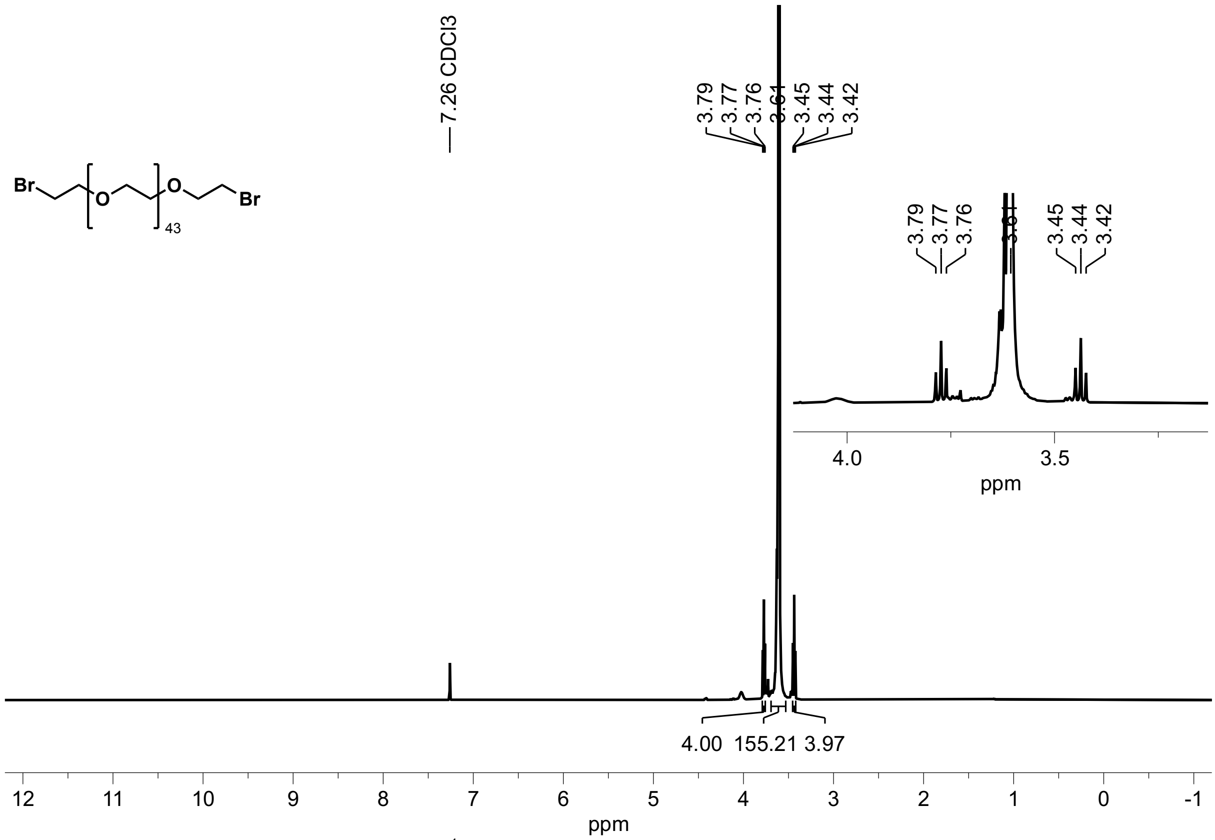

Figure S50 ^{1}H-NMR (500 MHz, $CDCl_3$) of **Br-PEG-Br**.

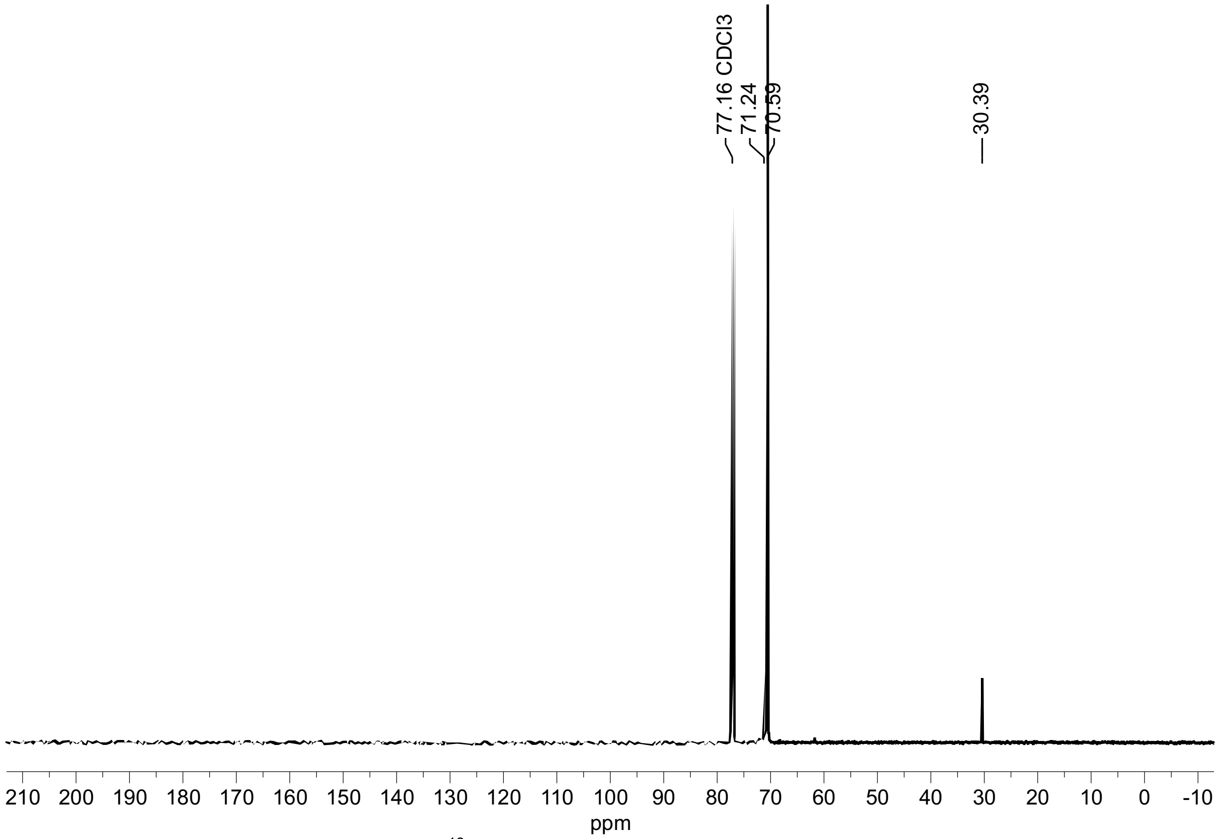

Figure S51 ^{13}C-NMR (500 MHz, $CDCl_3$) of **Br-PEG-Br**.

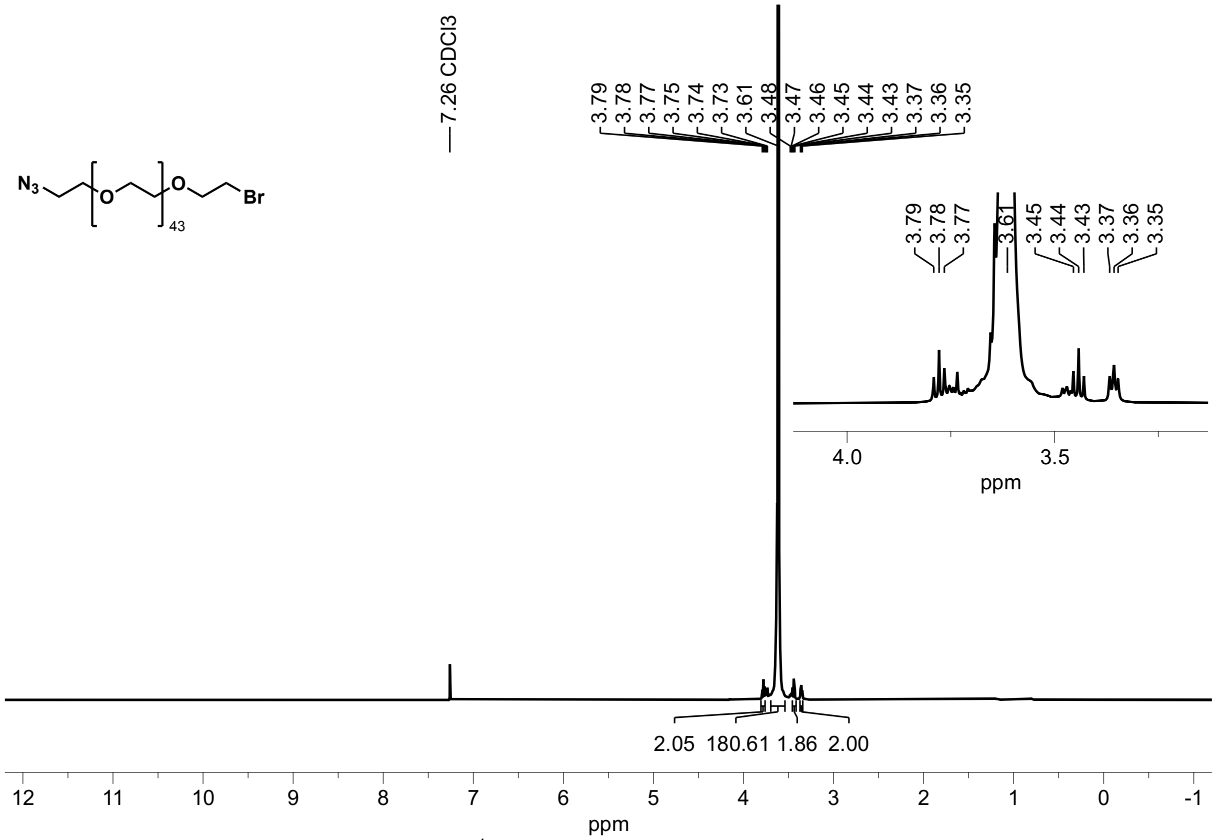

Figure S52 ^{1}H-NMR (500 MHz, $CDCl_3$) of **PEG2**.

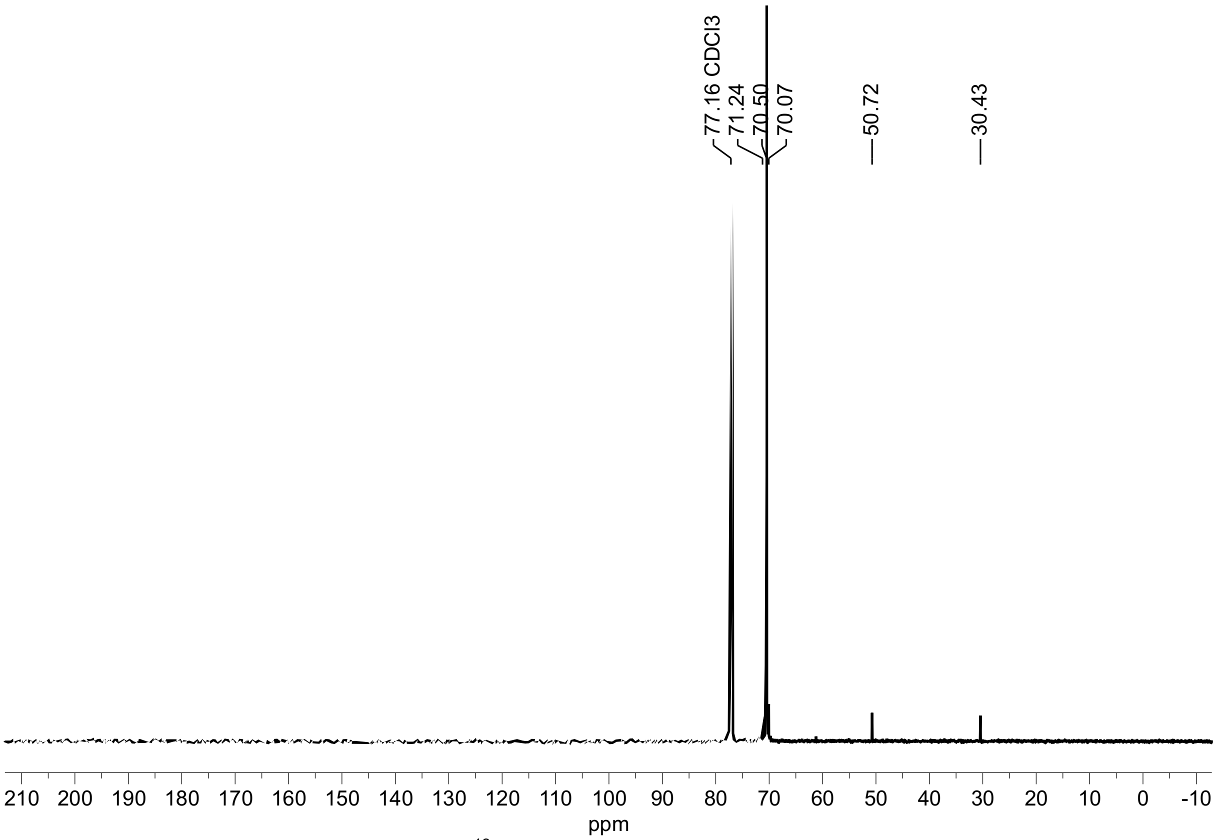

Figure S53 ^{13}C-NMR (500 MHz, $CDCl_3$) of **PEG2**.

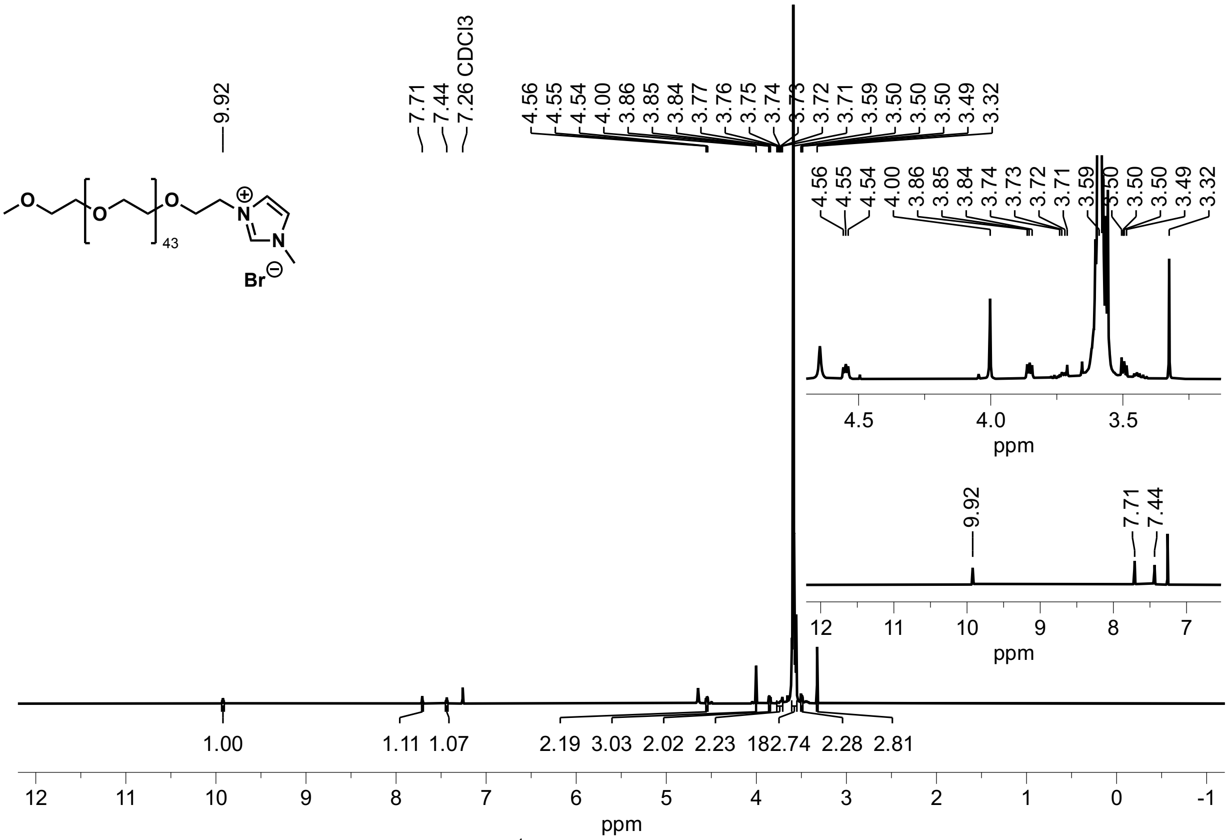

Figure S54 ^{1}H-NMR (500 MHz, $CDCl_3$) of **1**.

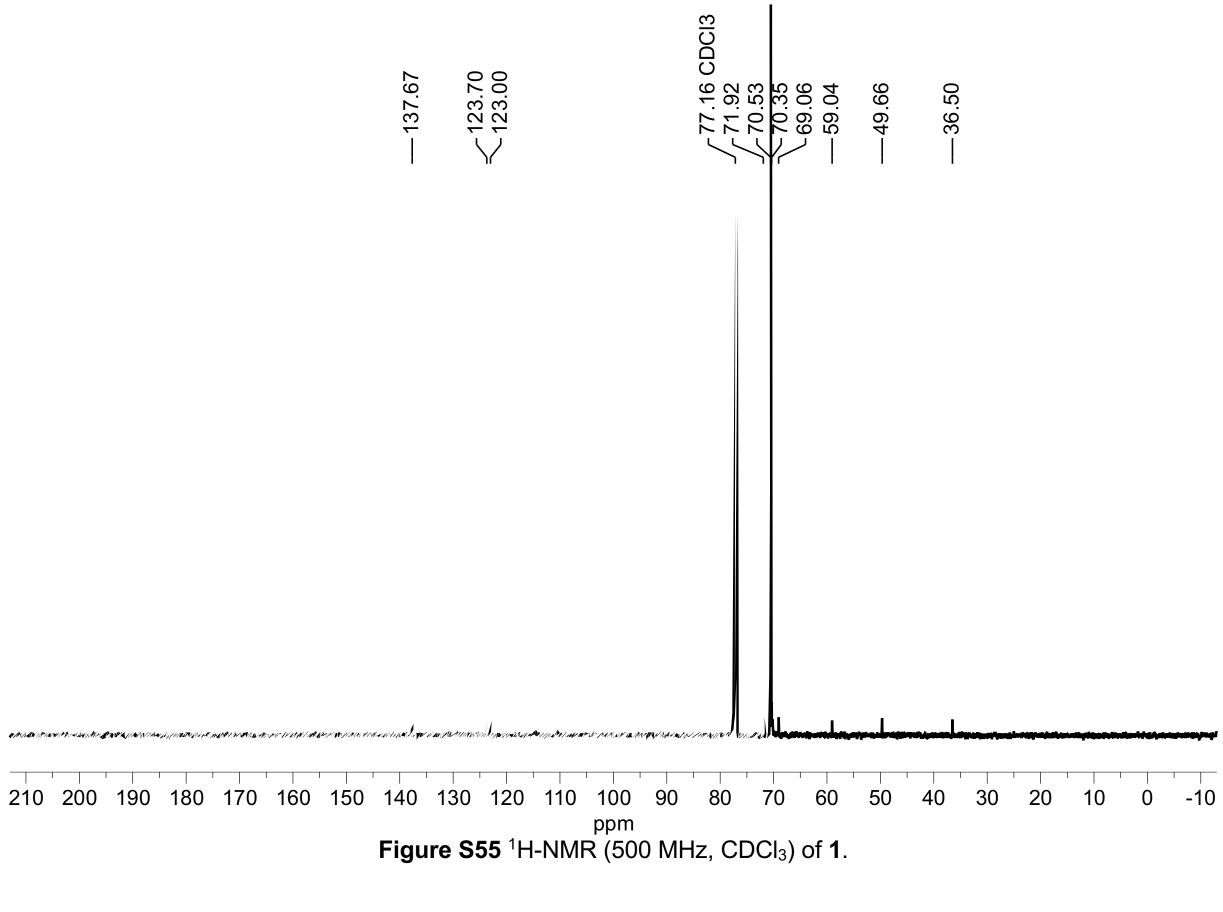

Figure S55 ^{1}H-NMR (500 MHz, $CDCl_3$) of **1**.

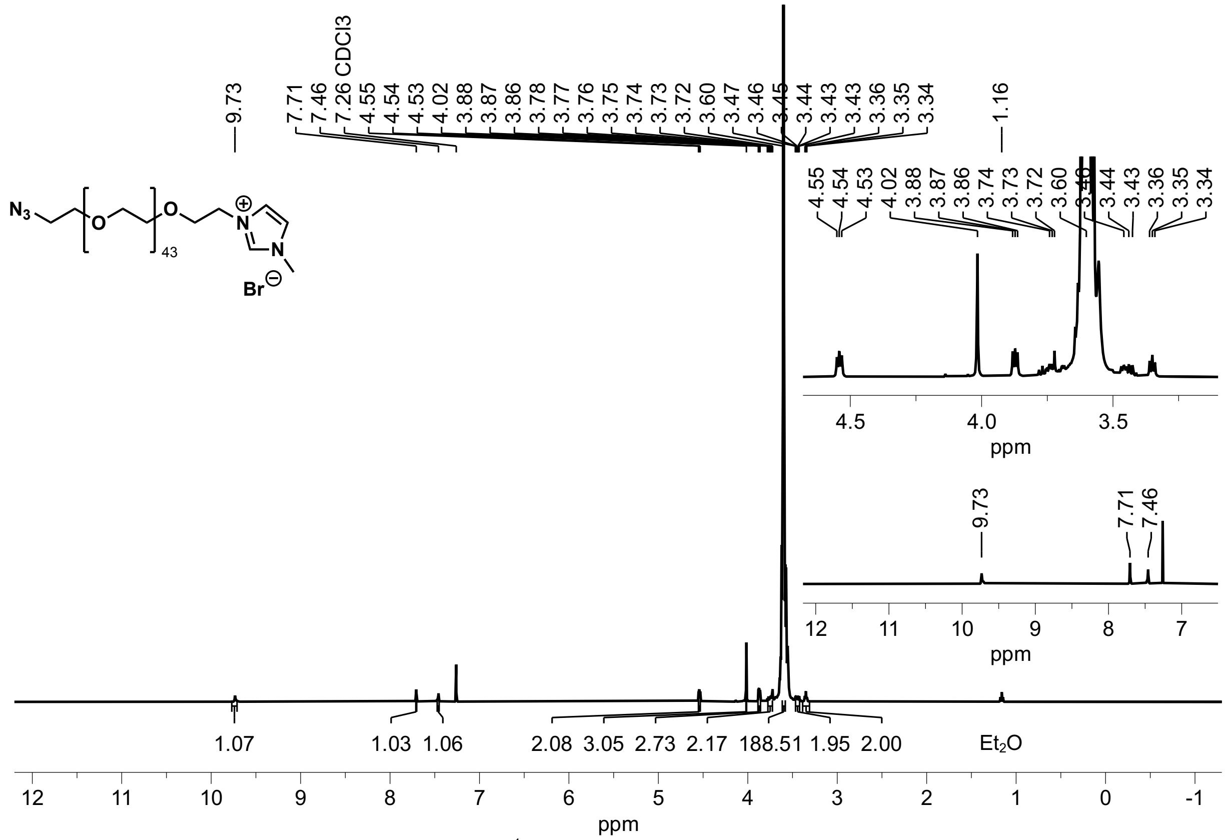

Figure S56 ^{1}H-NMR (500 MHz, $CDCl_3$) of **2**.

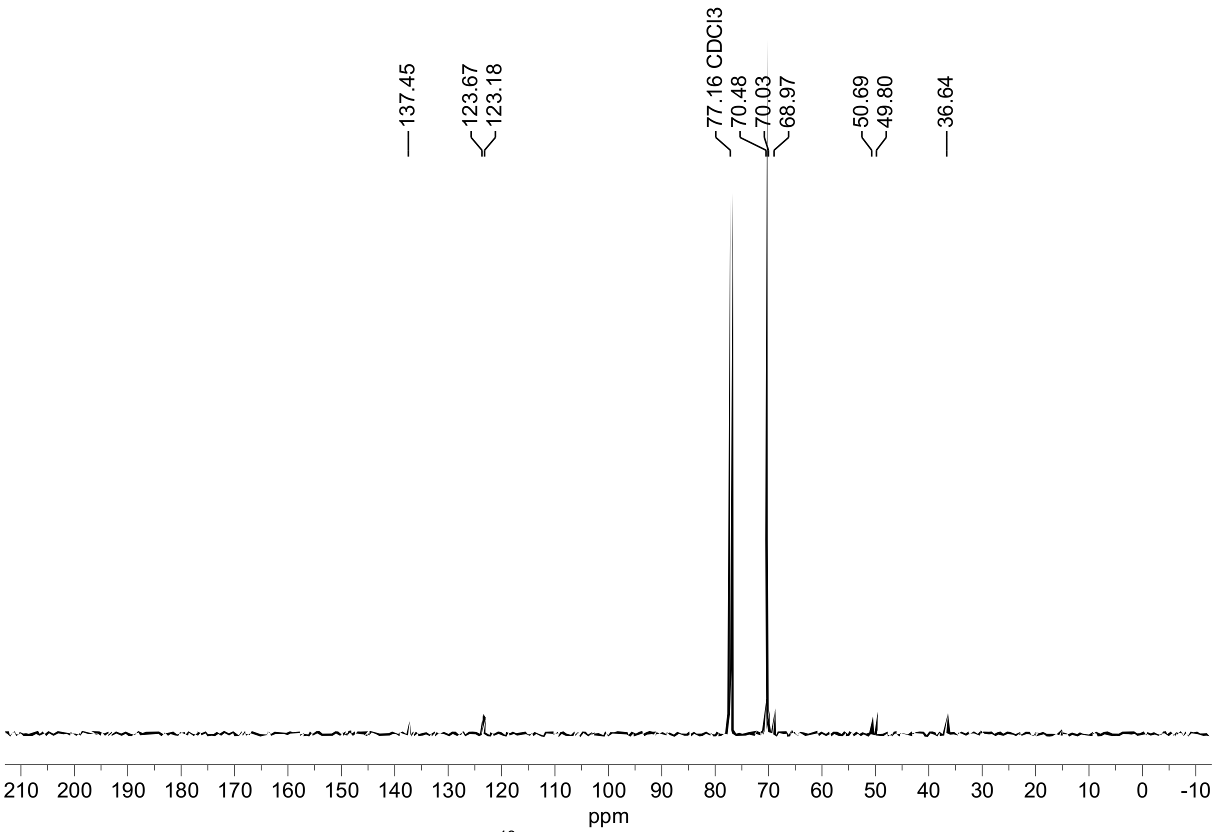

Figure S57 ^{13}C-NMR (500 MHz, $CDCl_3$) of **2**.

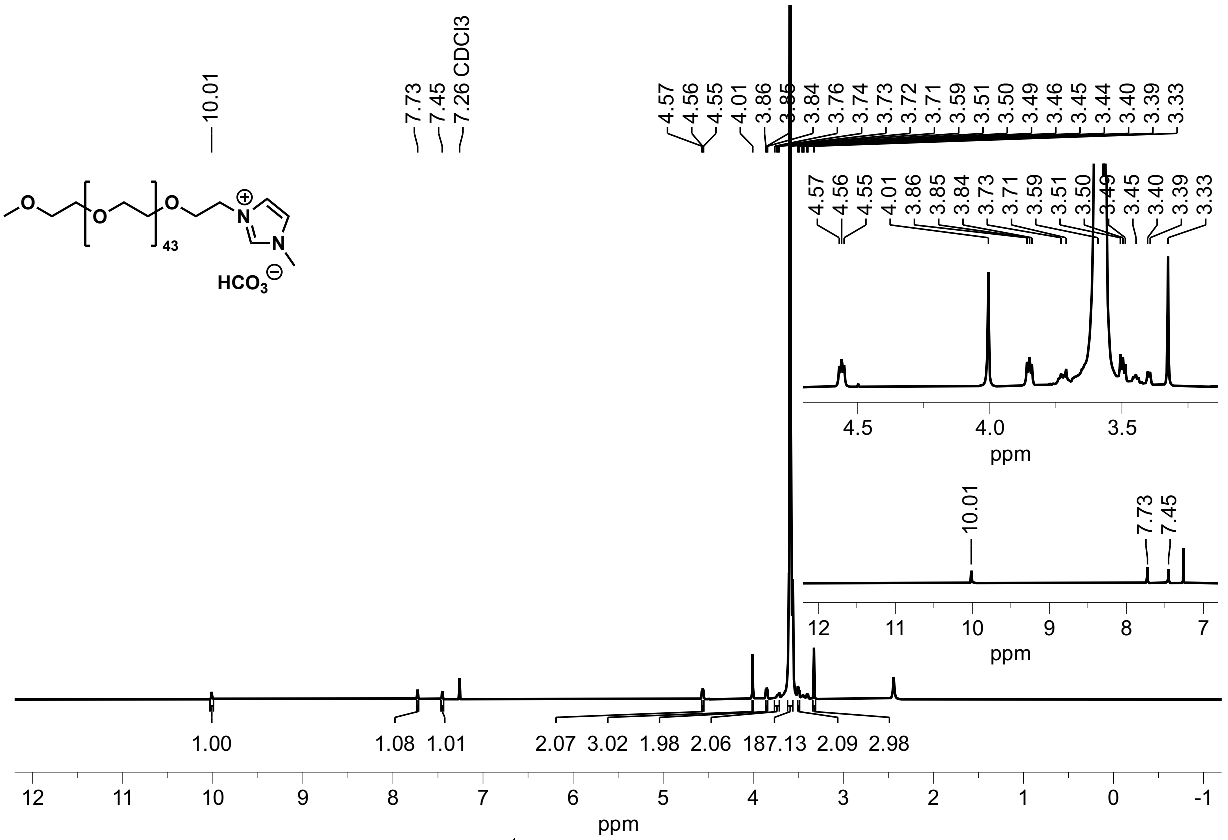

Figure S58 ^{1}H-NMR (500 MHz, $CDCl_3$) of **1'**.

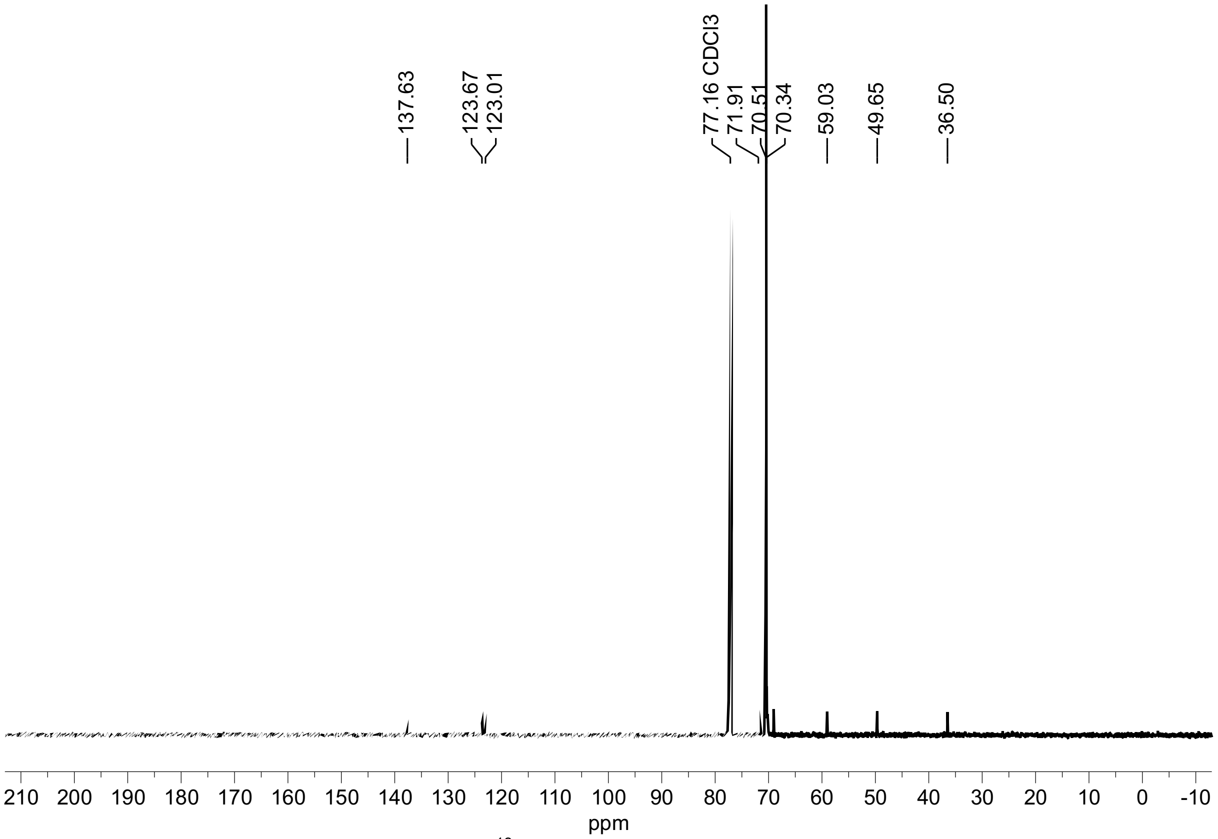

Figure S59 ^{13}C-NMR (500 MHz, $CDCl_3$) of **1'**.

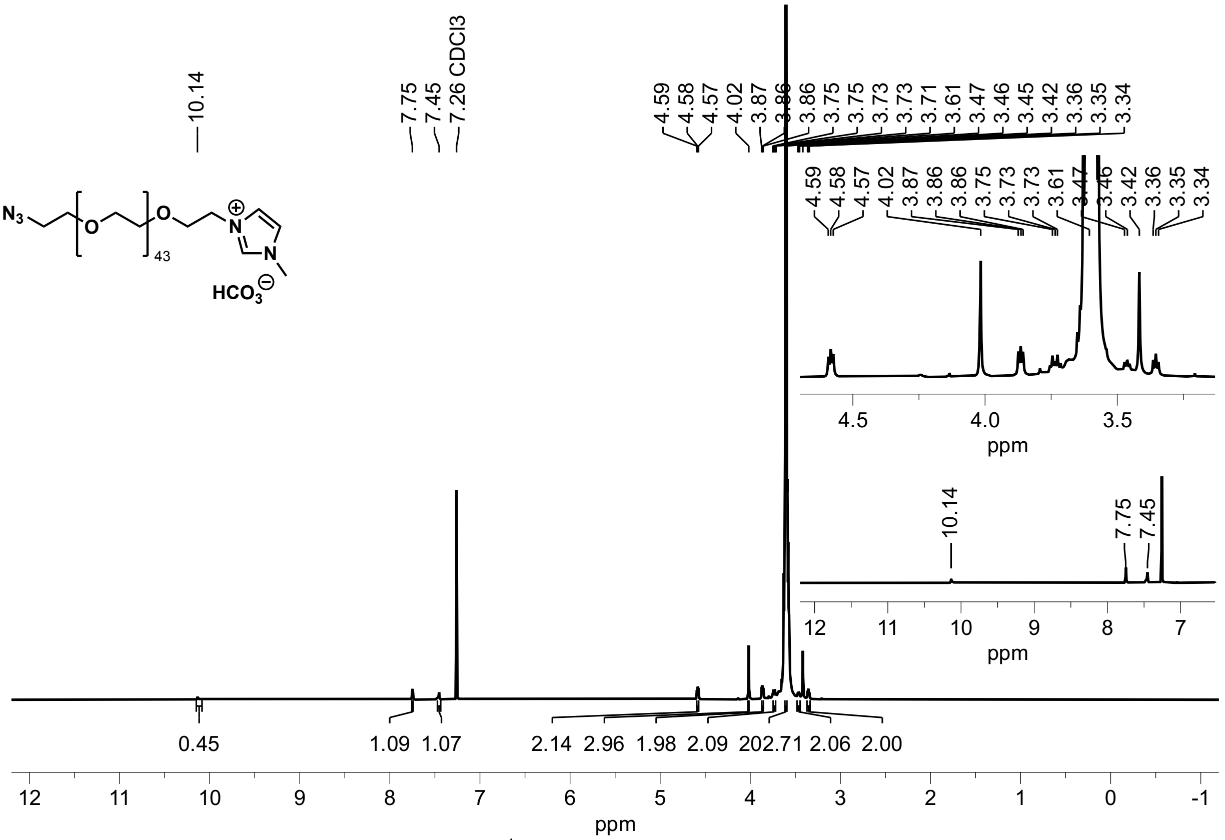

Figure S60 ^{1}H-NMR (500 MHz, $CDCl_3$) of **2'**.

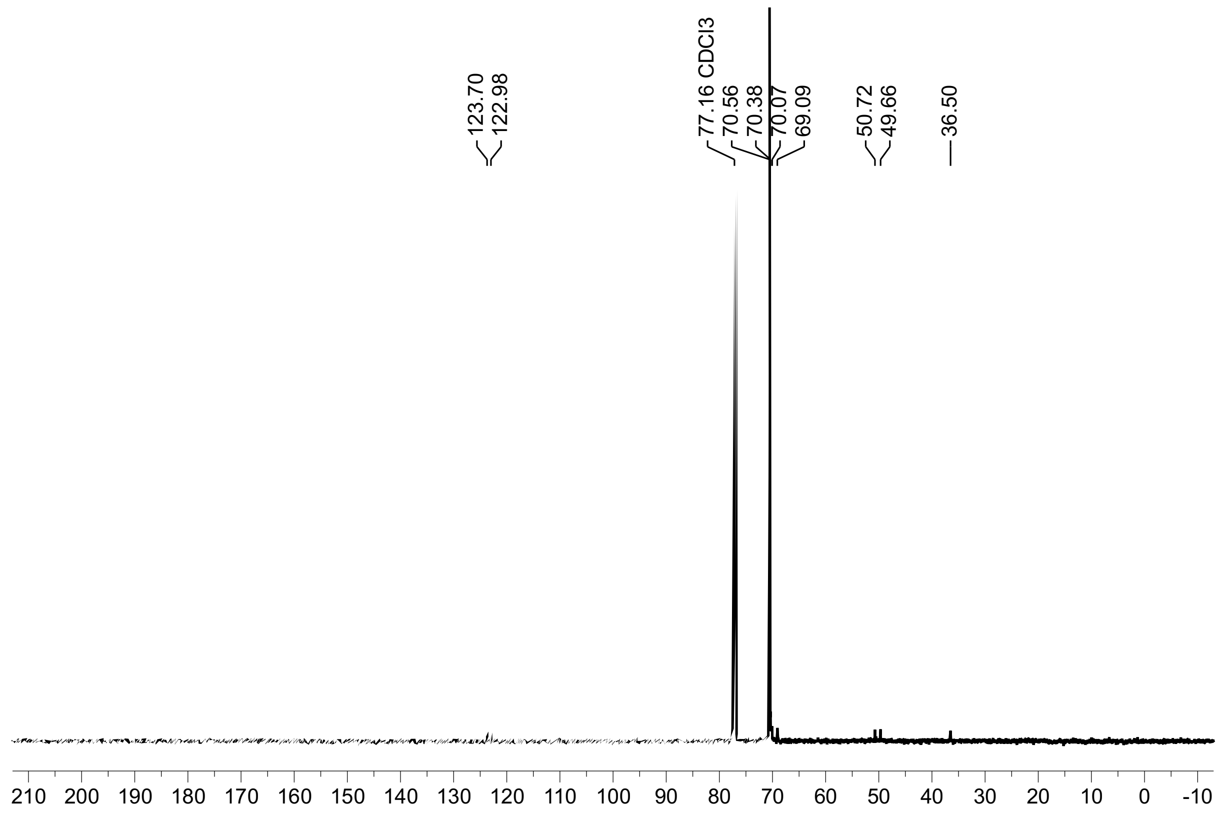

Figure S61 ^{1}H-NMR (500 MHz, $CDCl_3$) of **2'**.

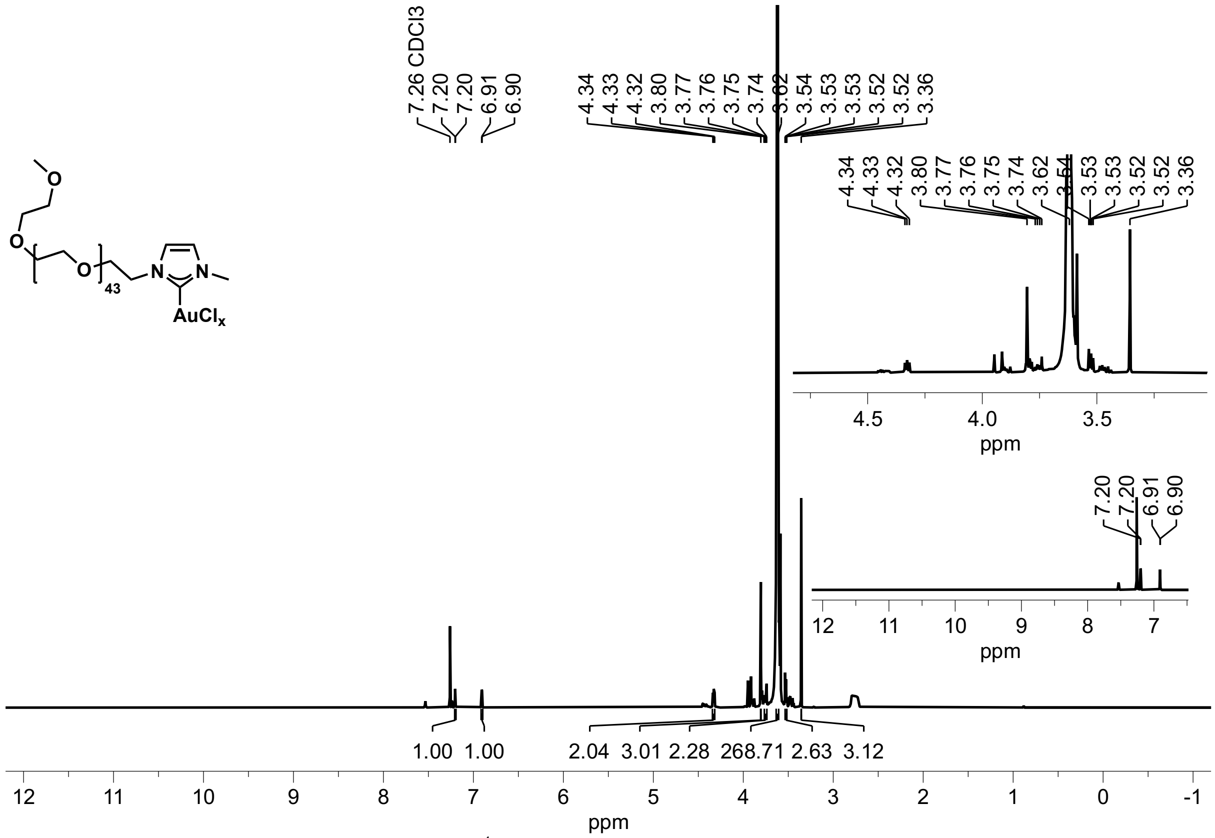

Figure S62 ^{1}H-NMR (500 MHz, $CDCl_3$) of **1-Au**.

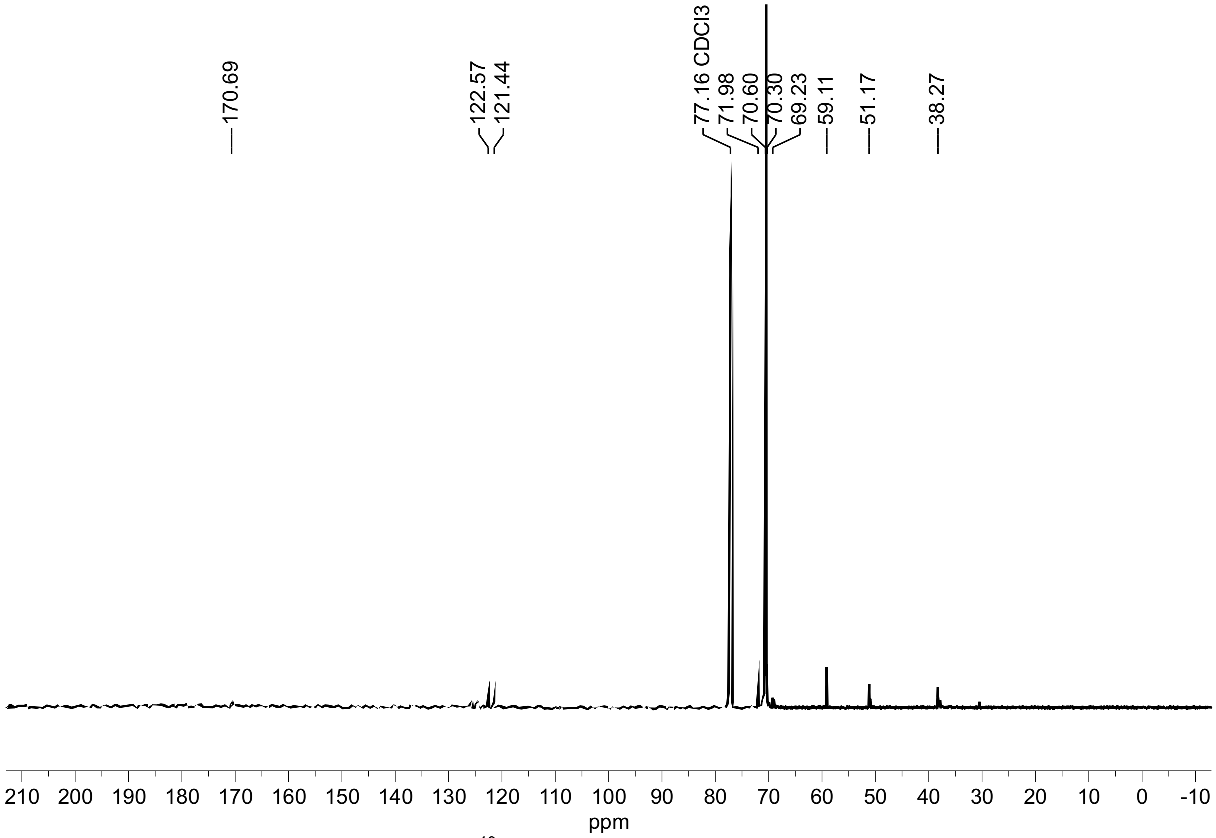

Figure S63 ^{13}C-NMR (500 MHz, $CDCl_3$) of **1-Au**.

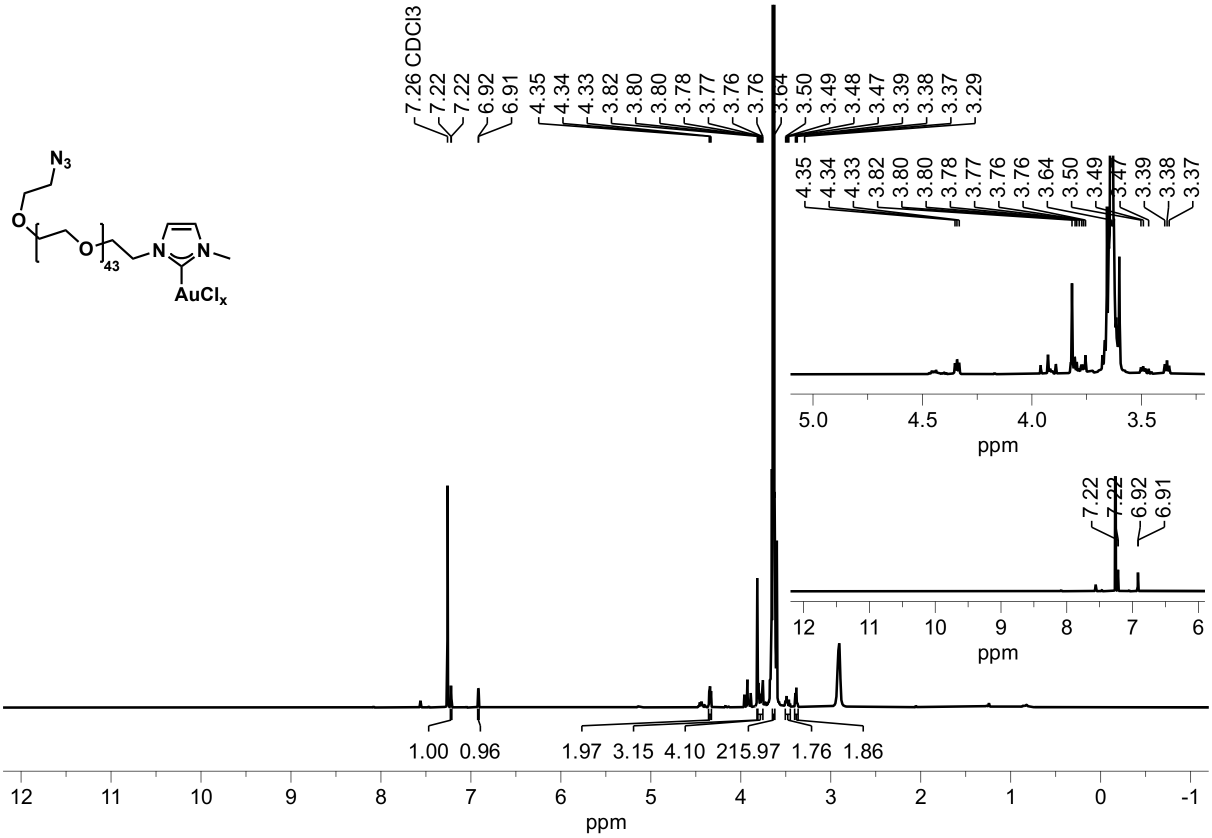

Figure S64 ^{1}H-NMR (500 MHz, $CDCl_3$) of **2-Au**.

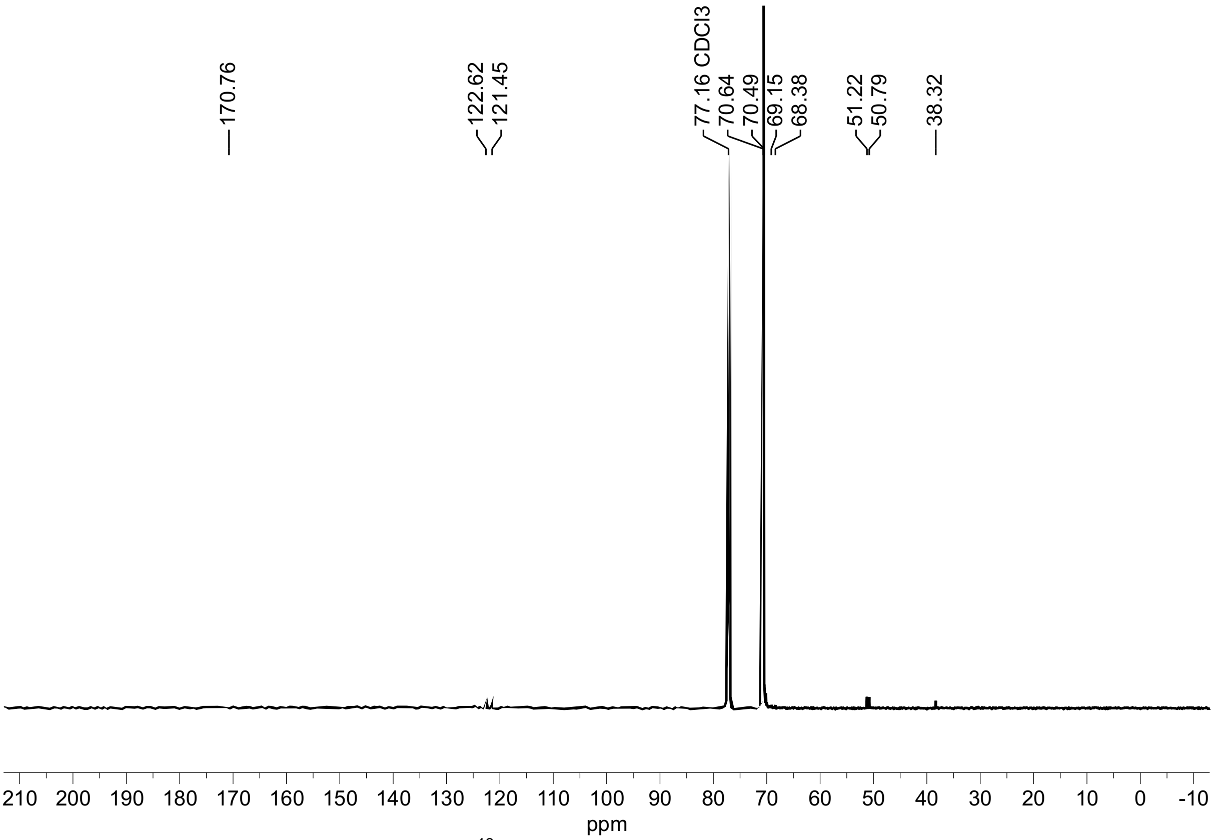

Figure S65 ^{13}C-NMR (500 MHz, $CDCl_3$) of **2-Au**.

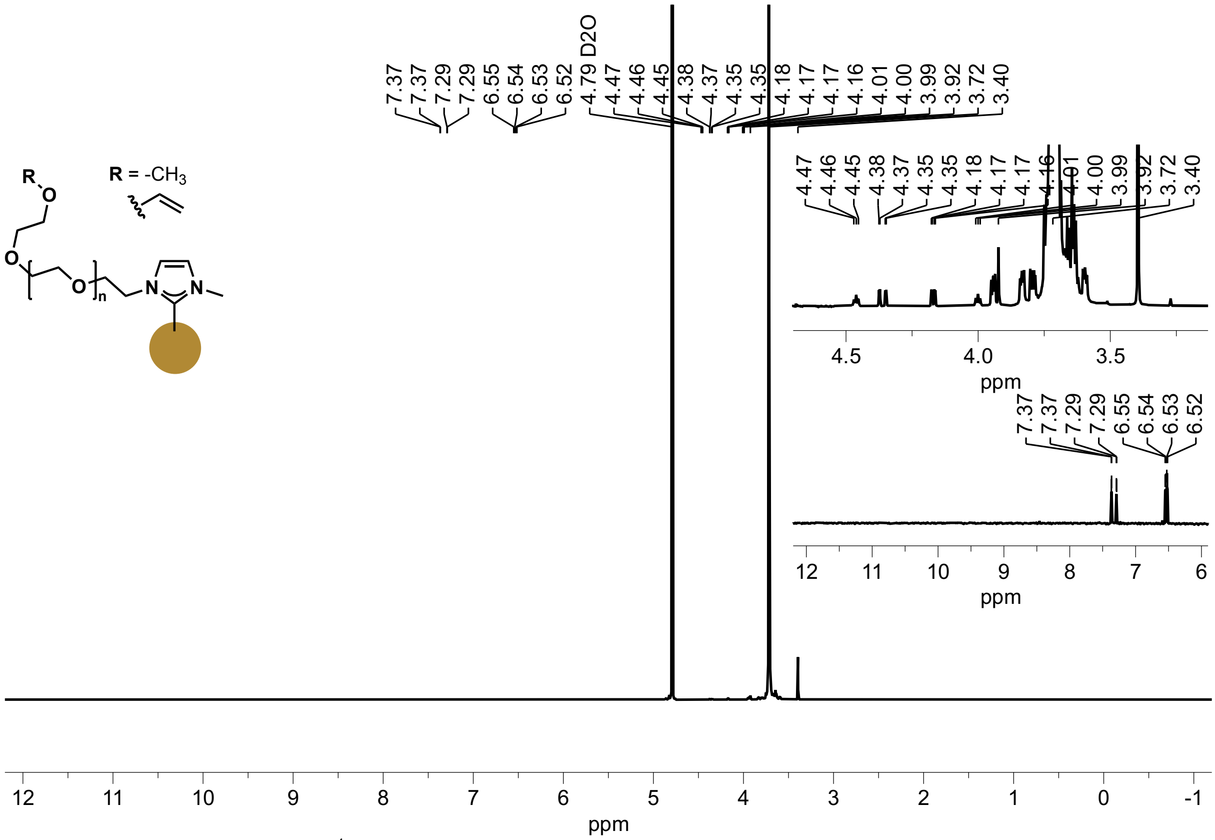

Figure S66 ^{1}H-NMR (500 MHz, D_2O) of **AuNP-1** obtained by procedure **P1**.

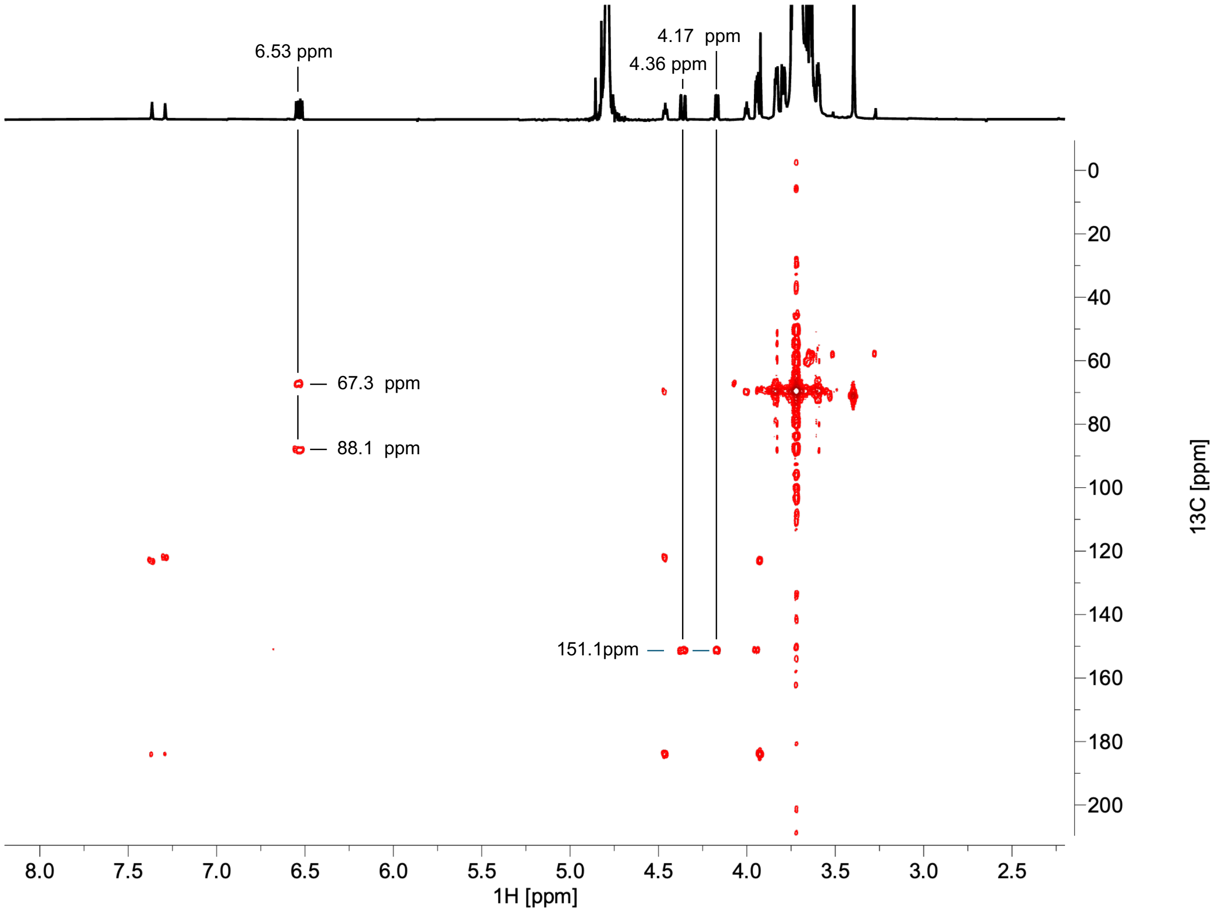

Figure S67 ^{1}H/^{13}C-HMBC (500 MHz, D_2O) of **AuNP-1** obtained by procedure **P1**.

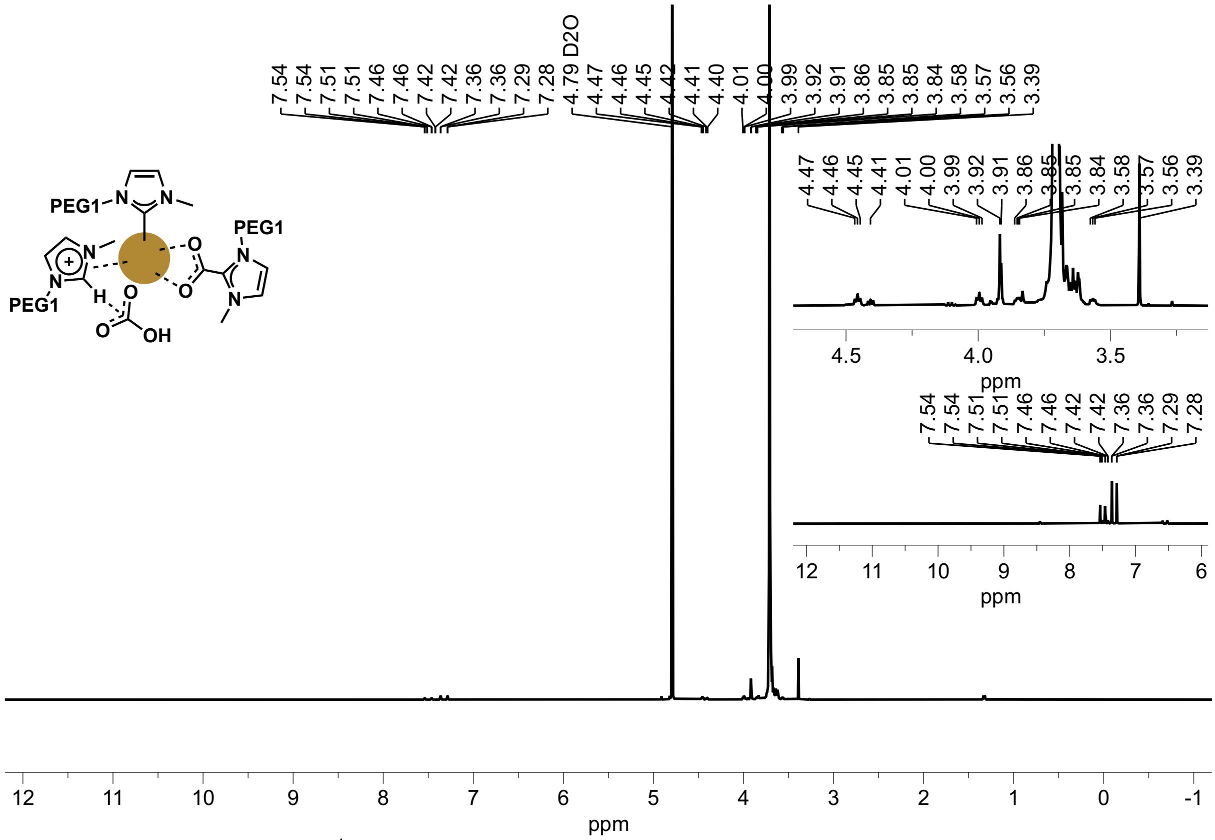

Figure S68 ^{1}H-NMR (500 MHz, D_2O) of **AuNP-2** obtained by procedure **P2**.

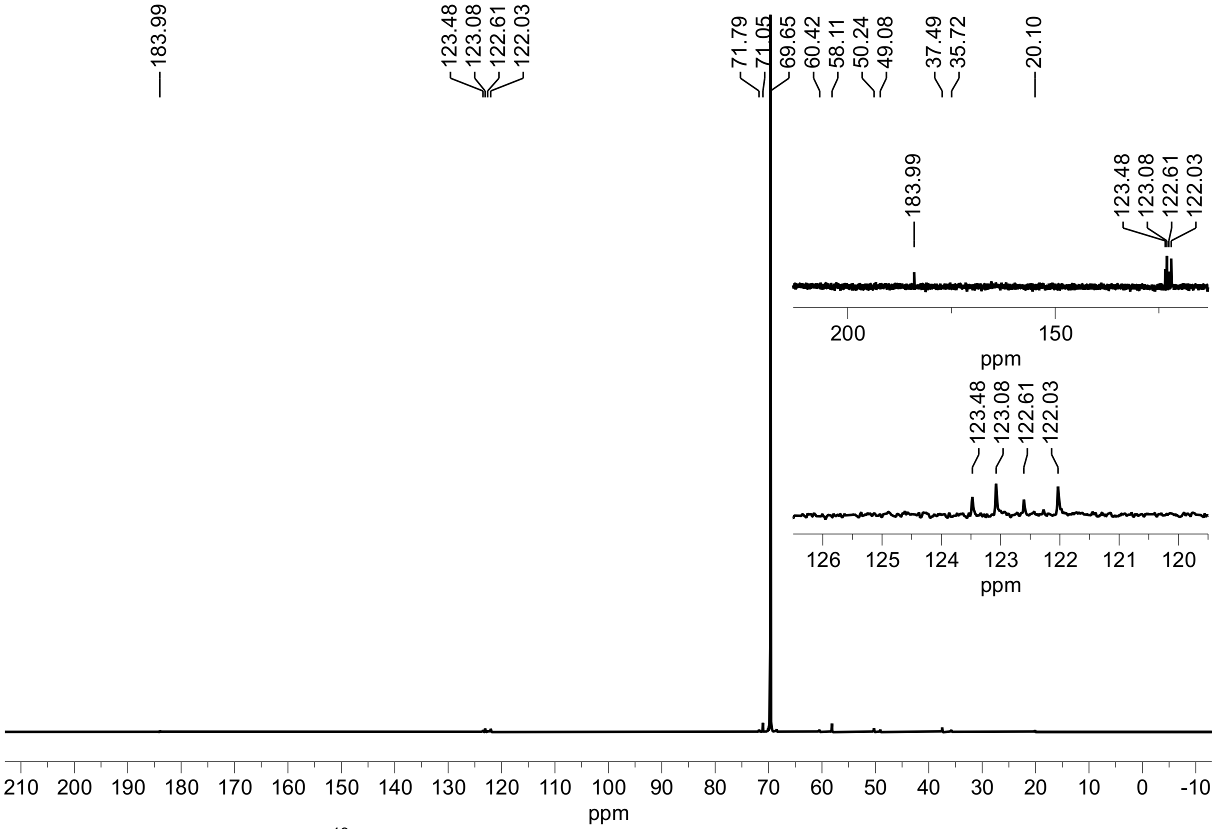

Figure S69 ^{13}C-NMR (500 MHz, D_2O) of **AuNP-2** obtained by procedure **P2**.

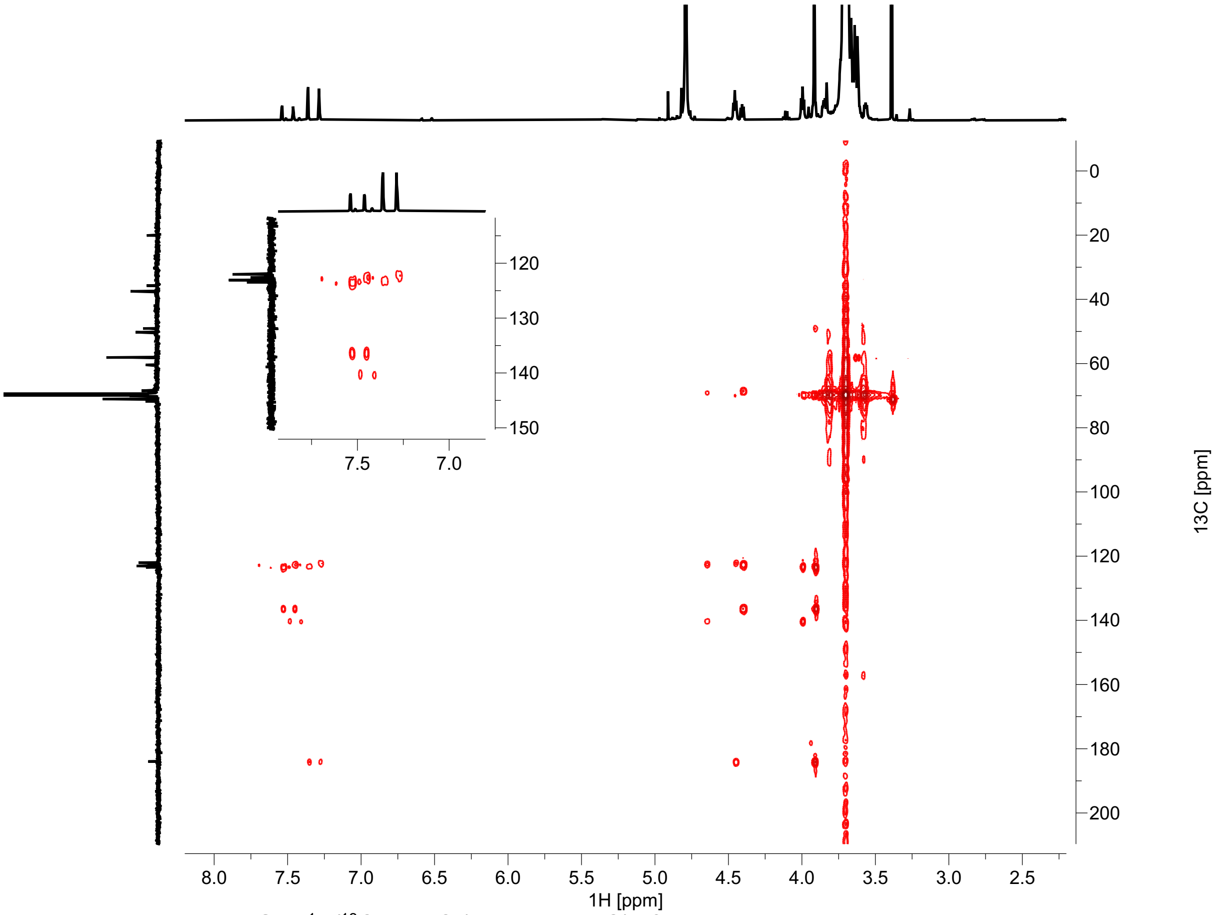

Figure S70 $^{1}H/^{13}C$-HMBC (500 MHz, D_2O) of **AuNP-2** obtained by procedure **P2**.

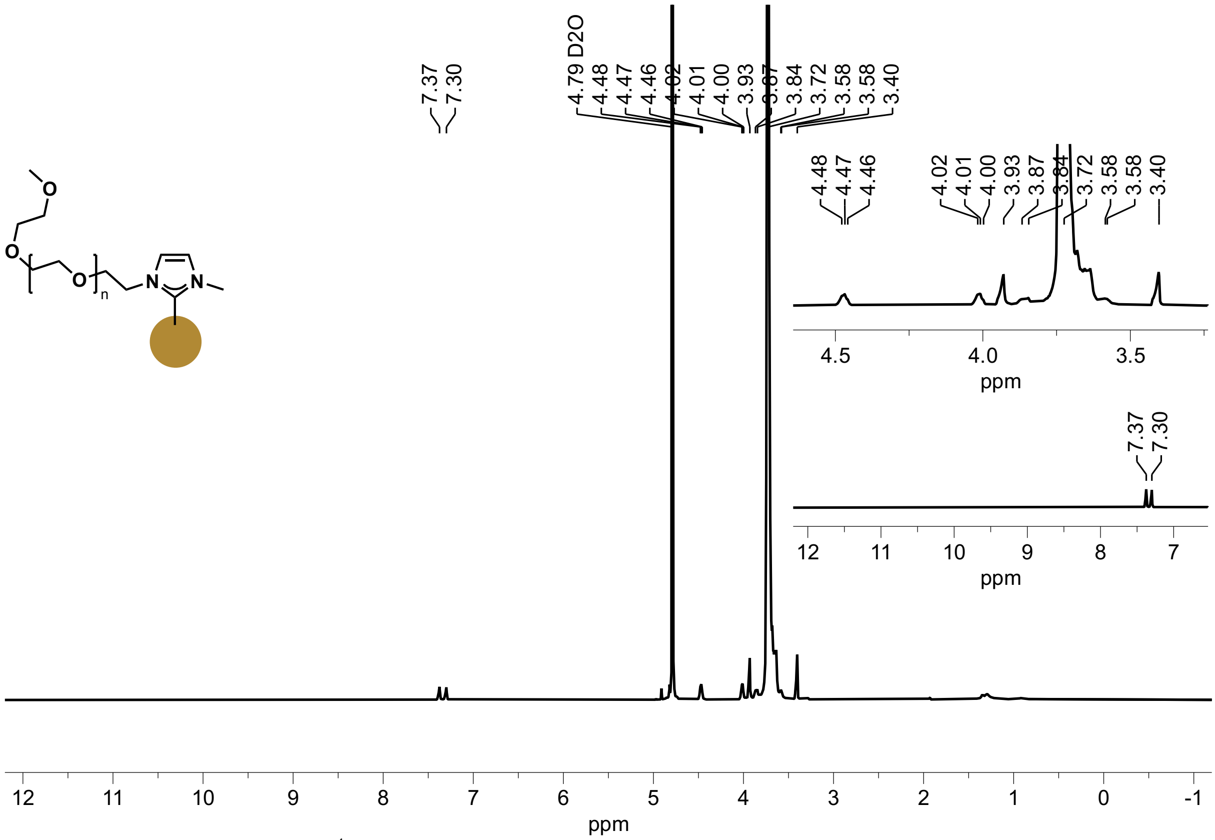

Figure S71 ^{1}H-NMR (500 MHz, D_2O) of **AuNP-3** obtained by procedure **P3**.

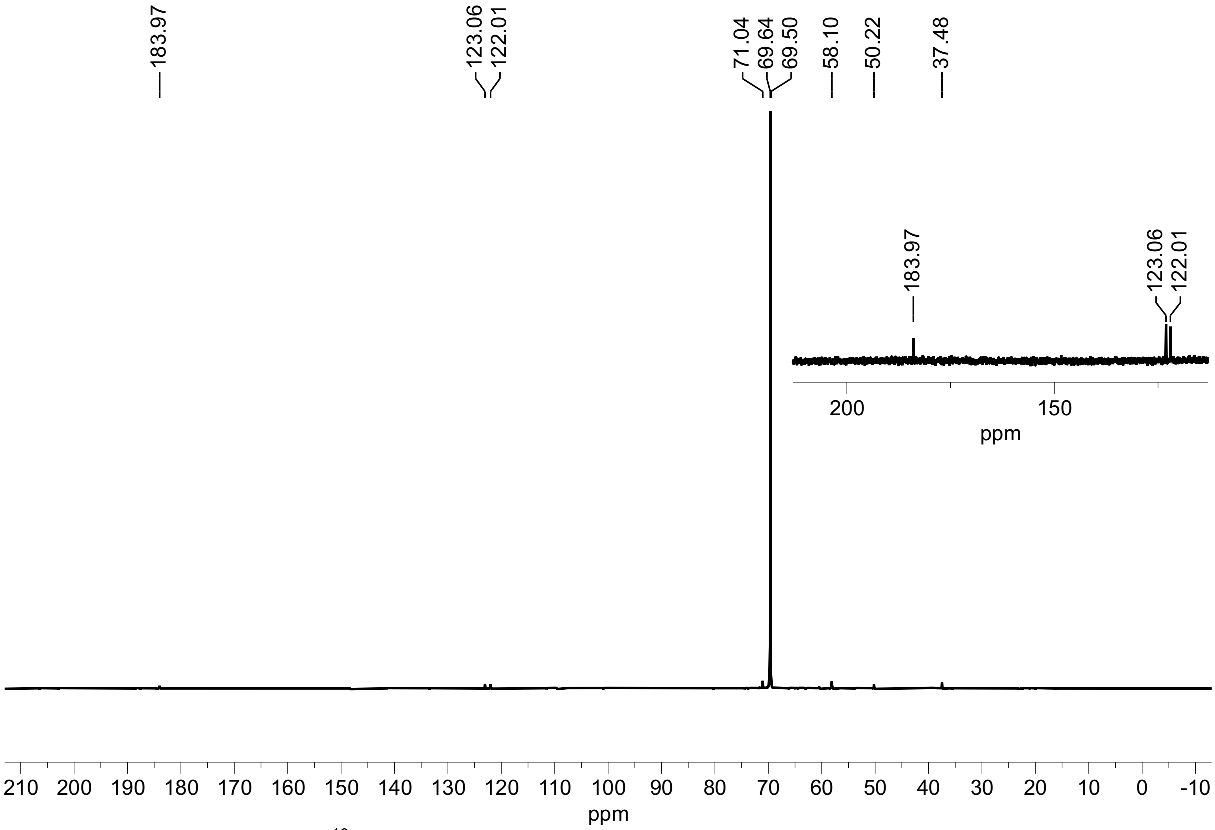

Figure S72 ^{13}C-NMR (500 MHz, D_2O) of **AuNP-3** obtained by procedure **P3**.

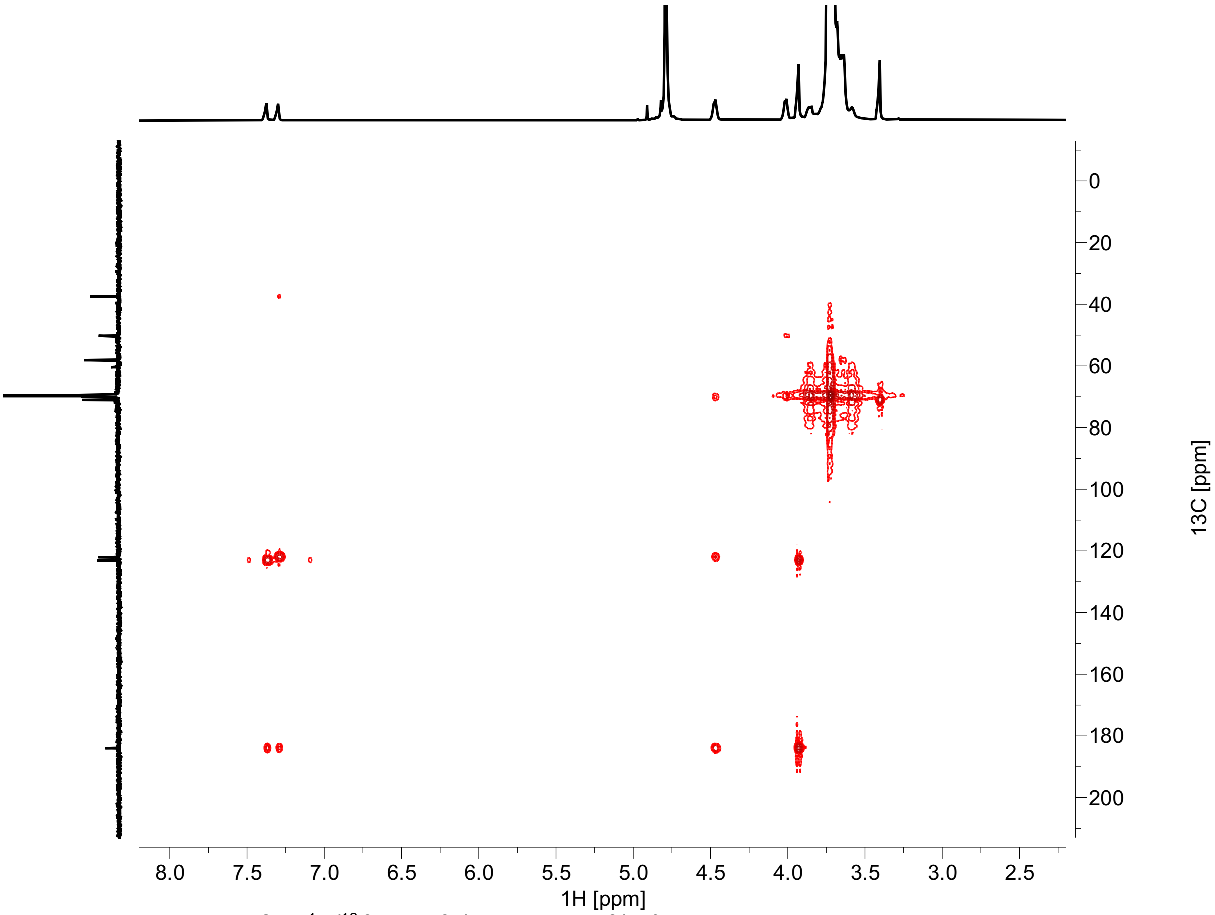

Figure S73 ^{1}H/^{13}C HMBC (500 MHz, D_2O) of **AuNP-3** obtained by procedure **P3**.

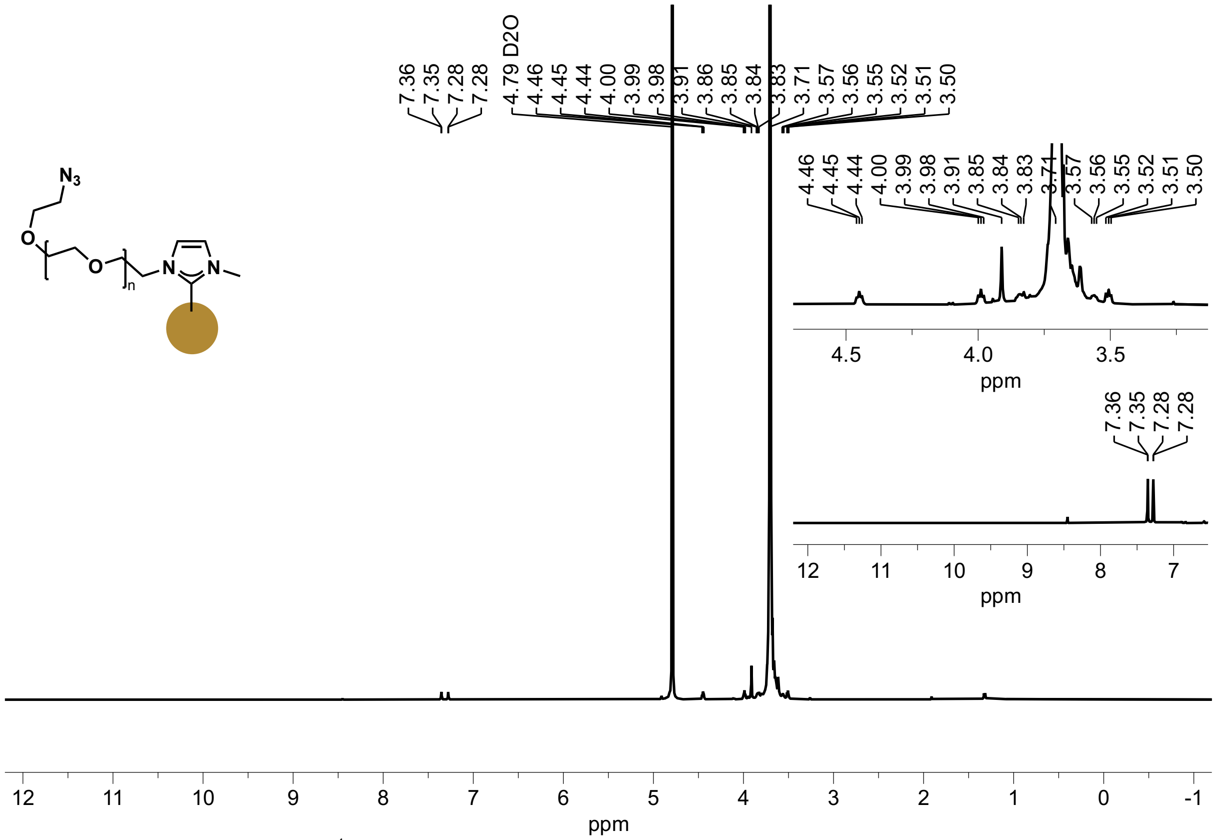

Figure S74 ^{1}H-NMR (500 MHz, D_2O) of **AuNP-4** obtained by procedure **P3**.

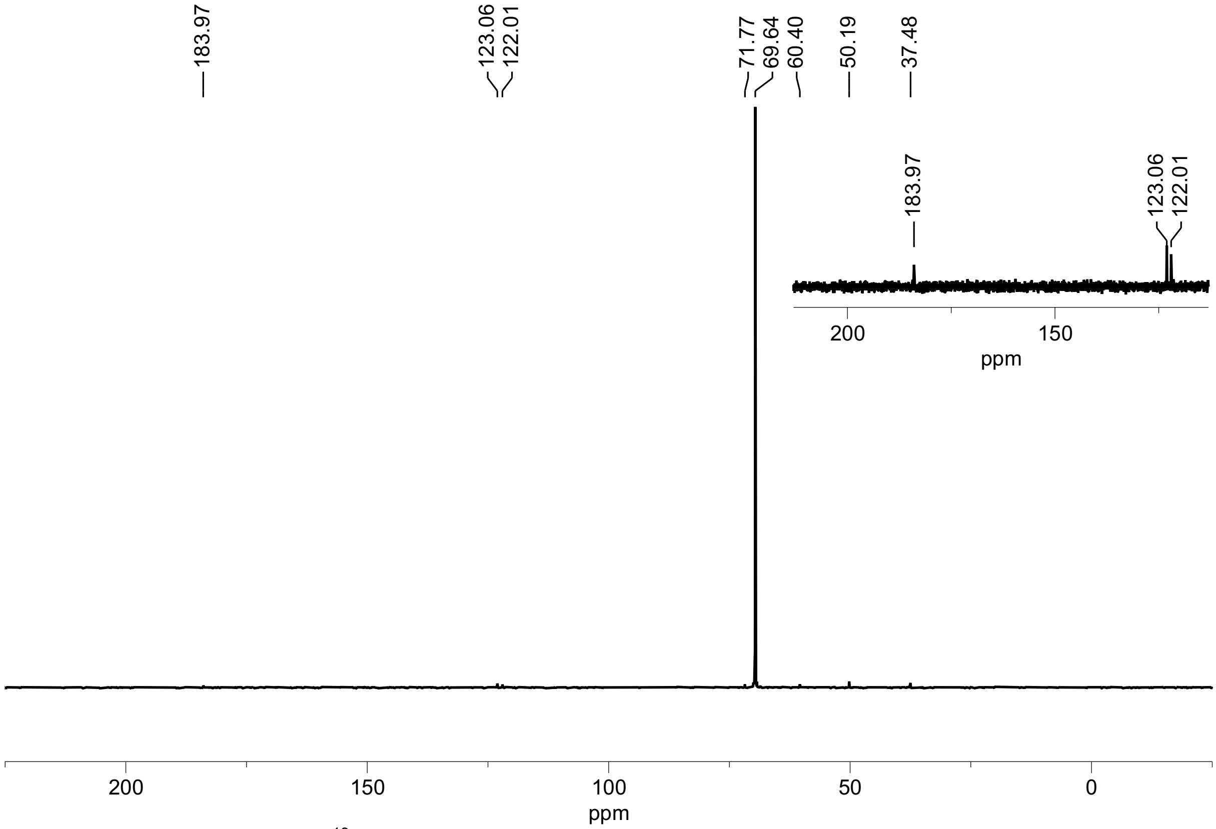

Figure S75 ^{13}C-NMR (500 MHz, D_2O) of **AuNP-4** obtained by procedure **P3**.

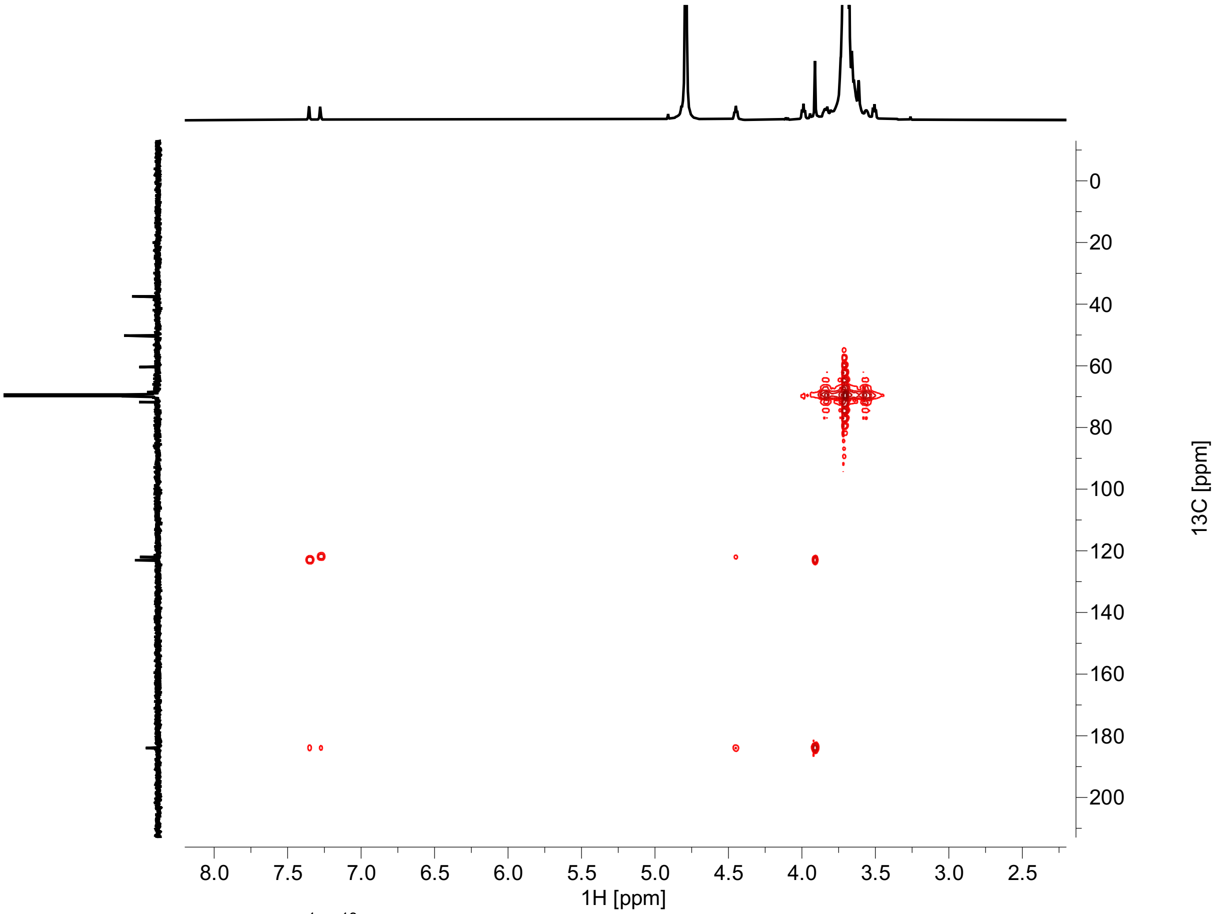

Figure S76 $^{1}H/^{13}C$-HMBC (500 MHz, D_2O) of **AuNP-4** obtained by procedure **P3**.

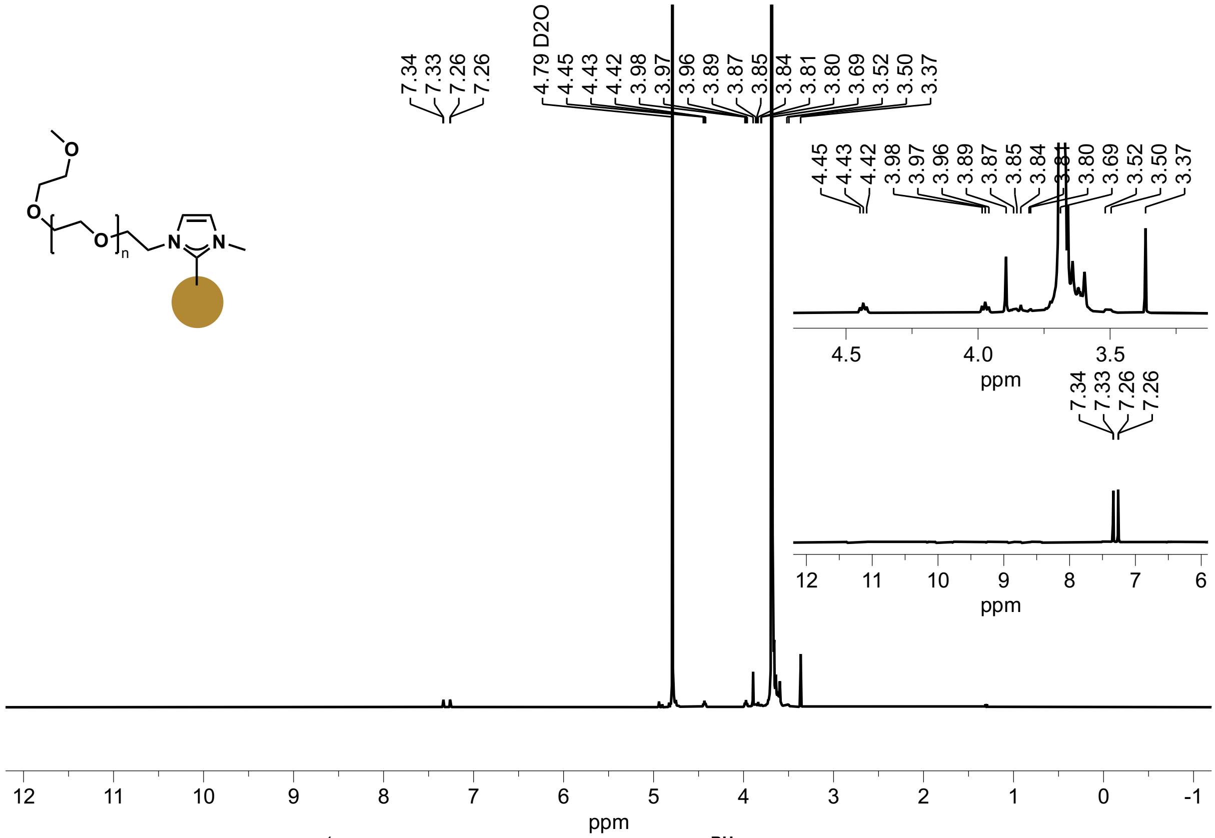

Figure S77 ^{1}H-NMR (500 MHz, D_2O) of **AuNP-3^{BU}** obtained by procedure **P5**.

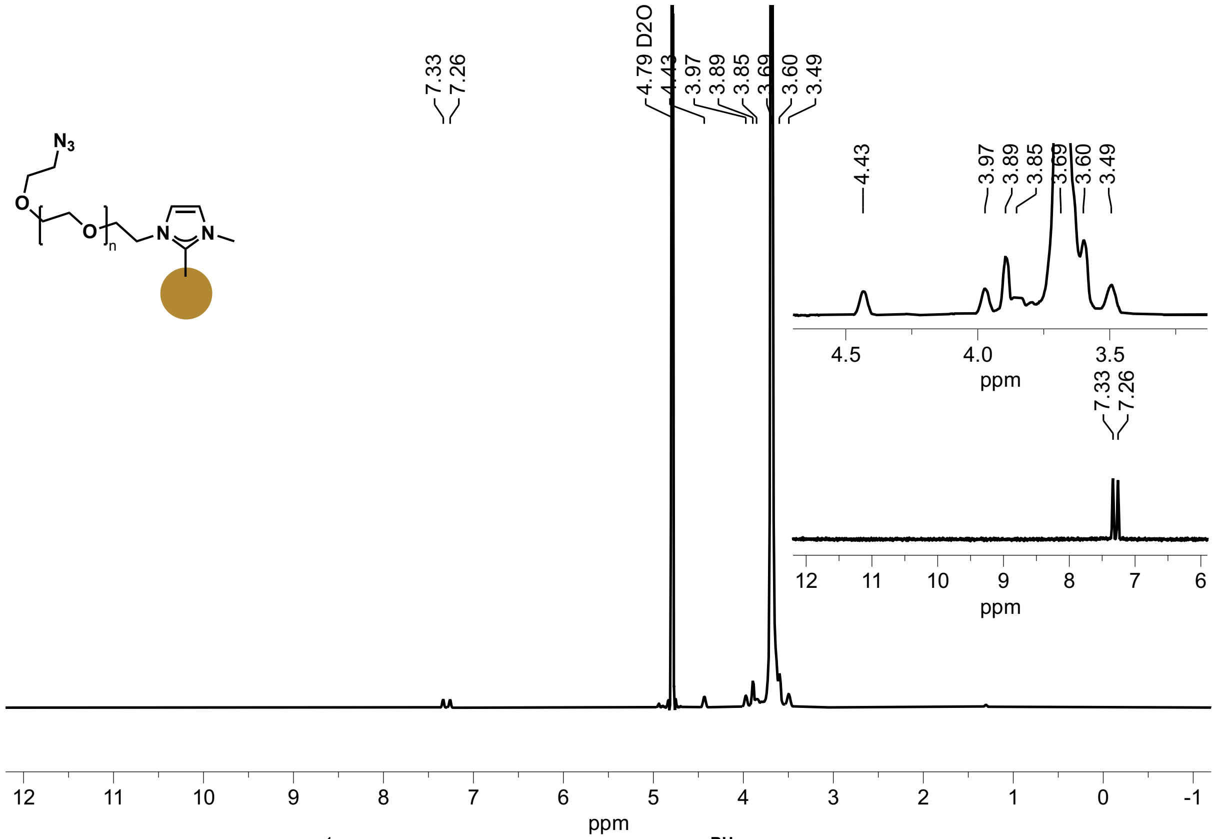

Figure S78 ^{1}H-NMR (500 MHz, D_2O) of **AuNP-4^{BU}** obtained by procedure **P5**.

High-resolution Mass Spectra

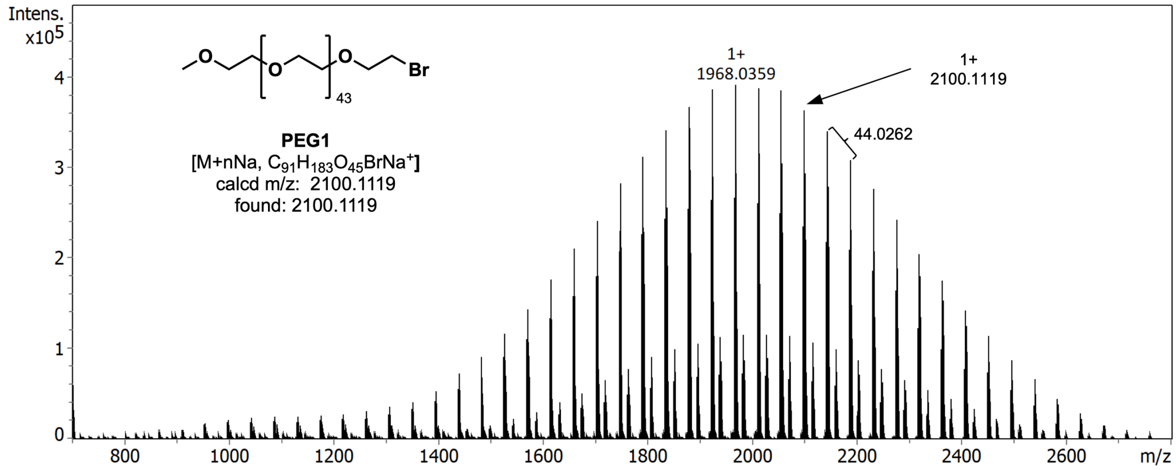

Figure S79 High-res. Maldi-TOF spectra of **PEG1**.

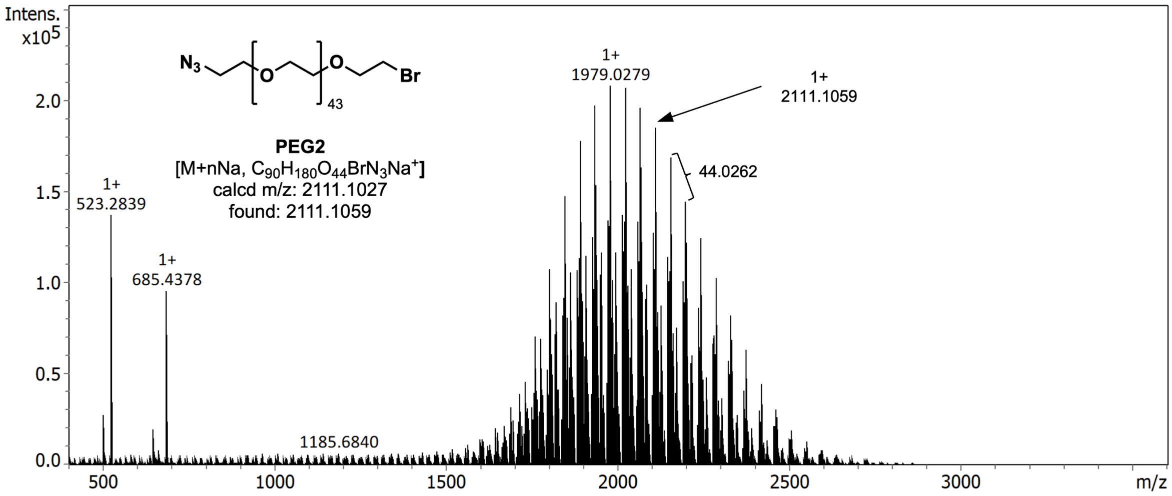

Figure S80 High-res. Maldi-TOF spectra of **PEG2**. Additional populations caused by N_3 decomposition.

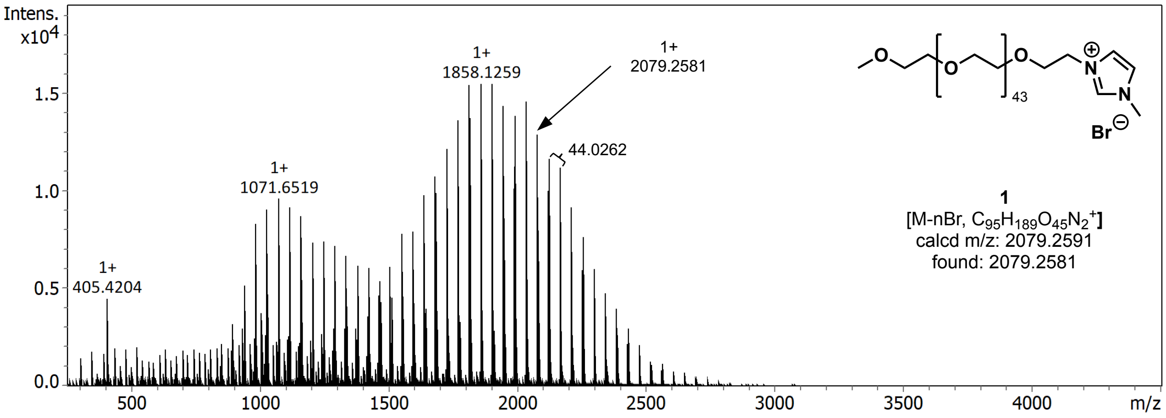

Figure S81 High-res. Maldi-TOF spectra of **1**.

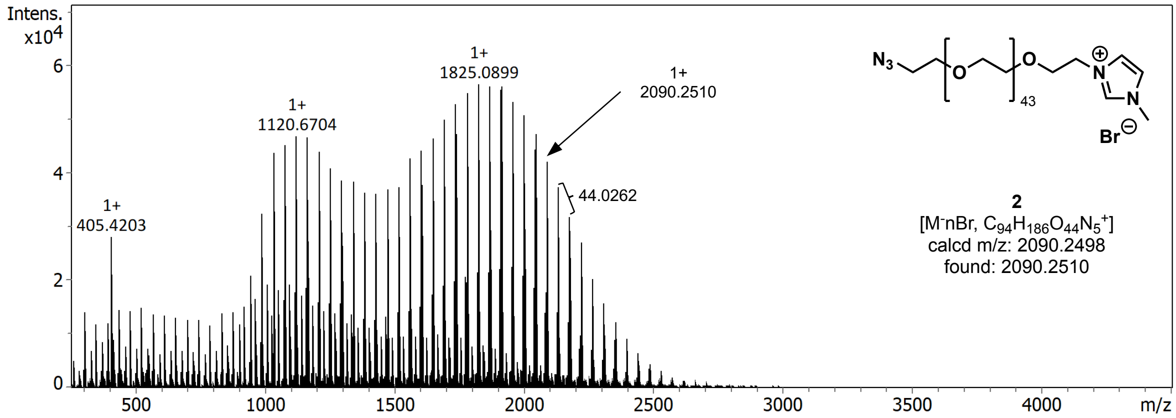

Figure S82 High-res. Maldi-TOF spectra of **2**. Additional populations caused by N_3 decomposition.

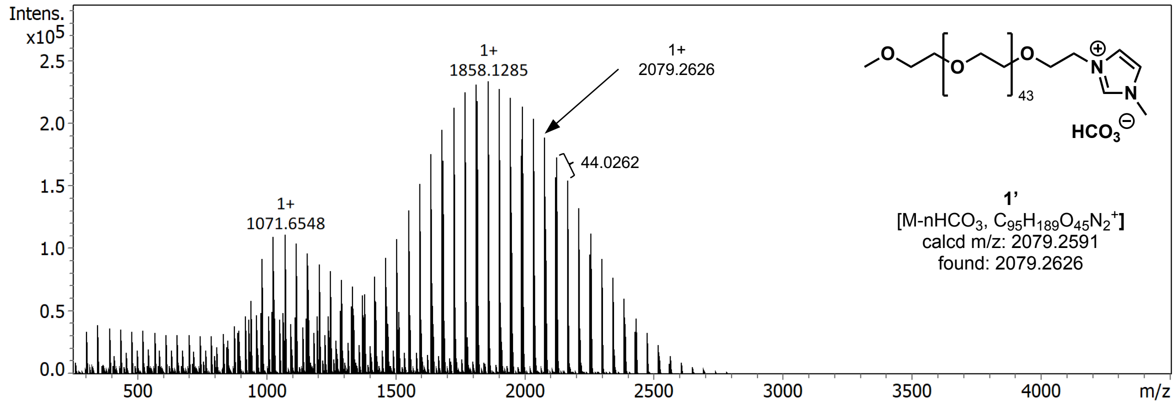

Figure 83 High-res. Maldi-TOF spectra of **1'**.

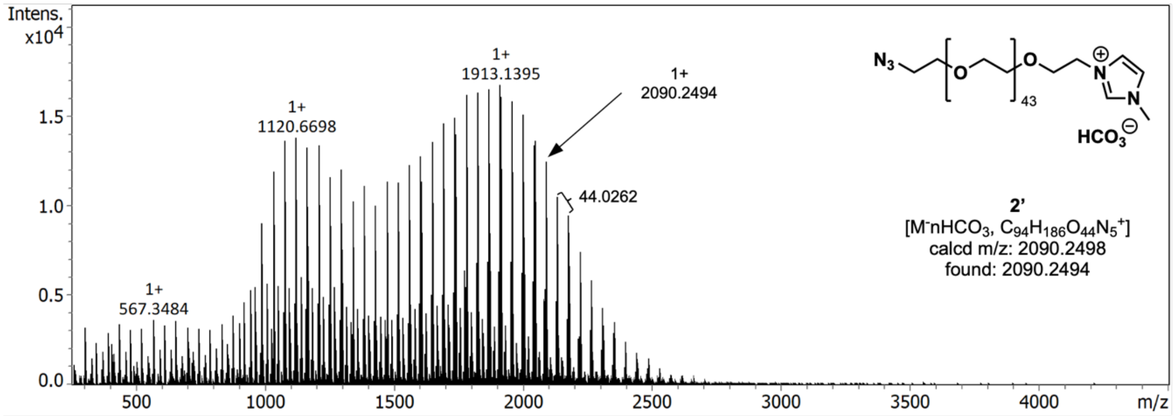

Figure S84 High-res. Maldi-TOF spectra of **2'**. Additional populations caused by N_3 decomposition.

References

[1] a) D. Velluto, D. Demurtas, J. A. Hubbell, *Mol. Pharmaceutics* **2008**, *5*, 632-642; b) J. Edward Semple, B. Sullivan, T. Vojkovsky, K. N. Sill, *J. Polym. Sci., Part A: Polym. Chem.* **2016**, *54*, 2888-2895.

[2] V. H. Jadhav, H. J. Jeong, S. T. Lim, M. H. Sohn, D. W. Kim, *Org. Lett.* **2011**, *13*, 2502-2505.

[3] M. Fevre, J. Pinaud, A. Leteneur, Y. Gnanou, J. Vignolle, D. Taton, K. Miqueu, J. M. Sotiropoulos, *J. Am. Chem. Soc.* **2012**, *134*, 6776-6784.

[4] M. J. MacLeod, J. A. Johnson, *J. Am. Chem. Soc.* **2015**, *137*, 7974-7977.

[5] S. Peng, Y. Lee, C. Wang, H. Yin, S. Dai, S. Sun, *Nano Res.* **2008**, *1*, 229-234.

[6] G. Socrates, *Infrared and Raman Characteristic Group Frequencies: Tables and Charts*, John Wiley & Sons Ltd., Chichester, **2004**.

[7] Y. Katayama, F. Nattino, L. Giordano, J. Hwang, R. R. Rao, O. Andreussi, N. Marzari, Y. Shao-Horn, *J. Phys. Chem. C* **2018**, *123*, 5951-5963.

[8] M. J. Trujillo, S. L. Strausser, J. C. Becca, J. F. DeJesus, L. Jensen, D. M. Jenkins, J. P. Camden, *J. Phys. Chem. Lett.* **2018**, *9*, 6779-6785.

[9] A. J. Young, M. Sauer, G. Rubio, A. Sato, A. Foelske, C. J. Serpell, J. M. Chin, M. R. Reithofer, *Nanoscale* **2019**, *11*, 8327-8333.

[10] D. T. H. Nguyen, M. Bélanger-Bouliga, L. R. Shultz, A. Maity, T. Jurca, A. Nazemi, *Chem. Mater.* **2021**, *33*, 9588-9600.

[11] K. Yamagishi, K. Sawaki, A. Murata, S. Takeoka, *Chem. Commun.* **2015**, *51*, 7879-7882.
